# Validation of a Point-of-Care Optical Coherence Tomography Device with Machine Learning Algorithm for Detection of Oral Potentially Malignant and Malignant Lesions

**DOI:** 10.3390/cancers13143583

**Published:** 2021-07-17

**Authors:** Bonney Lee James, Sumsum P. Sunny, Andrew Emon Heidari, Ravindra D. Ramanjinappa, Tracie Lam, Anne V. Tran, Sandeep Kankanala, Shiladitya Sil, Vidya Tiwari, Sanjana Patrick, Vijay Pillai, Vivek Shetty, Naveen Hedne, Darshat Shah, Nameeta Shah, Zhong-ping Chen, Uma Kandasarma, Subhashini Attavar Raghavan, Shubha Gurudath, Praveen Birur Nagaraj, Petra Wilder-Smith, Amritha Suresh, Moni Abraham Kuriakose

**Affiliations:** 1 Integrated Head and Neck Oncology Program (DSRG-5), Mazumdar Shaw Center for Translational Research (MSCTR), Mazumdar Shaw Medical Foundation, NH Health City, Bangalore 560099, India; bonney.lee.james@ms-mf.org (B.L.J.); sumsumsp@gmail.com (S.P.S.); ravindradr88@gmail.com (R.D.R.); praveen.birur@gmail.com (P.B.N.); 2 Manipal Academy of Higher Education (MAHE), Karnataka 576104, India; 3 Department of Head and Neck Oncology, Mazumdar Shaw Medical Center, NH Health City, Bangalore 560099, India; drvijaypillai@gmail.com (V.P.); vivek.shetty.dr@narayanahealth.org (V.S.); hednenaveen@gmail.com (N.H.); 4 Beckman Laser Institute, UCI, Irvine, CA 92612, USA; aheidari.uci@gmail.com (A.E.H.); tracielam.m@gmail.com (T.L.); annevt@uci.edu (A.V.T.); z2chen@uci.edu (Z.-p.C.); pwsmith@uci.edu (P.W.-S.); 5 Department of Oral Medicine and Radiology, KLE Society’s Institute of Dental Sciences, Bangalore 560022, India; kankanala.sandeep86@gmail.com (S.K.); shiladitya.sil@gmail.com (S.S.); subhashiniar@gmail.com (S.A.R.); drshubha.gurudath@gmail.com (S.G.); 6 Biocon Foundation, Bangalore 560100, India; vidyatiwari96@gmail.com (V.T.); tanjupat@yahoo.com (S.P.); 7 Mazumdar Shaw Center for Translational Research (MSCTR), Mazumdar Shaw Medical Foundation, NH Health City, Bangalore 560099, India; dshahms@hotmail.com (D.S.); nameeta.shah@ms-mf.org (N.S.); 8 Department of Oral and Maxillofacial Pathology, KLE Society’s Institute of Dental Sciences, Bangalore 560022, India; umak235@gmail.com

**Keywords:** optical coherence tomography, oral cancer, oral squamous cell carcinoma, oral potentially malignant lesions, pre-malignant lesions, artificial neural network

## Abstract

**Simple Summary:**

Early detection is crucial towards improving survival in patients diagnosed with oral cancer. Non-invasive strategies equivalent to histology diagnosis are extremely valuable in oral cancer screening and early detection in resource-constrained settings. Optical coherence tomography (OCT), an optical biopsy technique enables real-time imaging with periodic surveillance and capability to image architectural features of the tissues. We report that while OCT system delineates oral pre-cancer and cancer with more than 90% sensitivity, integration, with artificial neural network-based analysis efficiently identifies high-risk, oral pre-cancer (83%). This study provides evidence that the robust, low-cost system was effective as a point-of-care device in resource-constrained settings. The high accuracy and portability signify widespread clinical application in oral cancer screening and/or surveillance.

**Abstract:**

Non-invasive strategies that can identify oral malignant and dysplastic oral potentially-malignant lesions (OPML) are necessary in cancer screening and long-term surveillance. Optical coherence tomography (OCT) can be a rapid, real time and non-invasive imaging method for frequent patient surveillance. Here, we report the validation of a portable, robust OCT device in 232 patients (lesions: 347) in different clinical settings. The device deployed with algorithm-based automated diagnosis, showed efficacy in delineation of oral benign and normal (*n* = 151), OPML (*n* = 121), and malignant lesions (*n* = 75) in community and tertiary care settings. This study showed that OCT images analyzed by automated image processing algorithm could distinguish the dysplastic-OPML and malignant lesions with a sensitivity of 95% and 93%, respectively. Furthermore, we explored the ability of multiple (*n* = 14) artificial neural network (ANN) based feature extraction techniques for delineation high grade-OPML (moderate/severe dysplasia). The support vector machine (SVM) model built over ANN, delineated high-grade dysplasia with sensitivity of 83%, which in turn, can be employed to triage patients for tertiary care. The study provides evidence towards the utility of the robust and low-cost OCT instrument as a point-of-care device in resource-constrained settings and the potential clinical application of device in screening and surveillance of oral cancer.

## 1. Introduction

Early detection and subsequent intervention are the best approach for improving the outcome of oral cancer. Cancers of the oral cavity and lips account for approximately 377,713 new cancer cases with a mortality of approximately 177,757 cases worldwide [1]. India faces the highest age-adjusted incidence rate (15.2) and mortality rate (9.3), which could be attributed to the usage of tobacco and tobacco-related products [2,3,4,5]. More than 90% of the oral cavity cancers are oral squamous cell carcinoma (OSCC). The high morbidity and mortality in patients with OSCC are primarily attributed to late diagnosis, with more than two-thirds of OSCC cases diagnosed at an advanced stage. Prognosis of OSCC is stage-dependent, with an average five-year disease-free survival rate of 80–90% if diagnosed at early stages; and 20–30% if diagnosed at late stages [6,7].

OSCC is frequently preceded by oral potentially malignant lesions (OPML) and 1.5–20% of these lesions progress over 5 years [8]. The mechanism of malignant transformation remains unclear, with no definite prognostic markers capable of determining risk in an individual patient [9]. Patients with OPMLs, hence, need to be detected early with frequent and vigilant surveillance. Currently, monitoring is performed by visual examination followed by incisional biopsy. Studies have proven that improved awareness and reduced mortality can be achieved in high-risk patients by frequent oral screening [10] and mouth self-examination [11], nevertheless, the diagnostic accuracy of visual examination is unreliable due to inaccurate distinction of the normal variations/benign oral lesions from potentially malignant lesions [12,13]. Visual examination is also the primary approach in various screening programs involving primary health care centers; however, lack of trained frontline health workers add to delayed diagnosis [12]. Furthermore, poor compliance in high-risk patients when referred to tertiary care centers for biopsy and the need for high-resource environment including specialist expertise for biopsy confirmation add to the challenge [8]. The key approach in reducing the mortality and morbidity of OSCC is hence to generate non-invasive strategies that can detect OSCC at an early stage and enable periodical monitoring of malignant progression. 

Diagnostic adjuncts such as toluidine blue staining, brush biopsy, chemiluminescence, narrow-band imaging, and auto-fluorescence have been explored for their utility in screening high-risk patients; however, these methods were subjective and required skilled medical practitioners for interpretation [14,15]. Optical coherence tomography (OCT) is a non-invasive modality, which uses low coherence light in the near-infrared spectral range with a penetration depth of several hundred microns in tissue and has been explored to detect micro-architectural changes in tissues [16] including the epithelial thickness of the different oral sub-sites [17,18,19]. OCT-based imaging has the potential to be used as a screening tool in ex-vivo studies, wherein trained observers interpreted the images with a high efficiency (>80% accuracy) in identifying OSCC and OPMLs [20,21,22]. These studies, however, indicated the need for expert interpretation, which could give rise to subjectivity. Our group had previously reported the development of a portable, robust, low-cost OCT imaging system and an image processing algorithm for delineation of oral suspicious lesions [23]. In this study, we report the integration of OCT imaging with automated image processing and deep learning to reduce the subjectivity in image interpretation, and it is large-scale, in-vivo, validation in the delineation of OSCC and dysplastic lesions from normal/benign lesions in both community and tertiary care settings. 

## 2. Materials and Methods

### 2.1. Study Population and Design

**Figure 1:**
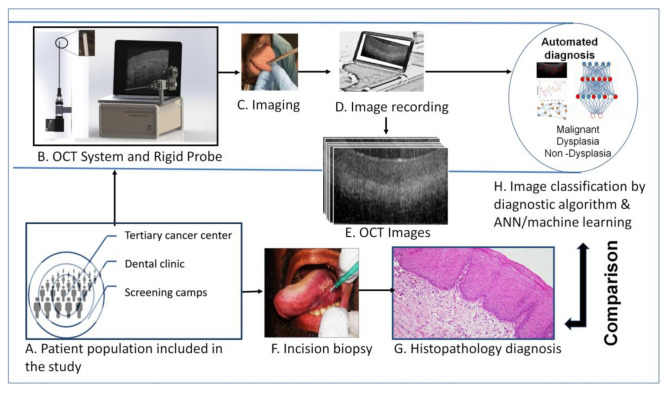
Study design. The subjects were: (**A**) recruited from low resource settings (oral cancer screening camps), dental hospitals and tertiary cancer center. (**B**) A portable Optical Coherence Tomography (OCT) system, (**C**)was used to capture oral mucosal lesion images, after oral physician consultation. The OCT images were recorded in laptop and used for image pre-processing and automated image analysis (**D**,**E**). The subjects underwent incision/excision biopsy (if indicated) for histopathological diagnosis (**F**,**G**). The OCT images were then analyzed by automated image processing and algorithm/artificial intelligence (**H**) based classification and compared with histological diagnosis.

This clinical study was conducted with the approval of the independent institutional ethics committee (Narayana Health Medical Ethics Committee (NHH/MEC-CL-2015-279), and KLE Society’s Institute of dental Sciences (KIDS/IEC/11-2015/8)) and monitored by the committee. The study included subjects who were more than 18 years old, consented for the investigation and clinically diagnosed with any oral lesions. Subjects with reduced mouth opening (less than 2 cm); those undergoing treatment for tuberculosis, HIV, HBV, HCV, and oral cancer; and pregnant women were excluded. The study participants were recruited from two different settings; tertiary care setting from the Department of Head and Neck Oncology, Mazumdar Shaw Medical Center, Narayana Health City, the Department of Oral Medicine, KLE Institute of Dental Sciences and from community settings (oral cancer screening camps conducted across Bangalore) during the time-period from December 2015 to November 2016. Subjects underwent incision/punch biopsy from the same lesion site, wherein the OCT images were taken and were administered standard-of-care treatment according to histology diagnosis (Figure 1). For the OCT imaging, the device was placed on the mucosal lesion for 30–60 s at 90 degrees’ angulation and the images were captured. Imaging was followed by incisional or excisional biopsy (wherever indicated) within a period of 10–15 min. The captured images were classified by simple algorithm developed previously [23,24]. The image features were extracted using multiple (*n* = 14) artificial neural networks (ANN) and the support vector machine (SVM) model was developed. Both the methods were compared with histological or clinical diagnosis depending on whether biopsy was indicated or not.

### 2.2. OCT System Design

Optical coherence tomography can generate high-resolution cross-sectional images of the oral mucosa (Figure 1E) [23,24]. OCT techniques can generate tomographic images by utilizing the differences in refractive index due to cells, organelles, and fibers in extracellular matrix found in biological tissues. The device used in this study is a spectral-domain OCT (SD-OCT) system consisting of a 2D scanning long GRID rod probe and uses a low coherence light (Figure 1B). The SD-OCT system used a center wavelength of 930 nm. It has an axial resolution of 7.0 μm and a lateral resolution of 15.0 μm with an oral mucosal penetration depth of 0.2–1 mm. Using a 20 kHz, 1024-point CCD line-scan camera on the spectrometer detection arm, an imaging speed of 1.2 kHz was achieved (2 images per second). In the hand-held imaging scanner, a GRIN rod relays the light from the proximal end of the probe to the patient’s oral mucosa.

### 2.3. Analysis of OCT Images

The automated interpretation of OCT images was implemented by two approaches; a MATLAB based simple algorithm-score [23,24] and an Artificial Neural Network-Support Vector Machine (ANN-SVM) based model. Multiple images (range of 10–15) were captured from oral mucosal lesions. The raw images were evaluated in two ways; by a trained oral physician (in order to remove blank, blurry, and poor images) and by using a non-reference image quality evaluator, Naturalness Image Quality Evaluator (NIQE) [25]. Images with high noise, artifacts and low quality (based on physician input) were removed from the image data set (Figure S1A). The significance of stationarity of the data quality change as assessed by NIQE was evaluated using Augmented Dickey Fuller test (ADF) [26] and Kwiatkowski–Phillips–Schmidt–Shin (KPSS) test [27]. NIQE score showed an increasing variation in the image quality (Figure S1A), with image quality worsening with time (ADF test, *p* = 0.10 and KPSS test, *p* = 0.01). However, assessment of the quality across different diagnostic categories as well as study-sites did not show any trend (Figure S1B) [26,27]. These images were taken forward for the analysis with their histopathological or clinical diagnosis (wherever biopsy was not indicated) as the gold standard.

#### 2.3.1. Algorithm-Score Based Analysis

The simple algorithm-score was derived from intensity measurements and thickness of the epithelium and sub-epithelium in the OCT images [23]. Firstly, a region of interest (ROI) was selected after image enhancement. An edge detection algorithm was applied on the ROI to obtain the edge of the first layer and OCT score was derived. The OCT score was used to stratify the images as malignant (OCT score range: −0.0580 to −0.0780), dysplastic (Range: −0.0780 to −0.0918) or normal (Range: −0.0918 to −0.1280) as described previously [23,24]. The efficacy of the algorithm was ascertained by using histological diagnosis as the reference standard.

#### 2.3.2. ANN-SVM Based Analysis

**Figure 2:**
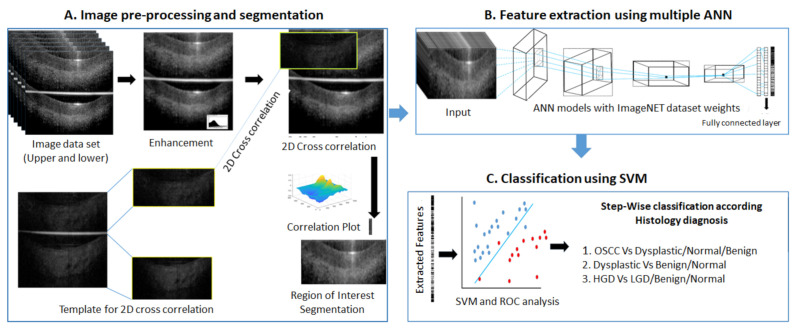
ANN-SVM based image analysis pipeline. Image data sets selected for image pre-processing and segmentation (**A**) after quality evaluation. The image was enhanced by Gaussian filter. Template images were used to segment region of interest of upper and lower sections by 2D-cross correlation. Feature extraction (**B**) was performed on segmented images by multiple artificial neural networks (ANN). The extracted features were used for developing Support Vector Machine model (SVM) model for each ANN feature vectors (**C**). The receiver operating characteristic (ROC) curve analysis was performed in cross-validation data set to find out optimal cut-off score for classification. The SVM-model validated in test data set and classified according cut-off score. The ANN-SVM models were developed for stepwise classification- initially for classifying OSCC from dysplastic/normal/benign lesions and then dysplastic from benign/normal lesions. OSCC: Oral squamous cell carcinoma, HGD: High grade dysplasia–Moderate/Severe dysplasia, LGD: Low grade dysplasia- Mild dysplasia, hyperplasia, ANN: Artificial neural network, 2D- 2 dimensional, SVM: Support vector machine, ROC: Receiver Operating Characteristic.

The image analysis pipeline included image preprocessing, feature extraction using multiple deep neural networks, and Support Vector Machine (SVM) model (Figure 2) development. The training was performed in two steps, delineating the lesions according to the risk of malignancy and grades of dysplasia. The models were developed for sequential separation of oral malignant lesions from the dysplastic and non-dysplastic lesions and followed by the differentiation of dysplastic from non-dysplastic oral lesions. All the image preprocessing, feature extraction, SVM model development, and validation were performed using MATLAB 2019a version.

##### Image Data Preparation

Datasets from histologically or clinically (normal and benign lesions wherein biopsy was not indicated) annotated OCT images were classified into training and test sets (Table S1). The training set included images from our previous studies [23,24] and the current study (Cases: 127; Images: 3594). In the training set, 40% of the images were from previous study dataset and 60% from the current, prospective study cohort. The testing of ANN-SVM model was performed in 271 oral sub-sites (stand-alone dataset of 2129 images) (Table S1).

##### Image Preprocessing

From each acquired OCT image, a pair of images was generated, consisting of the upper original image as well as a lower mirror image separated by a line (Figure 2A). The images were normalized and smoothened by Gaussian filter. A high-quality image set (upper and lower) was used as a template to segment the region of interest (ROI) from each of the images using a normalized 2D cross correlation [28,29]. A normalized 2D cross-correlation method segmented the images into upper and lower images and filtered out poor quality images (Figure 2A). The upper and lower segmented images were used for training.

##### Feature Extraction by Deep Learning and SVM Model

The images from the training data set (*n* = 3594; included upper/lower mirror images) were further randomly divided for SVM model training (70%) and cross-validation (30%). Feature extractions were performed using multiple, simple to complex neural networks (*n* = 14) (Table S2). The images were passed through all the layers of each neural network till the fully connected layer (before softmax layer) [29] without back propagation (keeping weights of ImageNet dataset). The features were then extracted from this layer using activation function (MATLAB 2019a) and SVM model was generated (fitcecoc function, learner–Linear) using the training set (70%) for binary classification. The model was then validated in the cross-validation dataset (30%) and the score was calculated by taking the average prediction score of all images of the same site. The prediction score for the validation set was analyzed and optimal threshold cut-off score established for every ANN-SVM model using receiver operating characteristic (ROC) curve analysis (Figure 2C). The image features were extracted from test data using each neural network and classified using the trained SVM model. The final diagnosis of the subject was arrived at according to the cut-off calculated from ROC curve analysis of the cross-validation data set. The results of each neural network model were evaluated for their sensitivity, specificity, and accuracy in distinguishing malignant and dysplastic images sequentially.

### 2.4. Statistical Analysis

The minimum sample size required (diagnostic test), to validate the device and algorithm were calculated. Considering alpha value of <0.05 and power of 80% the minimum patients required for the study was 207 to show a sensitivity greater than 91% (Null hypothesis: sensitivity = 85%; Alternate hypothesis: sensitivity ≠ 85% or sensitivity = 91%) [23,24,28]. We included a possible drop out of 20% due to poor image quality and estimated a sample size of 249 for the study. Additionally, from healthy volunteers (*n* = 25), an average of five oral sub-sites were captured.

Descriptive statistics were used to summarize details of patient demography, clinical features and pathology diagnosis. The distribution of the OCT score across normal/benign, dysplastic and OSCC subjects was determined. Kruskal-Wallis test was used to determine the statistical significance between multiple groups. The sensitivity, specificity, and accuracy were calculated. ROC curve analysis was performed to find cut-off score. All statistical analyses were carried out using the software MedCalc v14.8.1 and image analysis were performed by MATLAB 2019a. The graphs required for the manuscript were prepared using MedCalc v14.8.1 and Tableau 2019.4.8.

## 3. Results

### 3.1. Clinical and Demographic Details of Patients

The subjects were recruited as per the inclusion and exclusion criteria from a tertiary cancer center (MSMC: 51.1%), dental oral medicine clinic (KLES: 28.1%), and community screening centers (20.9%) (Figure S2A). Majority of the subjects from community screening (100%) and tertiary cancer center (73.6%) were males (Figure S2B), while the age of patients in the dental clinic and tertiary cancer center followed the same distribution (median age > 45 years; Figure S2C). In the community, the median age of recruited subjects was 32 years. The major sites captured were buccal mucosa (53.9%) and labial mucosa (12.1%) (Figure S2D). The majority of cases recruited from dental clinic and community centers were dysplastic (67.9%), while most of the malignant cases (86.7%) were enrolled from the tertiary cancer center (Figure S2E). Eighty percent of subjects had a habit history of tobacco chewing, smoking, or both (Figure S2F), with tobacco chewers being the majority of the population.

**Figure 3:**
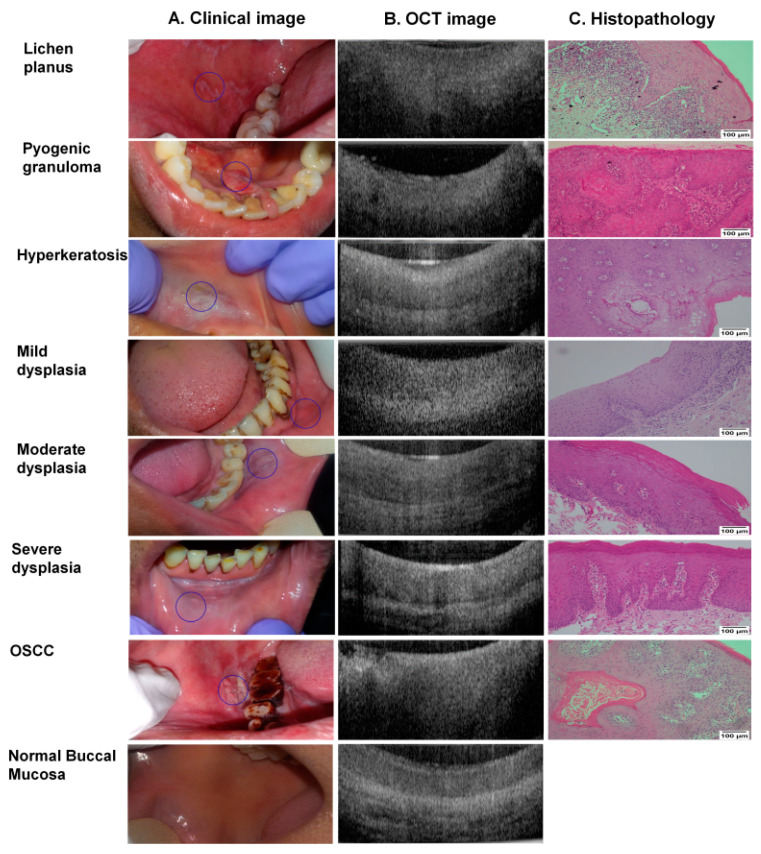
Clinical, OCT & histology images. Clinical (**A**) and OCT (**B**) images were captured for all the subjects and the biopsy tissues collected (wherever indicated) were assessed by histopathology (**C**). Histology images were taken at 100× resolution (scale bar = 100 µm) using Nikon DSFi2 and NIS elements D4 20.0. The non-dysplastic lesions shown were histologically diagnosed with lichen planus, pyogenic granuloma, and hyperkeratosis. Normal buccal mucosa images were taken from healthy volunteer without any habit history. Representative images of all dysplastic grades and a buccal oral squamous cell carcinoma (OSCC) are also depicted.

Each subject recruited into the study underwent oral examination and subsequently, OCT images were captured (Figure 1 and Figure 3). A total of 381 sub-sites images were captured from 249 subjects (subjects had multiple sites, especially from normal healthy participants); 347 sub-sites were used for analysis (17 subjects were excluded due to lack of biopsy) (Figure S3).

### 3.2. Algorithm Prediction Score Correlates with Histopathology Diagnosis

**Figure 4:**
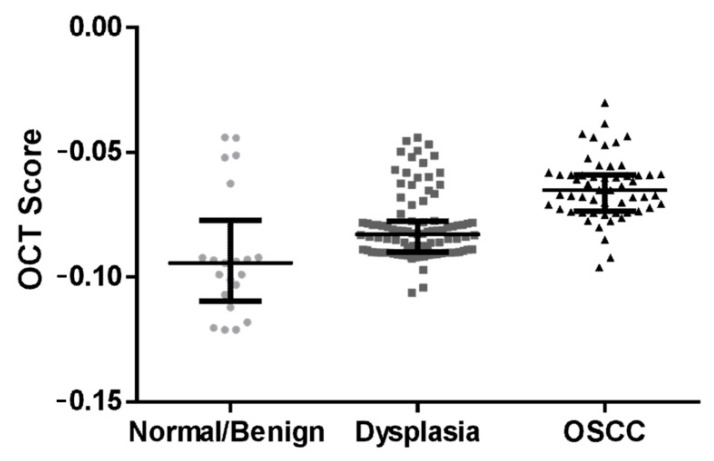
OCT algorithm prediction score distribution between the different patient cohorts. Box and whisker plot depicting the algorithm scores of oral squamous cell carcinoma (OSCC), dysplasia and normal/benign lesions. The score significantly increases (*p* < 0.005) as the disease progresses. Graph shows median and inter-quartile range.

A total of 172 oral sub-sites were prospectively analyzed by the algorithm, considering parameters of intensity and epithelial thickness. The OCT scores obtained were compared with the histological diagnosis. Comparative analysis of the sub-sites indicated that the algorithm-score of dysplastic lesions (−0.0828 ± 0.0121) was significantly different from that of the OSCC lesions (−0.0651 ± 0.0142, *p* < 0.005) and the normal/benign sub-sites (−0.0943 ± 0.015, *p* < 0.005) (Figure 4).

As a next step, the differences in scores were compared across the dysplastic and OSCC patients based on their age, gender, tobacco usage, and lesion-site (Figure S4). Among these parameters, significant differences were observed based on the tobacco chewing risk habit (*p* = 0.035) and age (*p* = 0.045) (Figure S4). Dysplastic patients with tobacco chewing had a lower median score (−0.0838) as compared to patients with alcohol/smoking/without habits (−0.0782). Similarly, patients grouped based on age showed a difference in OCT score (age ≤ 45: −0.0778, age <= 45: −0.0838; *p* = 0.045), indicating the effect of these parameters on the OCT image. There was, however, no difference observed in the scores when the patients were categorized based on site or gender.

A comparison of the OCT scores of mild (median score: −0.0832), moderate (median score: −0.0828), and severe dysplasia (median score: −0.0835) (Figure S4E) showed no significant difference (*p* = 0.0921) indicating that the OCT score was unable to differentiate the grades of dysplasia. Nevertheless, the algorithm score could delineate OSCC from others (dysplasia/normal/benign) with a sensitivity of 93% (CI: 82–98%) and specificity of 74% (CI: 65–82%) respectively (Table 1). Similarly, dysplasia could be delineated from normal/benign with a sensitivity of 95% (CI: 88–98%) and specificity of 76% (CI: 52–91%).

### 3.3. ANN-SVM Model Delineated Grades of Dysplasia

**Figure 5:**
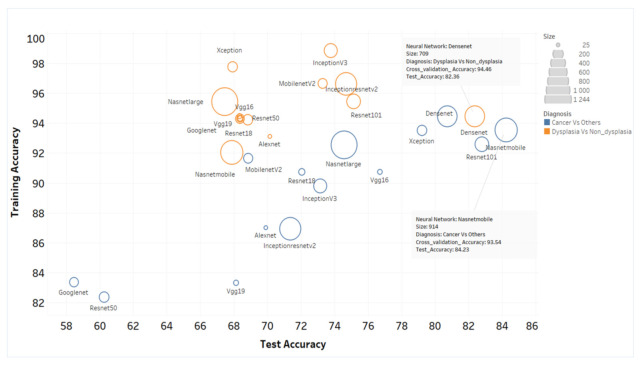
Accuracy of the various ANN-SVM models in delineating the patient cohorts. The training and test accuracy of the 14 neural networks used in the study in delineating cancer from dysplasia/non-dysplastic lesion and dysplasia from non-dysplastic lesions were depicted. The size of circle represents the size of neural network. The less overfitting models were NASNetMobile and DenseNet-201.

**Figure 6:**
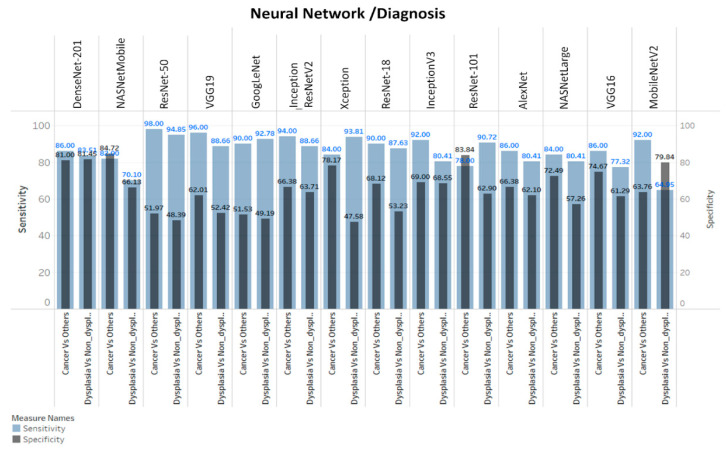
Sensitivity and specificity of neural networks. DenseNet-201 and NASNetMobile showed best sensitivity/specificity in delineating cancer vs others whereas DenseNet-201 was best in differentiating dysplasia vs non-dysplasia lesions.

All the images were passed through layers of 14 pre-trained ANN architecture, features were extracted from fully connected layers of ANN and the SVM model was developed using these features. The optimum threshold score values of SVM-model (Figures S5–S7), calculated by ROC curve analysis from the cross-validation data set (30% of the training set), showed 82–94% training accuracy and 60–85% test accuracy in delineating cancer from other lesions (Figure 5, Table S3). NASNetMobile and DenseNet-201 feature extraction showed the highest accuracy in delineating oral cancer and dysplastic lesions. DenseNet-201-SVM model showed a sensitivity and specificity of 86% and 81% respectively in delineating OSCC from others (Table 1, Figure 6).

The models showed 92–98% accuracy in the training set and 62–84% accuracy in the test sets for the detection of dysplastic lesions (Figure 5). DenseNet-201-SVM model showed highest test sensitivity (Table 1; 84%). Furthermore, Inception-ResNet-v2-SVM model showed high sensitivity (83%; CI: 72.2–90.4) in delineation of high-grade dysplastic lesions (HGD; moderate/severe dysplasia) from low-grade lesions (mild-dysplasia/benign/normal).

### 3.4. Clinical Application of OCT Device in Triaging Patients

**Figure 7:**
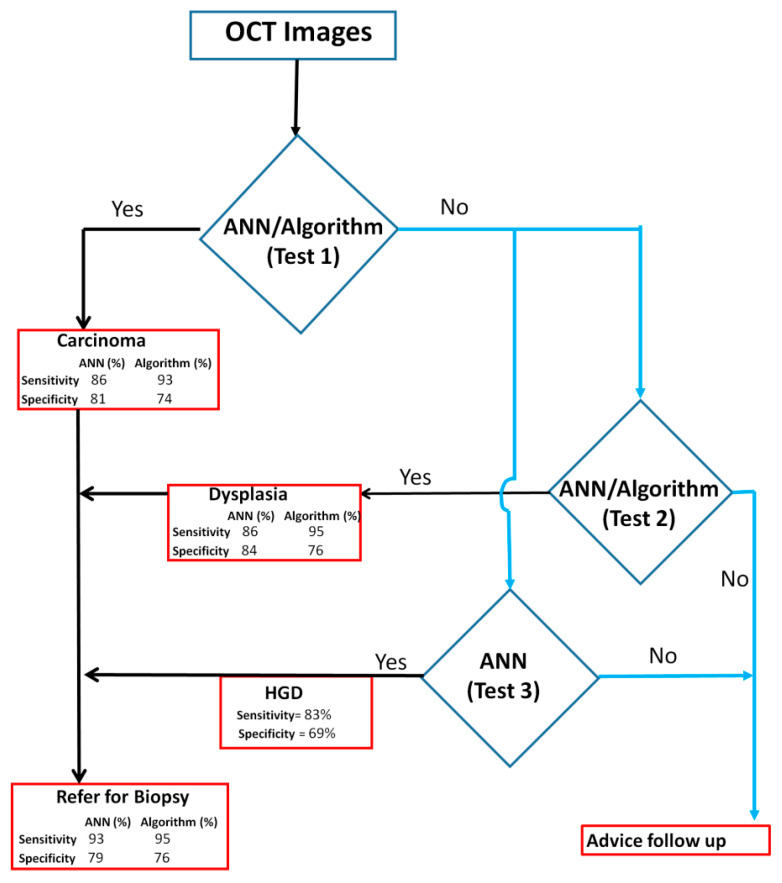
Decision tree for incisional biopsy from OCT diagnostic system. The decision tree consists of Test 1, which will identify OSCC patients. Test 2 and Test 3 can be used to identify patients with dysplasia and high-grade dysplasia (HGD) respectively. The combined sensitivity of these three tests ranges between 93–95% for delineating oral cancer and dysplasia. This decision tree can be used to triage high-risk patients in oral cancer screening camps. ANN: Artificial neural network.

A biopsy-decision model (Figure 7) was developed according to the automated image analysis based on either the algorithm score or the ANN-SVM based diagnosis. The model consists of multiple steps to triage the patients. As a first step, automated algorithm score and/or DenseNet-201-SVM delineated OSCC from dysplastic/non-dysplastic lesions (sensitivity: 86% (ANN) and 93% (algorithm) and specificity: 81% (ANN) and 74% (algorithm)), and the OSCC cohort can be referred for biopsy. The patients negative for test-1 can be assessed for the presence of dysplasia (Sensitivity: 86% (ANN) and 95% (algorithm)) (Figure 7). As a final step, the dysplastic patients could be further categorized into high-grade dysplasia using Inception-ResNet-v2-SVM (Sensitivity: 83%) (Figures S7–S9, Table S3). This final model provides a sensitivity of 96% (CI: 86.3–99.5) and 92% (CI: 84.4–96.4) for delineating OSCC and dysplastic lesion respectively. It also showed specificity of 79% (ANN) and 76% (algorithm) for delineating cancer/dysplastic lesions from non-dysplastic lesions (Figure 7).

## 4. Discussion

Early detection and regular surveillance of suspicious oral lesions are critical for decreasing mortality rate of OSCC. In this study, we validated a portable, low-cost OCT-based screening device for its accuracy in identifying malignant and potentially malignant lesions in resource-constrained settings. The simple algorithm score was able to delineate cancer from others with a sensitivity and specificity of 93% (CI: 82–98) and 74% (CI: 65–82) respectively. Besides, the combination of OCT imaging with deep learning could differentiate high-grade OPML with a sensitivity of 83%. This is the first large cohort study with varied patient groups (benign and different grades of dysplasia), deploying OCT-based imaging integrated with automated diagnosis (both simple algorithm and ANN-SVM model). The results suggested that OCT-based identification of malignant and dysplastic lesions are equivalent to incision biopsy-based diagnosis.

Previous studies in oral cancer using OCT imaging were mainly carried out ex vivo samples with the images being interpreted by trained observers. The diagnosis, being based on the manual calculation of the epithelium thickness, had certain subjectivity introduced by inter-observer variation. Nevertheless, OCT imaging was capable of delineating oral cancer with a sensitivity and specificity of 85–92% and 78–94% in these studies [18,20]. Fluorescence lifetime imaging in combination with OCT in the hamster cheek pouch model discriminated benign, dysplastic, and cancerous lesions [30] with a sensitivity and specificity of 81–90% and 92–96%. However, the major limitation of the study was that a majority of the benign lesions were hyperplastic or hyperkeratotic cases of the buccal mucosa. In comparison to these studies, in our study, imaging was done in vivo, in the patients, including all sites of the oral cavity, and subjectivity in diagnosis was addressed by employing automated image analysis and interpretation. This integration of OCT imaging with algorithm-score resulted in a sensitivity of 93% (CI: 82–98) and 95% (CI: 88–98) in delineating OSCC and dysplasia respectively (Table 1). Additionally, documentation of OCT images of healthy volunteers indicated site-specific variation; the dorsal surface of the tongue showed a different architecture displaying a high variation in the site-based optical properties compared to the other four oral sub-sites (Figure S10A). The floor of the mouth consistently showed the thinnest epithelium component, whereas the buccal mucosal lining showed maximum thickness compared to the other surfaces, as previously reported [19].

Automated image interpretation is a necessary tool that is to be applied in screening settings as it nullifies subjectivity in diagnosis, and enables efficient and periodic follow up of patients. A recent study integrated ANN with a three-dimensional OCT imaging system to differentiate normal and abnormal head and neck mucosa, wherein a pre-trained ANN (AlexNet) analyzed six malignant, one dysplastic as well as their corresponding tumor margin tissues, obtained a sensitivity and specificity of 100% and 70% respectively in identifying cancer from others [31]. In our study, both automated diagnostic platforms (ANN and the algorithm-based score) were able to identify malignant/dysplastic lesions with a sensitivity of 93–96% and specificity of 74–79% (Figure 7). In the ANN-based analysis we explored 14 pre-trained neural networks for feature extraction and SVM model was developed. Some of these networks are quite complex containing 25–1244 layers and our study showed the best results with DenseNet-201 and NASNetMobile (Figure 6). The image analysis and ANN based simple feature extraction method, uses low computational power, less training, and validation time. However, the ANN-SVM model was overfitting due to smaller training dataset and increased complexity. Overall, automation in this study was significant in improving efficacy and reducing the subjectivity of OCT-based diagnosis of oral malignant/premalignant lesions.

The prototype device combined with automated diagnosis in our study gives a sensitivity of 96% (CI: 86.3–99.5) and 92% (CI: 84.4–96.4) for delineating malignant and dysplastic lesions which strengthens the application of OCT as point-of-care device in a low resource setting (Figure 7). Additionally, the ANN-based analysis was able to delineate high-grade dysplastic lesions with a sensitivity of 83%, which is very beneficial in the management of high-risk lesions in follow-up visits. This process (Figure 7) could be used in low resource settings in real time, wherein, the captured images will be processed and fed to the ANN-SVM model to generate the diagnosis using a graphic user interface. Our recent study, wherein oral cytology was combined with ANN, could detect high-grade dysplastic lesions with a comparatively lower sensitivity of 73%, owing to the limitations of the cytology technique [32]. A recent clinical trial (NCT02415881) in head and neck squamous cell carcinoma used an anti-epidermal growth factor receptor contrast agent (panitumumab-IRDye800CW) and showed that the signal to background ratio was significantly higher in high-grade dysplasia as compared to low grade dysplasia/normal (*p* < 0.05) [33]. Another study, in oral leukoplakia, based on nuclear morphometric analysis (brush biopsy) revealed that low-grade and high-grade dysplasia can be significantly differentiated based on DNA content, DNA index, nuclear area, nuclear radius, nuclear intensity, sphericity, and fractional dimension (*p* < 0.01) [34]. Although these methods showed significant results in identifying high-grade dysplasia, they are either minimally invasive procedures or need extensive processing/specialist expertise. The ability to accurately distinguish high-grade lesions from mild dysplasia, benign and normal, using non-invasive OCT imaging, is one of the most significant aspects of this study.

The prototype device is a low-cost system, constructed at 10% of the cost of existing commercial system [23,24] and this study further established its applicability in low-resource settings. The system is apt for clinical use, both in screening and surveillance settings due to multiple advantages; the speed of image capture (two images per second), safety (easily sterilized, harmless radiation), ease of use (the device is user-friendly, can be operated by a nurses/dentists), rapid detection (OCT can enable real-time evaluation), and monitoring (enables repeated imaging for surveillance). However, there were a few challenges, one of them being the ability to access different oral cavity regions. A redesign with specific angulation at the tip can probably enable access to difficult sites such as the retromolar-trigone (RMT). Another challenge in our study is a comparatively low specificity (74–79%), which can be attributed to two main issues; intensity difference in benign lesions and dorsal tongue lesions. The intensity difference in OCT image is attributed to differential presence of chromophores (melanin, hemoglobin) in the epithelium, basal membrane, and lamina propria [21]. Benign lesions lacking epithelium (aphthous ulcer, traumatic ulcer, oral pemphigus) are hence misdiagnosed as malignant. Secondly, the architecture of dorsal tongue tissue, consisting of stratified squamous keratinized epithelium and characterized by papillae, is known to introduce shadowing artifacts influencing the image quality (Figure S10B) [35]. In our ANN-based analysis, 7.7% (*n* = 21) of the lesions were from the dorsal tongue, out of which 12 (57.1%) were misclassified as cancer (cancer: 4 vs. others: 17) and 3 (17.6%) as dysplasia (dysplasia: 3 vs. others: 14), thereby accounting for high false positivity. Another factor to be considered is that, the spatial resolution of our OCT system is poor considering the advanced OCT devices available currently in the market with resolutions of 1 µm [36]. The current resolution although not ideal, was sufficient for the aim of the current study, the device being low cost, to be utilized in low resource settings to delineate malignant or OPML lesions from benign or normal. Notwithstanding these challenges, the system showed high sensitivity, a necessity for any screening/early detection device. The OCT device, integrated with ANN/algorithm-based diagnosis, improved decision making in field settings or dental clinics in triaging the patients with malignant and high-grade dysplastic lesions to the tertiary cancer center, thereby enabling early detection, accurate referrals, and timely/appropriate treatment.

## 5. Conclusions

This study has validated the efficacy of the robust, low-cost OCT device as a point-of-care device in differentiating malignancy, dysplasia, and normal/benign oral lesion in resource-constrained settings. The screening and surveillance pipeline will include, imaging the lesion, feeding them to the decision-tree model for data interpretation, and accordingly a referral to the tertiary center for a biopsy if the patient is detected with a malignancy and high-grade dysplasia. The high accuracy, easy to use detection pipeline, good concordance with histology, and its portability suggests potential clinical application in screening and surveillance of oral cancer.

## Figures and Tables

**Table 1:** Sensitivity and specificity of simple algorithm and ANN. OSCC: Oral Squamous Cell Carcinoma, HGD: High Grade Dysplasia, LGD: Low Grade Dysplasia, TP: True positive, TN: True negative, FN: False negative, FP: False positive.

Method	Diagnosis	Sensitivity (TP/(TP + FN))	Specificity (TN/(TN + FP))	PPV	NPV
Algorithm-Score	OSCC Vs Dysplasia/Benign/Normal	93(51/55)	74(87/117)	63	96
	Dysplasia VsBenign/Normal	95(91/96)	76(16/21)	95	76
DensNet-201-SVM	OSCC Vs Dysplasia/Benign/Normal	86(43/50)	81(179/221)	51	96
	Dysplasia Vs Benign/Normal	84(81/97)	82(101/124)	78	86
Inception-ResNet-v2-SVM	HGD Vs LGD/Benign/Normal	83(63/75)	69(100/146)	58	89

## Data Availability

All data is included in the manuscript and the supplementary files. De-identified image data will be available upon request.

## Supplementary Materials

The following are available online at https://www.mdpi.com/article/10.3390/cancers13143583/s1. Figure S1: Distribution of NIQE (Naturalness Image Quality Evaluator) score. The graph (A) showed increase in NIQE scores (reducing the quality of the image) according to order of cases recruited. There is no pattern change according to diagnosis and study sites (B). SCC: squamous cell carcinoma, study sites: KLE, MSMC, Community Screening Site (CSS). Figure S2: Clinical and demographic details of subjects. The subjects were recruited from different study sub centers- tertiary cancer center, dental clinics and community screening camps (A). The detail distribution of age (B) and gender (C) of participants from each type of study centers were depicted. The majority of cases was presented with buccal mucosa lesions (D) and was dysplastic (E). The count and percentage of the various oral sub-sites (D) imaged, histology diagnosis (E) and risk-factor history (F) were depicted center-wise. Figure S3: Study consort chart. FigureS4: OCT algorithm score distribution across sub-sites, gender, age and habits. Sub-sites categorized into gingivo-buccal sulcus (GBS) and tongue (A) according to dysplasia and oral squamous cell carcinoma (SCC), showed no significant difference (*p* > 0.05). The same result (*p* > 0.05) was observed in gender-based distribution (B). Significant difference was observed in dysplastic cases when the age (C) or habits (D) were considered (*p* < 0.05). The scores did not show any significant difference between the grades of dysplasia (E) and between high-grade dysplasia (HGD)/low-grade dysplasia (LGD) (F). FigureS5: Receiver Operating Characteristic (ROC) curve analysis of 14 ANN-SVM model score for delineating oral squamous cell carcinoma from dysplasia/benign/normal. Figure S6: Receiver Operating Characteristic (ROC) curve analysis of 14 ANN-SVM model score for delineating dysplastic lesions from non-dysplastic lesions (Normal/Benign). Figure S7: Receiver Operating Characteristic (ROC) curve analysis of 14 ANN-SVM model score for delineating high grade dysplasia (moderate/severe dysplasia) from low grade dysplasia (mild dysplasia, benign, normal). Figure S8: Accuracy of 14 ANN-SVM model for delineation high grade dysplasia (moderate/severe dysplasia) from low grade dysplasia (mild dysplasia, benign, normal). Figure S9: Sensitivity and specificity of 14 ANN-SVM model in delineating high grade dysplasia (moderate/severe dysplasia) from low grade dysplasia (mild dysplasia, benign, normal). Figure S10: OCT images of different oral mucosa sub-sites. The architectural difference between sub-sites of oral mucosa (normal) is evident with buccal mucosa displaying the maximum epithelial thickness and floor of the mouth and gingival displaying the least (A). The dorsal tongue (B) showed architecture variation from the rest of the sub-sites. (B) OCT images of lesions/normal dorsal tongue. TableS1: Data set for artificial neural network (ANN) training and testing from current study and previous study dataset. TableS2: Artificial neural networks (*n* = 14) used for Feature Extraction and number of layers of each neural Network. Table S3: Test sensitivity and specificity of 14 ANN-SVM models.
