# Supplementary material for: Validation of a Point-of-Care Optical Coherence Tomography Device with Machine Learning Algorithm for Detection of Oral Potentially Malignant and Malignant Lesions
Source: Cancers (Basel). 2021 Jul 17;13(14):3583. doi: 10.3390/cancers13143583 (PMC8304149; doi:10.3390/cancers13143583)
Supplement: Supplementary file 1 [file cancers-13-03583-s001.zip › cancers-1212517-TableS1-S3.pdf]

cancers

Article

Validation of a Point-of-Care Optical Coherence Tomography Device with Machine Learning Algorithm for Detection of Oral Potentially Malignant and Malignant Lesions

Bonney Lee James[1,2,†], Sumsum P. Sunny[1,2,3,†], Andrew Emon Heidari[4], Ravindra D Ramanjinappa[1],Tracie Lam[4], Anne V. Tran[4], Sandeep Kankanala[5], Shiladitya Sil[5], Vidya Tiwari[6], Sanjana Patrick[6],Vijay Pillai[3], Vivek Shetty[3], Naveen Hedne[3], Darshat Shah[7], Nameeta Shah[7], Zhong-ping Chen[4], Uma Kandasarma[8], Shubhasini Attavar Raghavan[5], Subha Gurudath[5], Praveen Birur Nagraj[1,5,6], Petra Wilder-Smith[4], Amritha Suresh[1,3,*], and Moni Abraham Kuriakose[1,3,*]

1 Integrated Head and Neck Oncology Program (DSRG-5), Mazumdar Shaw Center for Translational Research (MSCTR), Mazumdar Shaw Medical Foundation, NH Health City, 560099 Bangalore, India; bonney.lee.james@ms-mf.org (B.L.J); sumsumsp@gmail.com (S.P.S.); ravindradr88@gmail.com (R.D.R.); praveen.birur@gmail.com (P.B.N.); amritha.suresh@ms-mf.org (A.S.); makuriakose@gmail.com (M.A.K.)

2 Manipal Academy of Higher Education (MAHE), Manipal, 576104 Karnataka, India

3 Department of Head and Neck Oncology, Mazumdar Shaw Medical Center, NH Health City, 560099 Bangalore, India;drvijaypillai@gmail.com (V.P.); vivek.shetty.dr@narayanahealth.org (V.S.); hednenaveen@gmail.com (N.H)

4 Beckman Laser Institute, UCI, Irvine, California 92612, USA.; aheidari.uci@gmail.com (A.E.H.); tracielam.m@gmail.com (T.L.); annevt@uci.edu (A.V.T.); z2chen@uci.edu (Z.C.); pwsmith@uci.edu (P.W.-S.)

5 Department of Oral Medicine and Radiology, KLE Society's Institute of Dental Sciences, 560022 Bangalore, India; kankanala.sandeep86@gmail.com (S.K.); shiladitya.sil@gmail.com (S.S.); subhashiniar@gmail.com (S.A.); drshubha.gurudath@gmail.com (S.G.)

6 Biocon Foundation, 560100 Bangalore, India; vidyatiwari96@gmail.com (V.T.); tanjupat@yahoo.com (S.P.)

7 Mazumdar Shaw Center for Translational Research (MSCTR), Mazumdar Shaw Medical Foundation, NH Health City, 560099 Bangalore, India; dshahms@hotmail.com (D.S.); nameeta.shah@ms-mf.org (N.S.)

8 Department of Oral and Maxillofacial Pathology, KLE Society's Institute of Dental Sciences, 560022 Bangalore, India; umak235@gmail.com

* Correspondence: amritha.suresh@gmail.com, amritha.suresh@ms-mf.org (A.S.); makuriakose@gmail.com (M.A.K.)

† Contributed Equally.

Citation: James, B.L.; Sunny, S.P.; Heidari, A.E.; Ramanjinappa, R.D.; Tran, A.V.; Kankanala, S.; Sil, S.; Tiwari, V.; Patrick, S.; Shetty, V.; et al. Validation of a Point-of-Care Optical Coherence Tomography Device with Machine Learning Algorithm for Detection of Oral Potentially Malignant and Malignant Lesions. *Cancers* **2021**, *13*, x. https://doi.org/10.3390/xxxxx

Academic Editor: Andreas Stadlbauer, Anke Meyer-Baese and Max Zimmermann

Received: date
Accepted: date
Published: date

Publisher's Note: MDPI stays neutral with regard to jurisdictional claims in published maps and institutional affiliations.

Copyright: © 2021 by the authors. Licensee MDPI, Basel, Switzerland. This article is an open access article distributed under the terms and conditions of the Creative Commons Attribution (CC BY) license (http://creativecommons.org/licenses/by/4.0/).

Table S1. Data set for artificial neural network (ANN) training and testing from current study and previous study data-set.

Cases Used for ANN Based Training and Validation							
**Histology**	Cancer		Dysplastic		Non-dysplastic		Total
**Merged-Studies**	Present study	Previous study[1]	Present study	Previous study[1]	Present study	Previous study[1]	
**Total cases (*n*)**	**85**		**147**		**167**		**399**
**Study wise(*n*)**	75	10	121	26	151	16	399
**Training set (*n*)**	25	10	24	26	27	16	128
**Test Set (*n*)**	50		97		124		271
**Training set (%)**	**41**		**34**		**26**		**32**
**Test set (%)**	**59**		**66**		**74**		**68**

Table 2. Artificial neural networks (*n* = 14) used for Feature Extraction and number of layers of each neural Network.

Neural Network Used	Layers
AlexNet	25
VGG16	41
VGG19	47
ResNet-18	72
GoogLeNet	144
MobileNetV2	155
Xception	171
ResNet-50	177
InceptionV3	316
ResNet-101	347
DenseNet-201	709
InceptionResnet-V2	825
NASNetMobile	914
NASNetLarge	1244

Table S3. Test sensitivity and specificity of 14 ANN-SVM models.

Diagnosis	Neural Network	Sensitivity	95% CI	Specificity	95% CI	Accuracy
**Cancer Vs Others**	**NASNetLarge**	84.5	70.9–92.8	72.5	66.2–78.2	74.5618
	**DenseNet-201**	86.2	74.62–93.85	81.4	74.7–85.55	81.936
	**InceptionResNet-v2**	94.4	83.5–98.7	66.4	59.9–72.5	71.3516
	**Xception**	84.3	70.9–92.8	78.2	72.2–83.3	79.2194
	**ResNet-101**	78.6	64.0–88.5	83.8	78.4–88.4	82.7888
	**NASNetMobile**	82.5	68.6–91.4	84.7	79.4–89.1	84.2304
	**VGG19**	96.6	86.3–99.5	62.0	55.4–68.3	68.1282
	**VGG16**	86.3	73.3–94.2	74.7	68.5–80.2	76.7094
	**MobileNetV2**	92.4	80.8–97.8	63.8	57.2–70.0	68.8432
	**ResNet-50**	98.5	89.4–99.9	52.0	45.3–58.6	60.2554
	**ResNet-18**	90.8	78.2–96.7	68.1	61.7–74.1	72.0584
	**GoogLeNet**	90.6	78.2–96.7	51.5	44.9–58.2	58.4546
	**AlexNet**	86.7	73.3–94.2	66.4	59.9–72.5	69.9116
	**InceptionV3**	92.4	80.8–97.8	69.0	62.6–74.9	73.14
**Dysplasia Vs Non-Dysplasia**	**NASNetLarge**	80.4	71.1–87.8	57.3	48.1–66.1	67.446
	**DenseNet-201**	83.5	74.6–90.3	81.5	73.5–87.9	82.3564
	**InceptionResNet-v2**	88.7	80.6–94.2	63.7	54.6–72.2	74.688
	**Xception**	93.8	87.0–97.7	47.6	38.5–56.7	67.9212
	**ResNet-101**	90.7	83.1–95.7	62.9	53.8–71.4	75.1408
	**NASNetMobile**	70.1	60.0–79.0	66.1	57.1–74.4	67.8768
	**VGG19**	88.7	80.6–94.2	52.4	43.3–61.5	68.3656

	**VGG16**	77.3	67.7–85.2	61.3	52.1–69.9	68.3432
	**MobileNetV2**	65.0	54.6–74.4	79.8	71.7–86.5	73.2884
	**ResNet-50**	94.9	88.4–98.3	48.4	39.3–57.5	68.8324
	**ResNet-18**	87.6	79.4–93.4	53.2	44.1–62.2	68.366
	**GoogLeNet**	92.8	85.7–97.0	49.2	40.1–58.3	68.3696
	**AlexNet**	80.4	71.1–87.8	62.1	52.9–70.7	70.1564
	**InceptionV3**	80.4	71.1–87.8	68.6	59.6–76.6	73.7684
**High Grade dysplasia Vs Low Grade/Benign/Normal**	**NASNetLarge**	57.3	45.4–68.7	76.0	68.3–82.7	69.6907
	**DenseNet-201**	94.7	86.9–98.5	35.6	27.9–44.0	55.63795
	**InceptionResNet-v2**	82.7	72.2–90.4	68.5	60.3–75.9	73.29702
	**Xception**	92.0	83.4–97.0	58.9	50.5–67.0	70.1209
	**ResNet-101**	94.7	86.9–98.5	49.3	41.0–57.7	64.69365
	**NASNetMobile**	80.0	69.2–88.4	61.6	53.2–69.6	67.86404
	**VGG19**	96.0	88.8–99.2	37.7	29.8–46.1	57.44387
	**VGG16**	76.0	64.7–85.1	63.7	55.3–71.5	67.8697
	**MobileNetV2**	94.7	86.9–98.5	30.8	23.5–39.0	52.46515
	**ResNet-50**	45.3	33.8–57.3	87.7	81.2–92.5	73.31674
	**ResNet-18**	73.3	61.9–82.9	73.3	65.3–80.3	73.30356
	**GoogLeNet**	77.3	66.2–86.2	61.6	53.2–69.6	66.95891
	**AlexNet**	64.0	52.1–74.8	76.0	68.3–82.7	71.95183
	**InceptionV3**	90.7	81.7–96.2	43.8	35.6–52.3	59.71537

1. Sunny, S.P.; Agarwal, S.; James, B.L.; Heidari, E.; Muralidharan, A.; Yadav, V.; Shetty, V.; Chen, Z.; Hedne, N.; et al. Intra-operative point-of-procedure delineation of oral cancer margins using optical coherence tomography. *Oral. Oncol.* **2019**, *92*, 12–19. doi: 10.1016/j.oraloncology.2019.03.006.
