# Supplementary material for: Validation of a Point-of-Care Optical Coherence Tomography Device with Machine Learning Algorithm for Detection of Oral Potentially Malignant and Malignant Lesions
Source: Cancers (Basel). 2021 Jul 17;13(14):3583. doi: 10.3390/cancers13143583 (PMC8304149; doi:10.3390/cancers13143583)
Supplement: Supplementary file 1 [file cancers-13-03583-s001.zip › Figure S10.pdf]

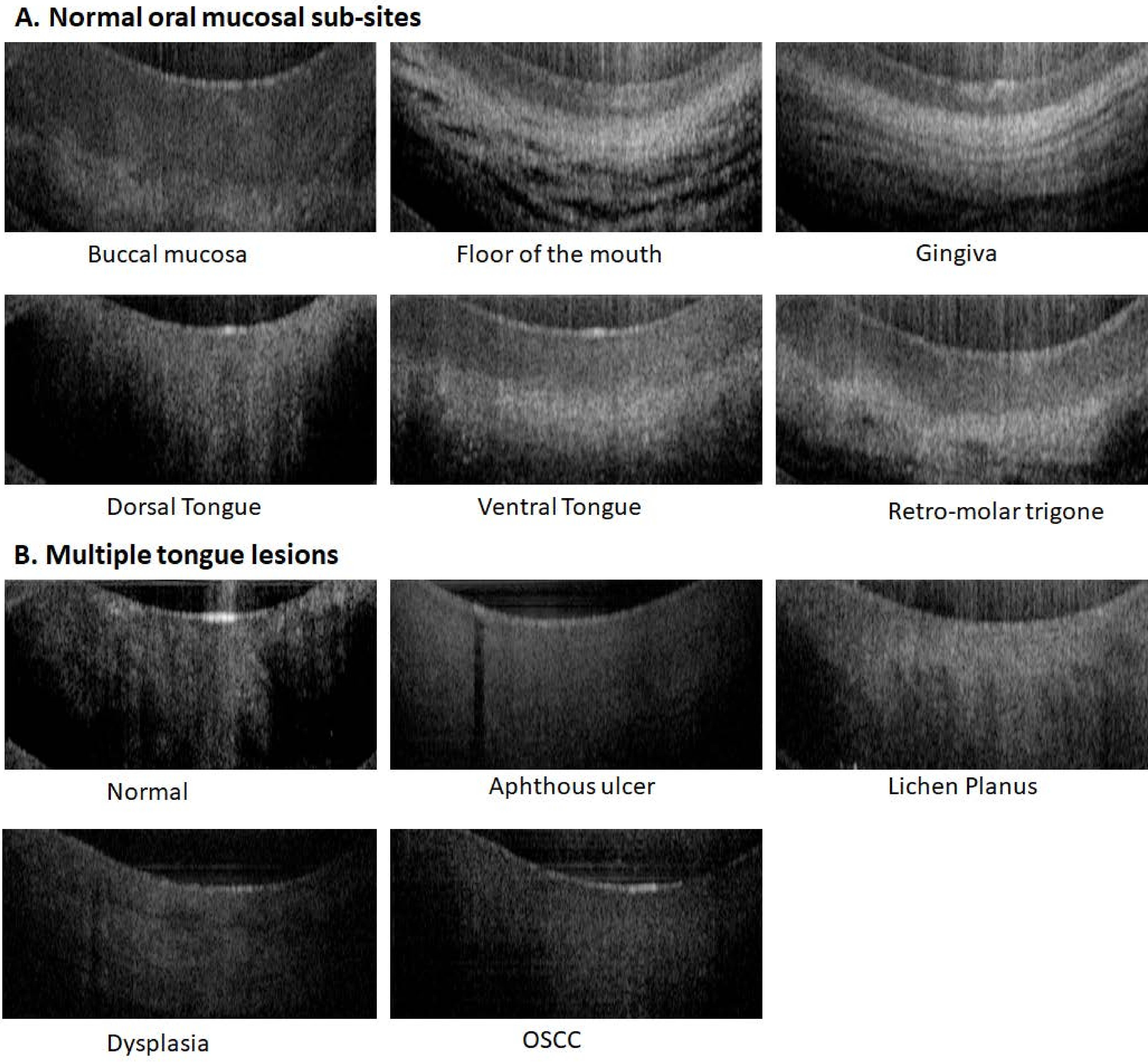
A. Normal oral mucosal sub-sites
Buccal mucosa
Floor of the mouth
Gingiva
Dorsal Tongue
Ventral Tongue
Retro-molar trigone
B. Multiple tongue lesions
Normal
Aphthous ulcer
Lichen Planus
Dysplasia
OSCC
