# Supplementary material for: Validation of a Point-of-Care Optical Coherence Tomography Device with Machine Learning Algorithm for Detection of Oral Potentially Malignant and Malignant Lesions
Source: Cancers (Basel). 2021 Jul 17;13(14):3583. doi: 10.3390/cancers13143583 (PMC8304149; doi:10.3390/cancers13143583)
Supplement: Supplementary file 1 [file cancers-13-03583-s001.zip › Figure S3.pdf]

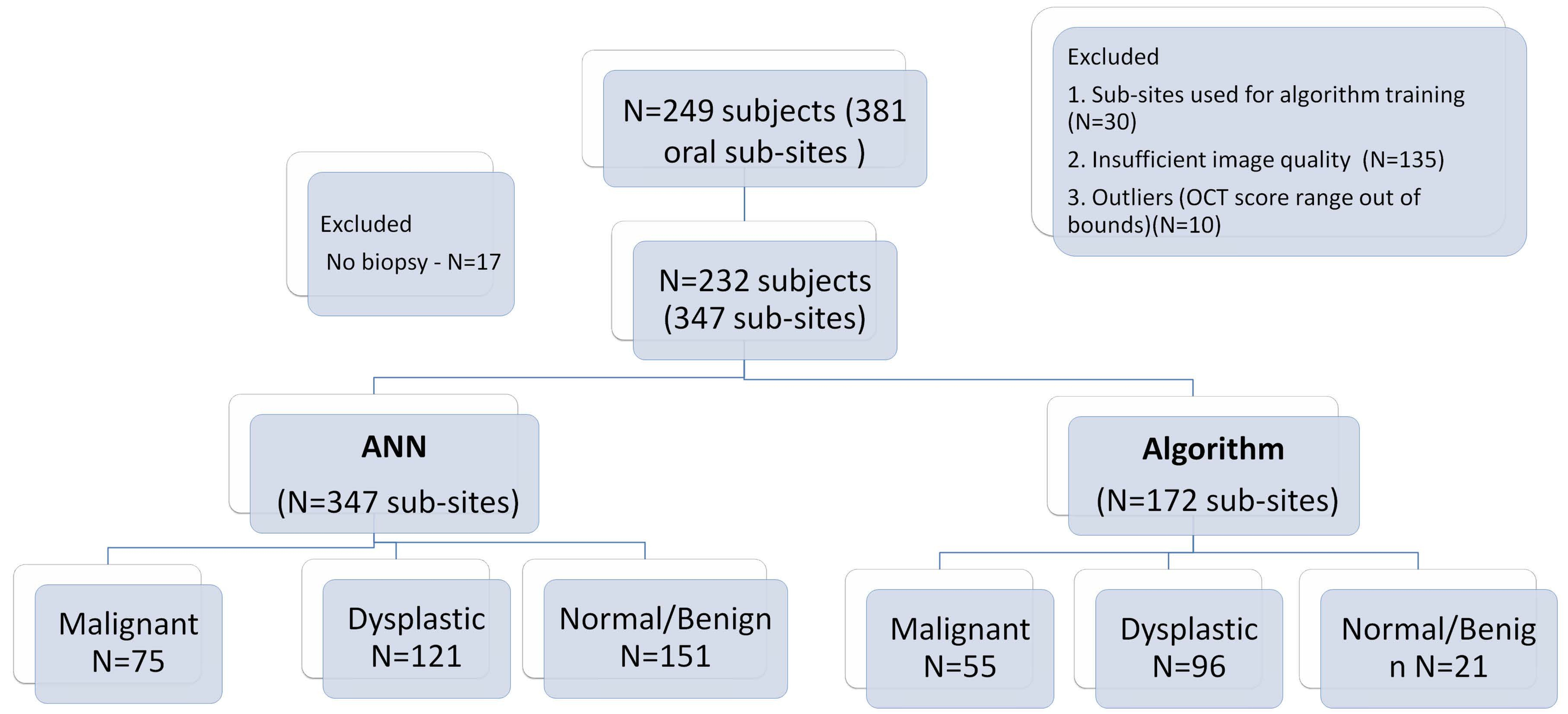
N=249 subjects (381 oral sub-sites)
Excluded
1. Sub-sites used for algorithm training (N=30)
2. Insufficient image quality (N=135)
3. Outliers (OCT score range out of bounds)(N=10)
Excluded
No biopsy - N=17
N=232 subjects (347 sub-sites)
ANN
(N=347 sub-sites)
Algorithm
(N=172 sub-sites)
Malignant N=75
Dysplastic N=121
Normal/Benign N=151
Malignant N=55
Dysplastic N=96
Normal/Benig n N=21
