# Supplementary material for: Validation of a Point-of-Care Optical Coherence Tomography Device with Machine Learning Algorithm for Detection of Oral Potentially Malignant and Malignant Lesions
Source: Cancers (Basel). 2021 Jul 17;13(14):3583. doi: 10.3390/cancers13143583 (PMC8304149; doi:10.3390/cancers13143583)
Supplement: Supplementary file 1 [file cancers-13-03583-s001.zip › Figure S5.pdf]

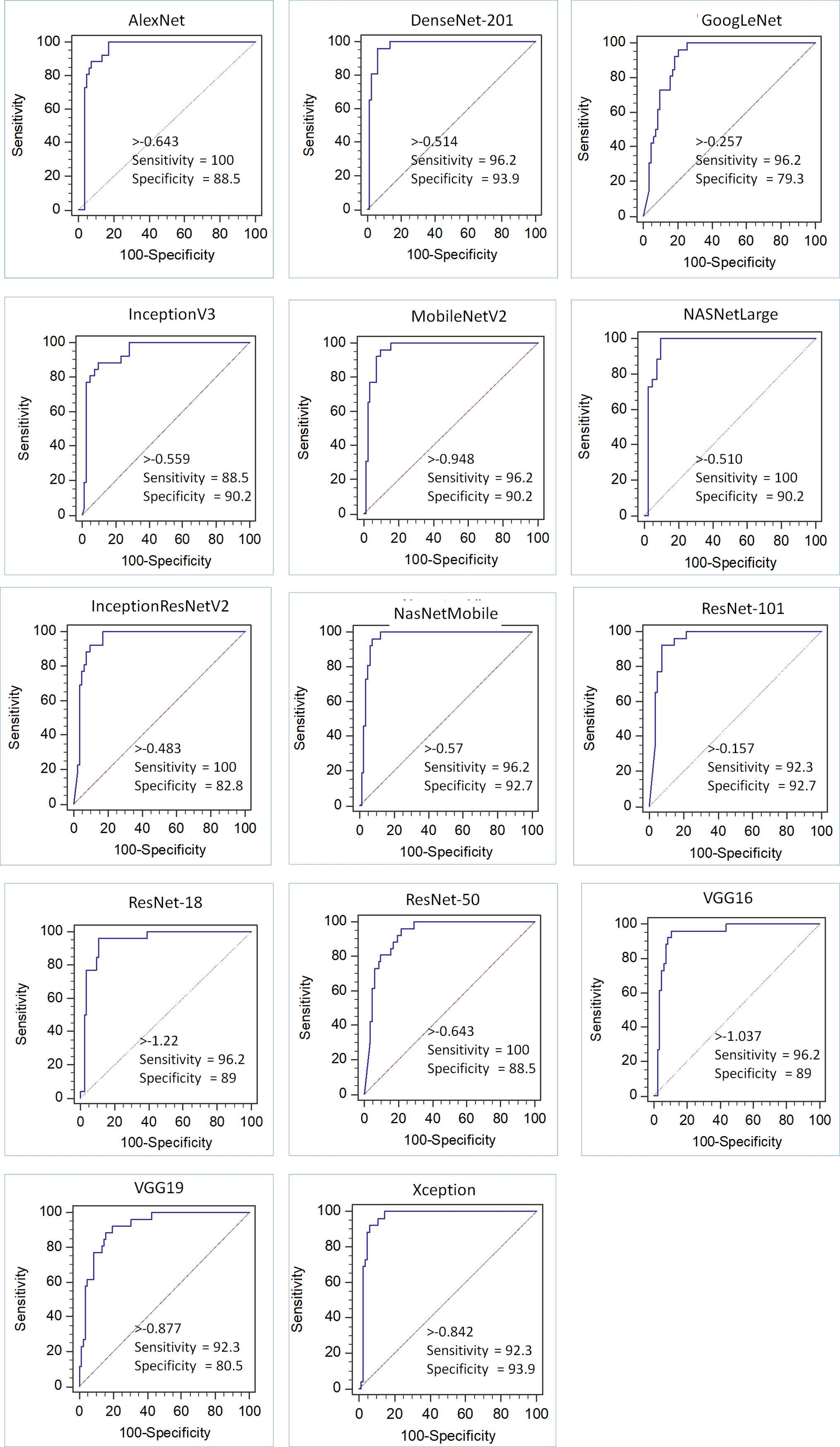

AlexNet
>-0.643
Sensitivity = 100
Specificity = 88.5
DenseNet-201
>-0.514
Sensitivity = 96.2
Specificity = 93.9
GoogLeNet
>-0.257
Sensitivity = 96.2
Specificity = 79.3
InceptionV3
>-0.559
Sensitivity = 88.5
Specificity = 90.2
MobileNetV2
>-0.948
Sensitivity = 96.2
Specificity = 90.2
NASNetLarge
>-0.510
Sensitivity = 100
Specificity = 90.2
InceptionResNetV2
>-0.483
Sensitivity = 100
Specificity = 82.8
NasNetMobile
>-0.57
Sensitivity = 96.2
Specificity = 92.7
ResNet-101
>-0.157
Sensitivity = 92.3
Specificity = 92.7
ResNet-18
>-1.22
Sensitivity = 96.2
Specificity = 89
ResNet-50
>-0.643
Sensitivity = 100
Specificity = 88.5
VGG16
>-1.037
Sensitivity = 96.2
Specificity = 89
VGG19
>-0.877
Sensitivity = 92.3
Specificity = 80.5
Xception
>-0.842
Sensitivity = 92.3
Specificity = 93.9
Sensitivity
100-Specificity
