# Supplementary figures and images for: Validation of a Point-of-Care Optical Coherence Tomography Device with Machine Learning Algorithm for Detection of Oral Potentially Malignant and Malignant Lesions
Source: Cancers (Basel). 2021 Jul 17;13(14):3583. doi: 10.3390/cancers13143583 (PMC8304149; doi:10.3390/cancers13143583)

A

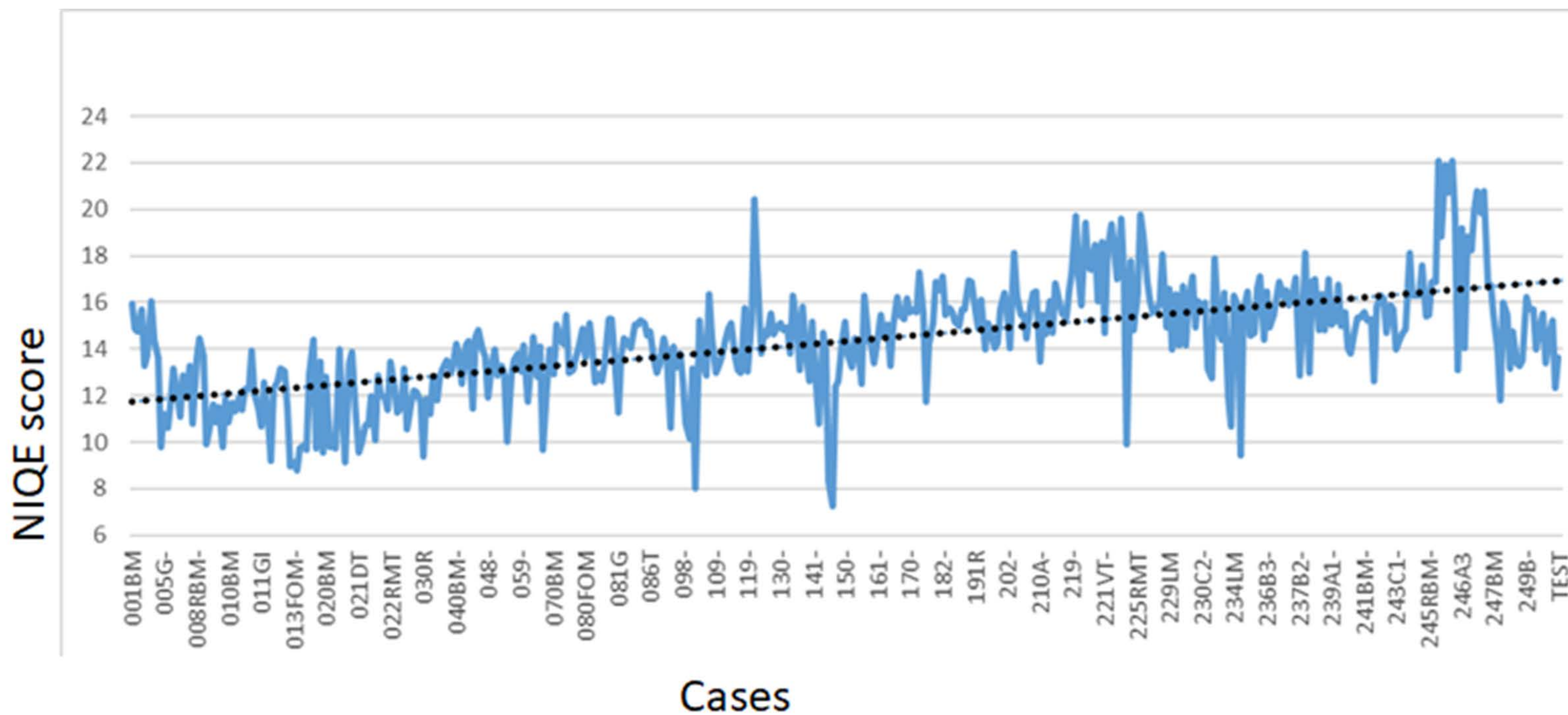

B

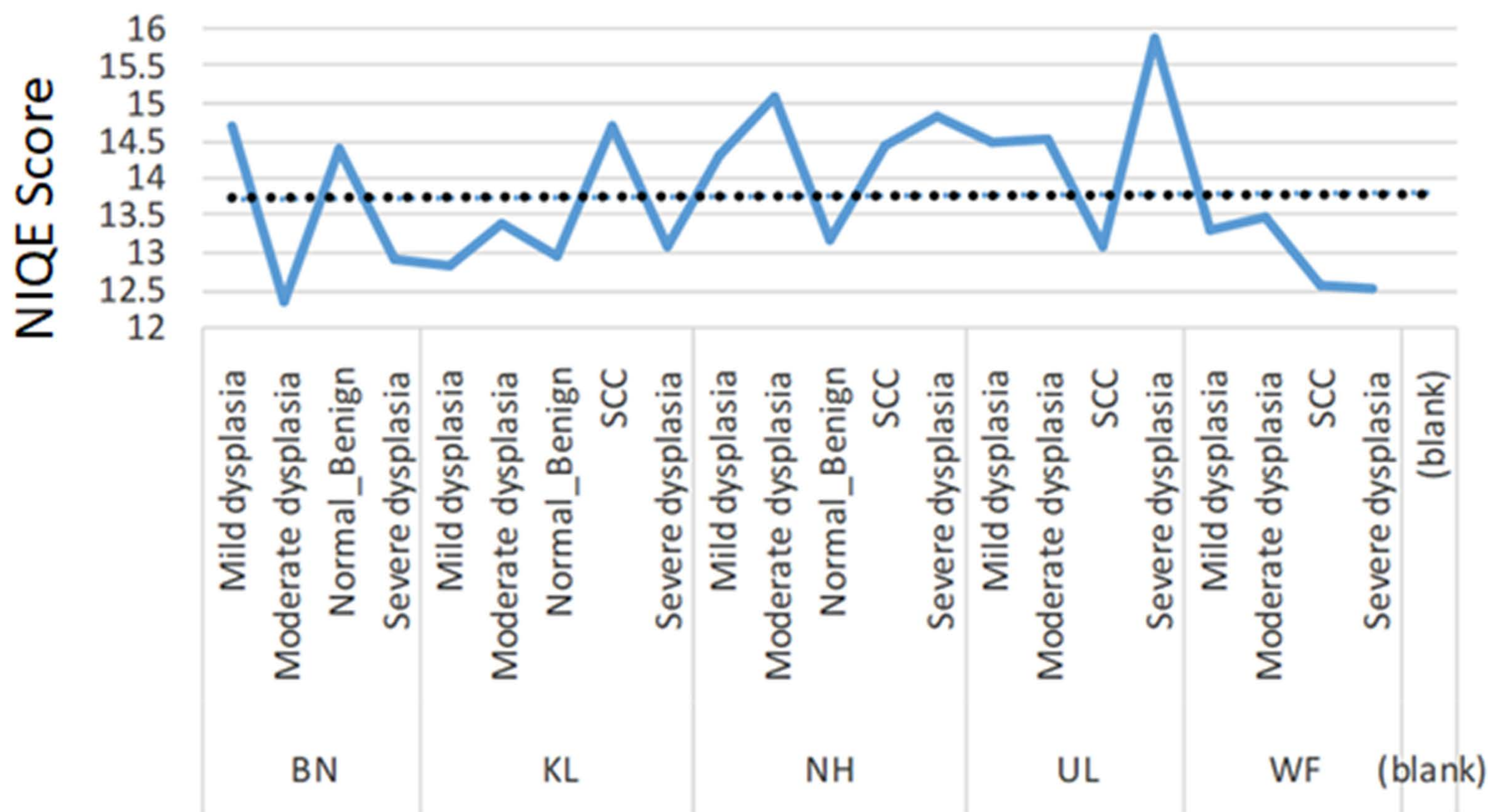

Supplement: Supplementary file 1 [file cancers-13-03583-s001.zip › Figure S1.pdf]

Sub-sites

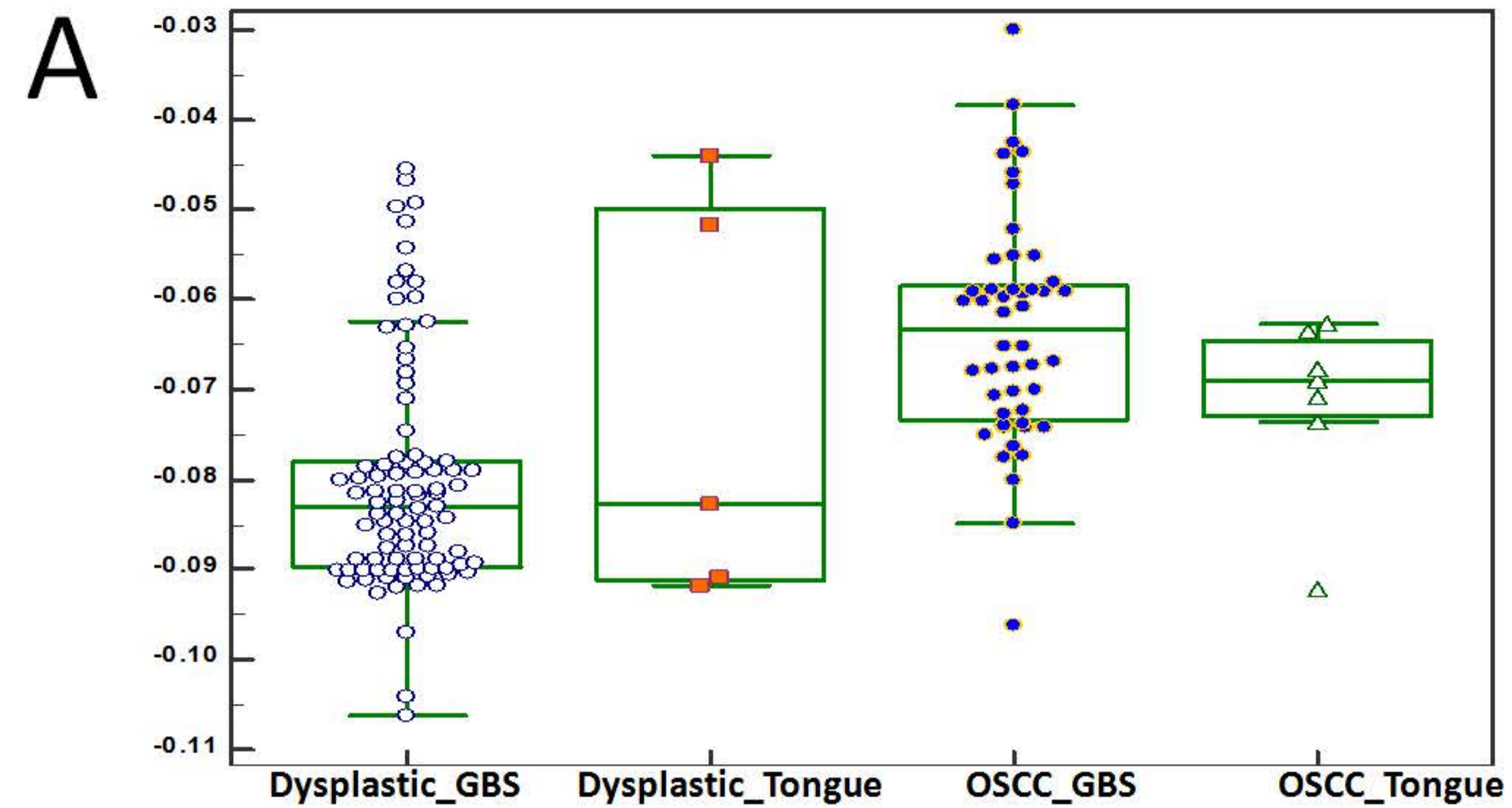

Gender

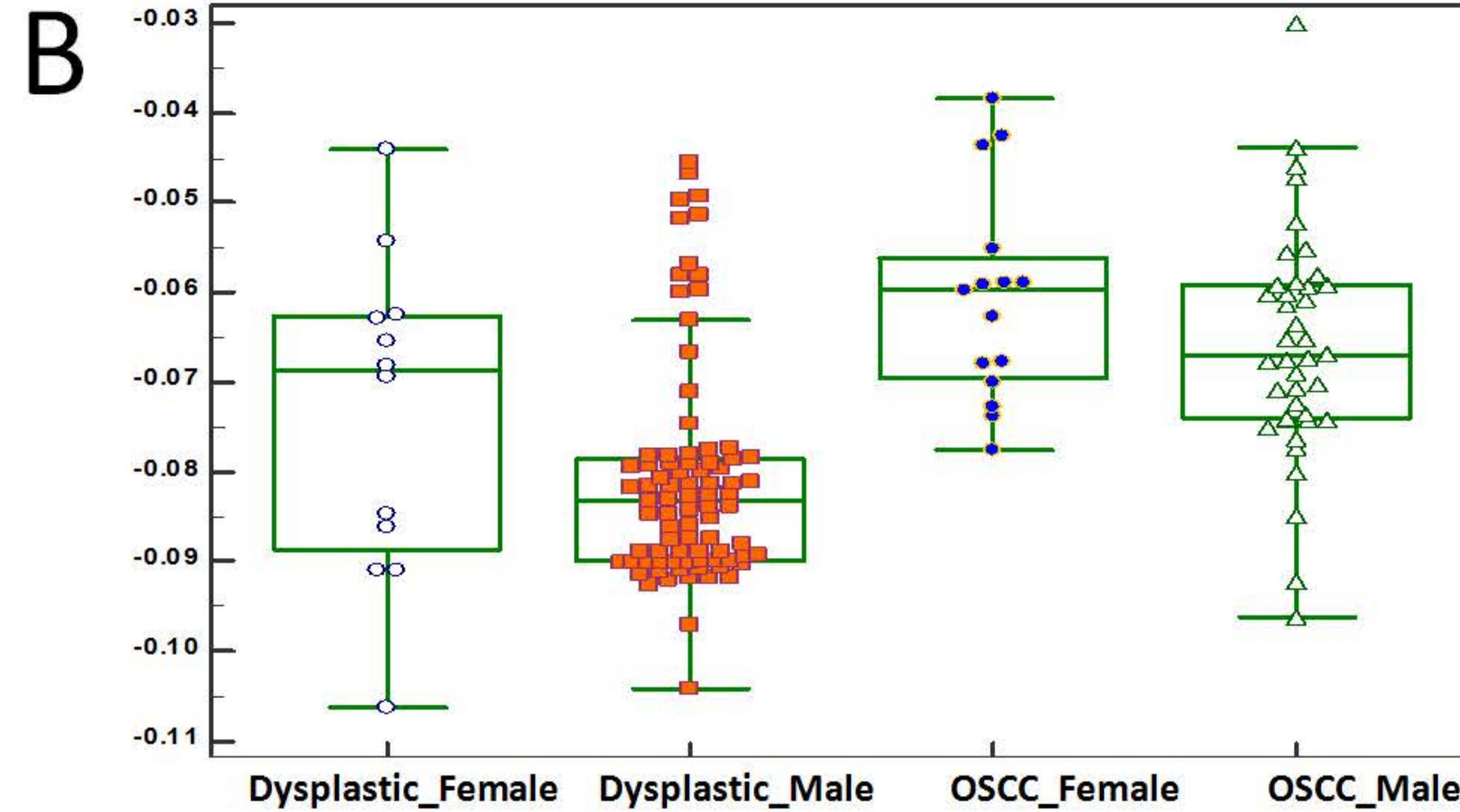

Age

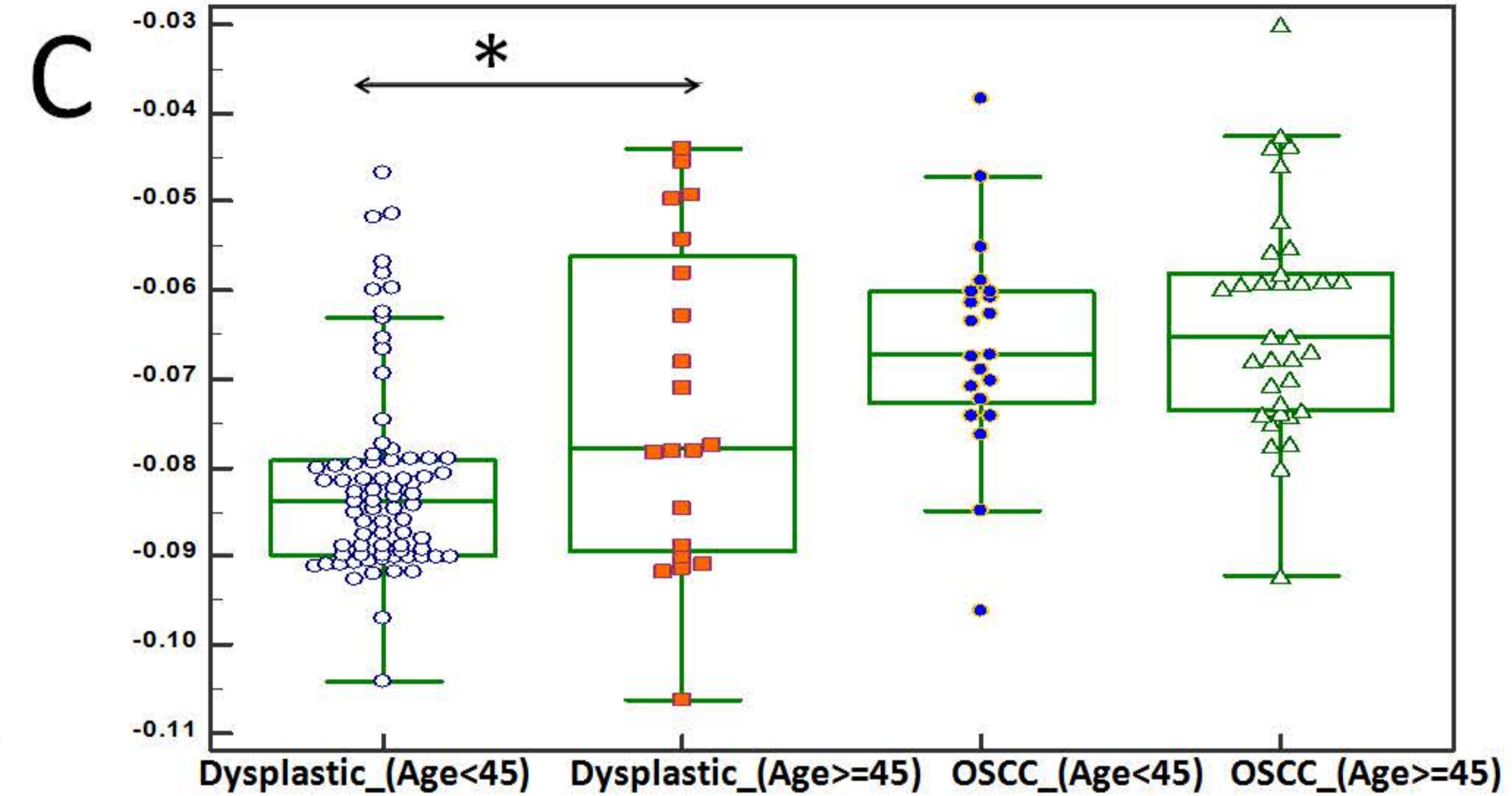

Habit history

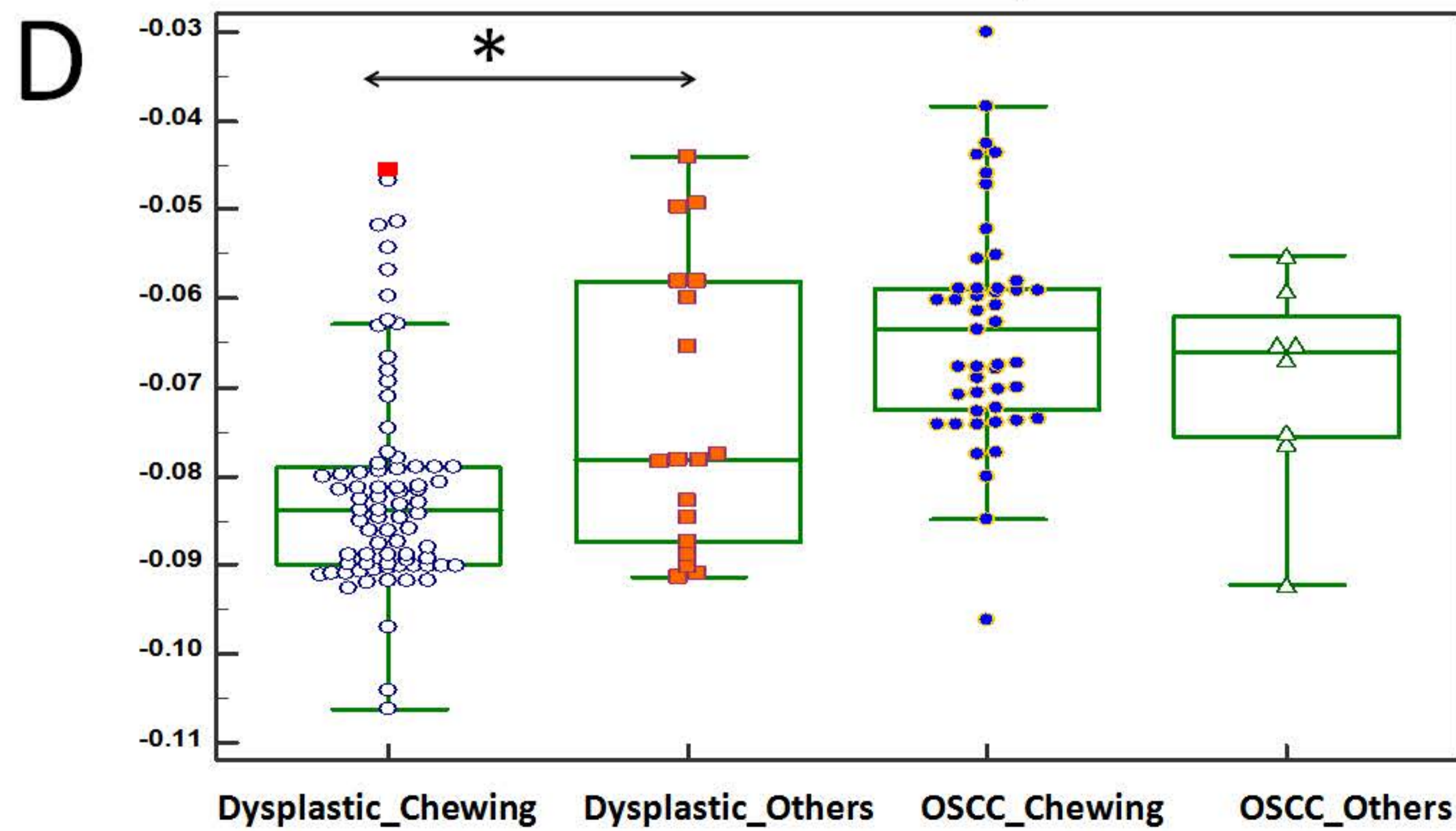

Grades of dysplasia

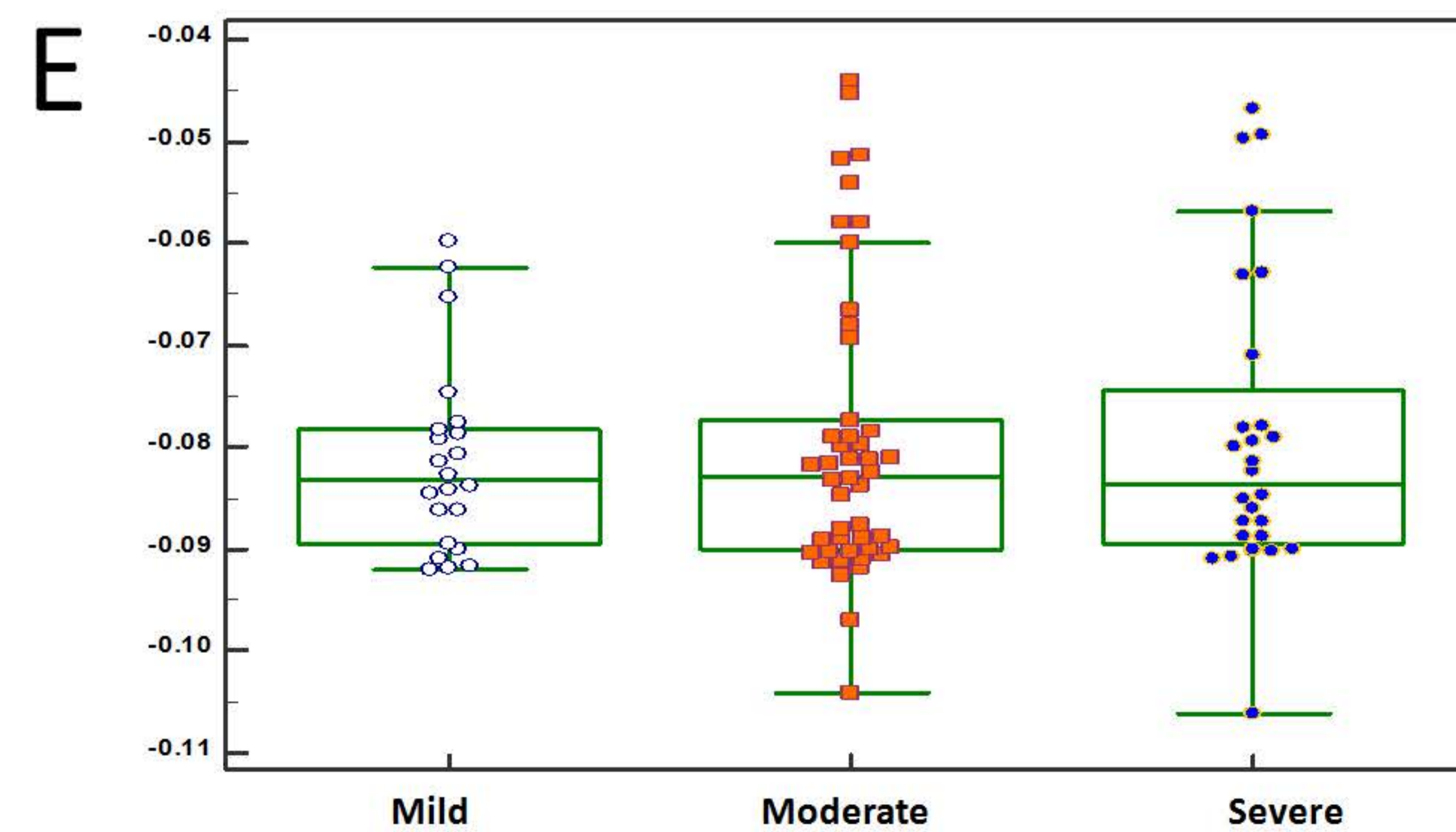

HGD Vs LGD

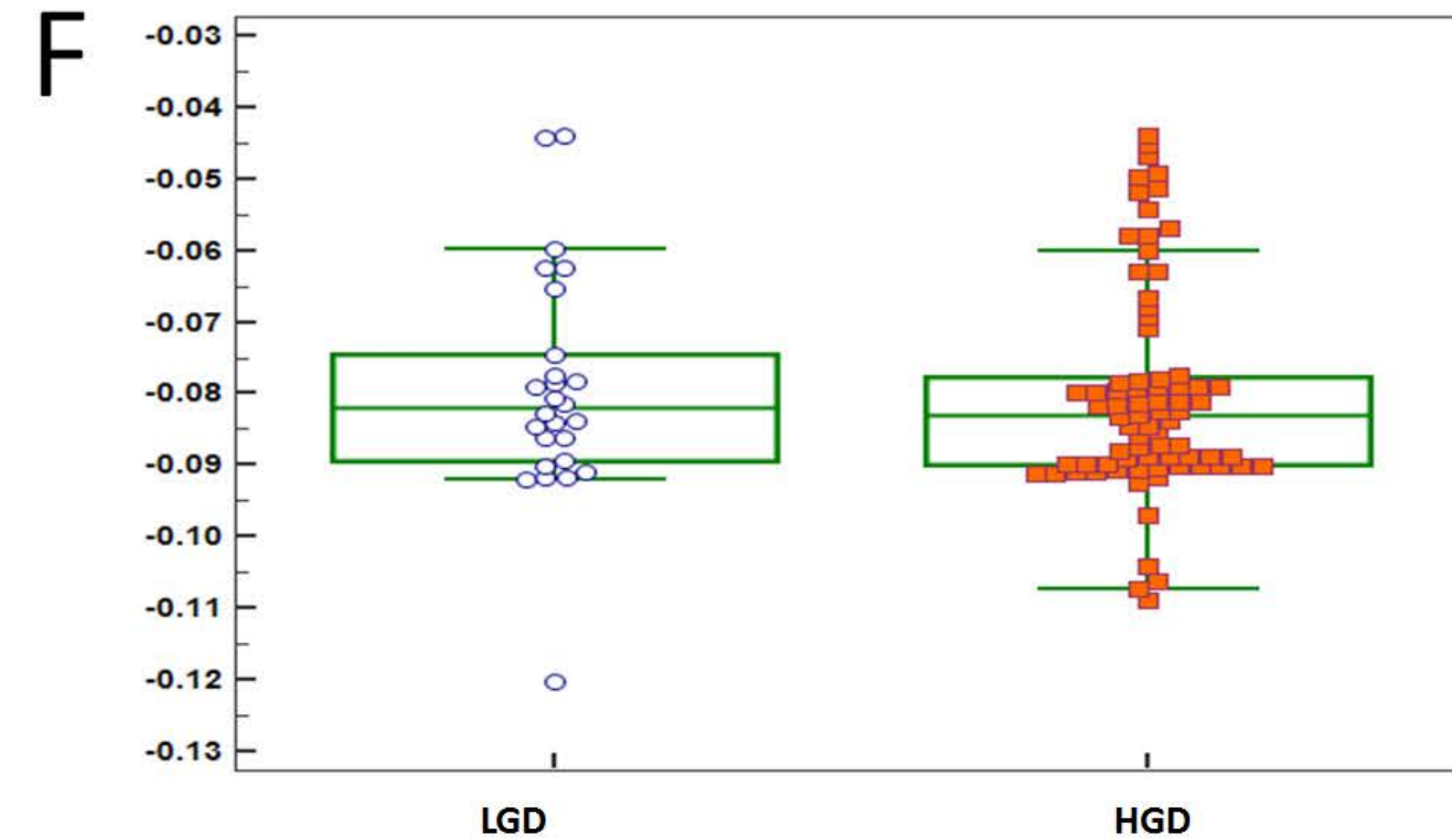

Supplement: Supplementary file 1 [file cancers-13-03583-s001.zip › Figure S4.pdf]

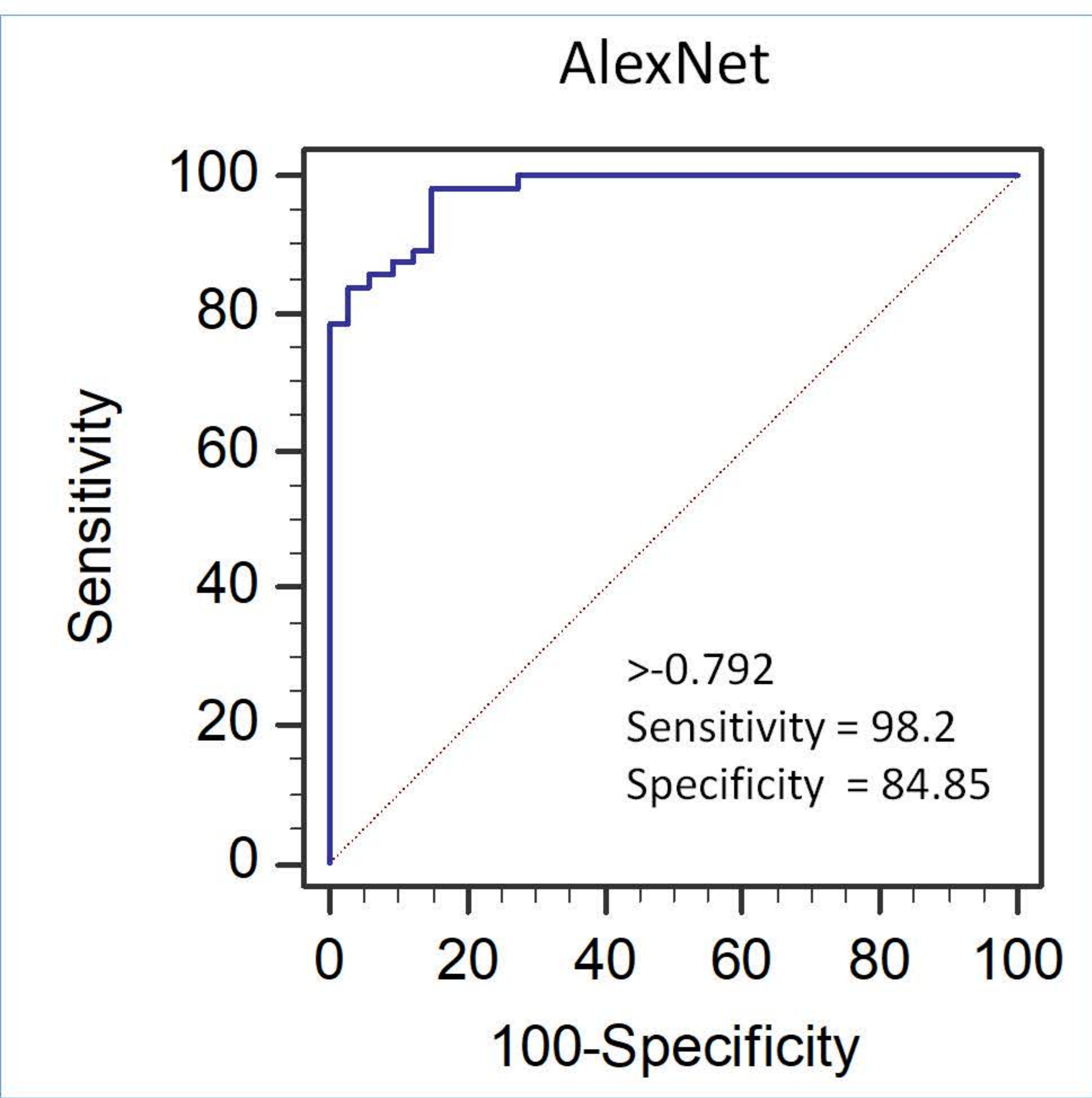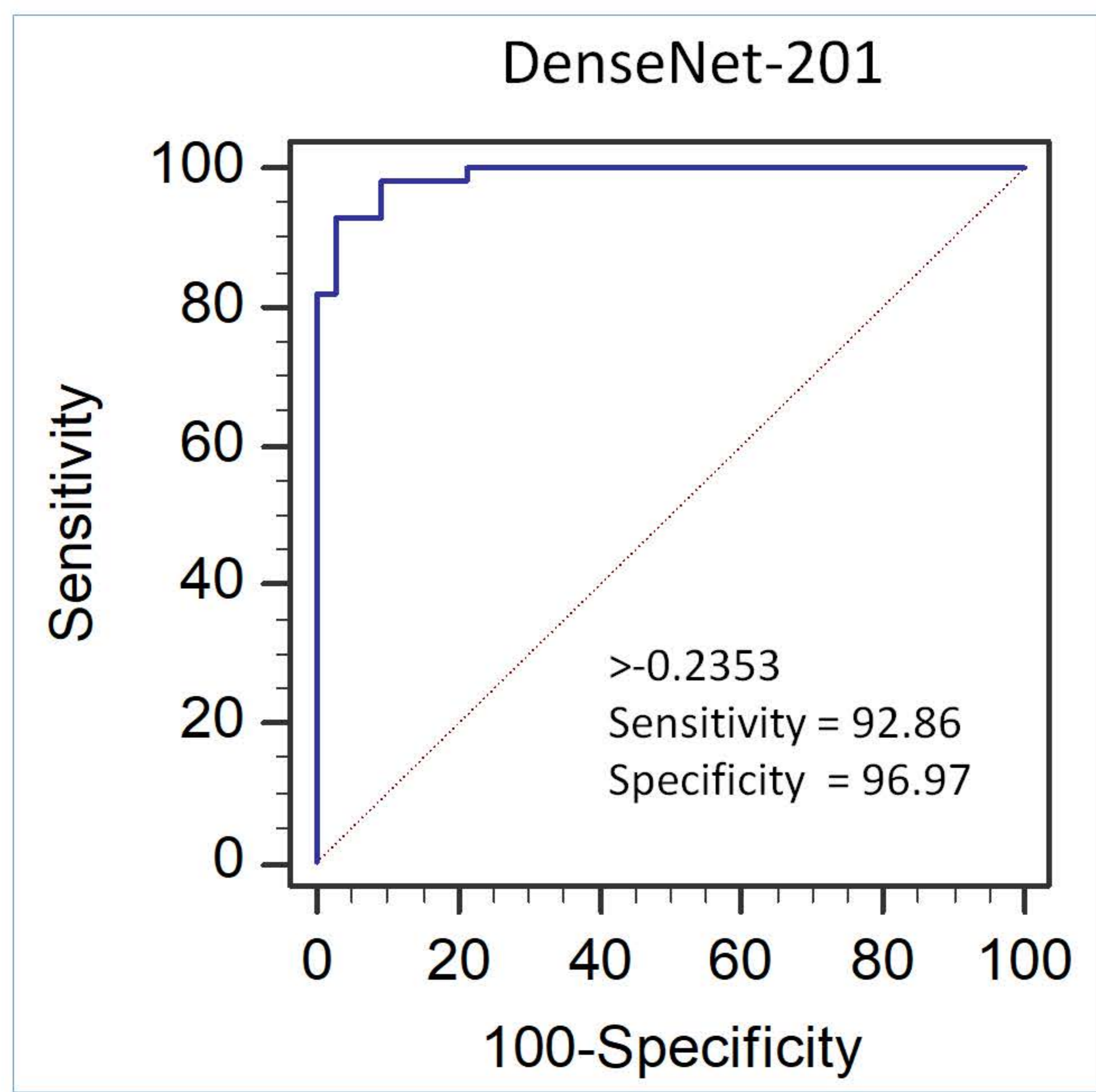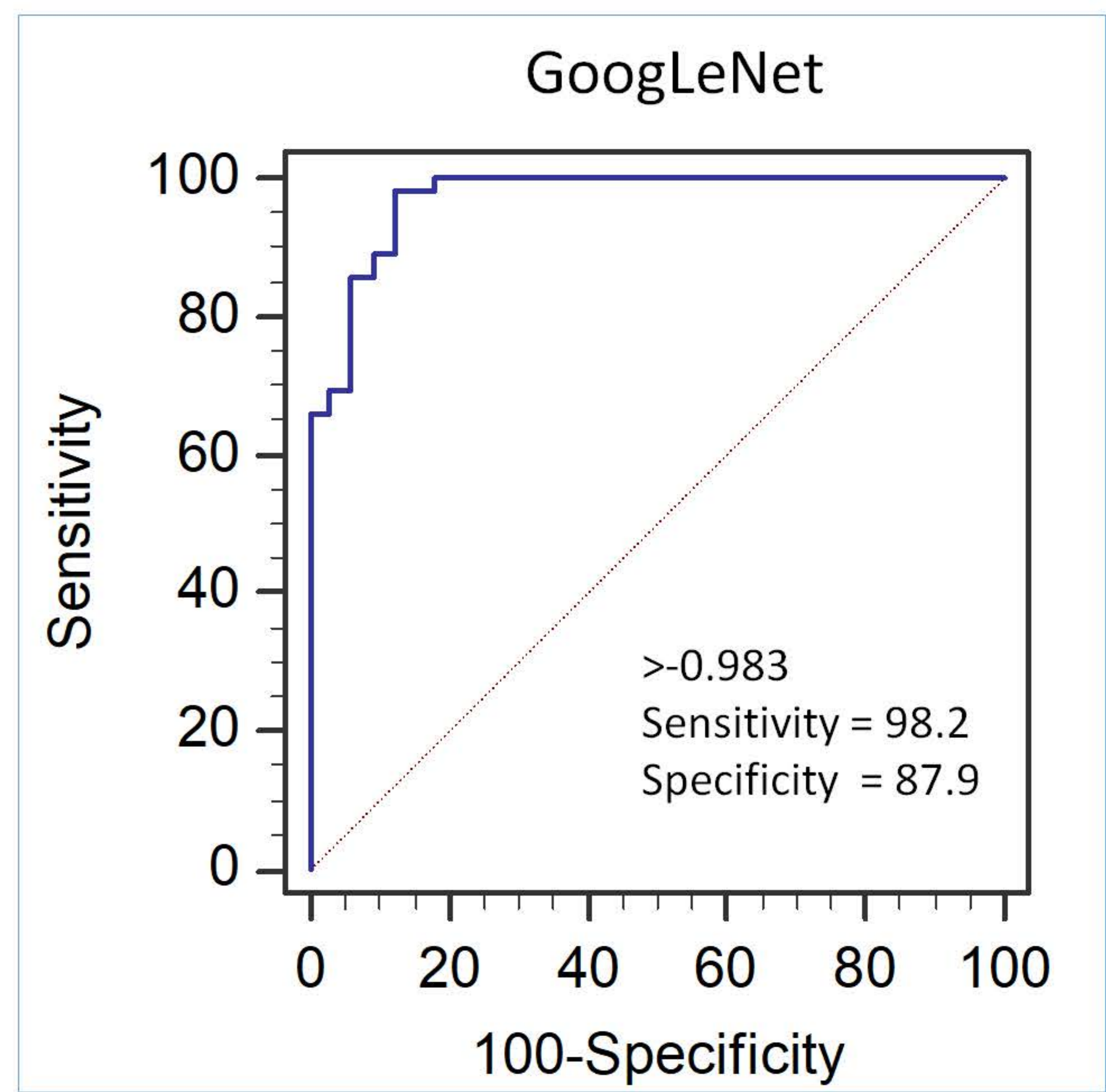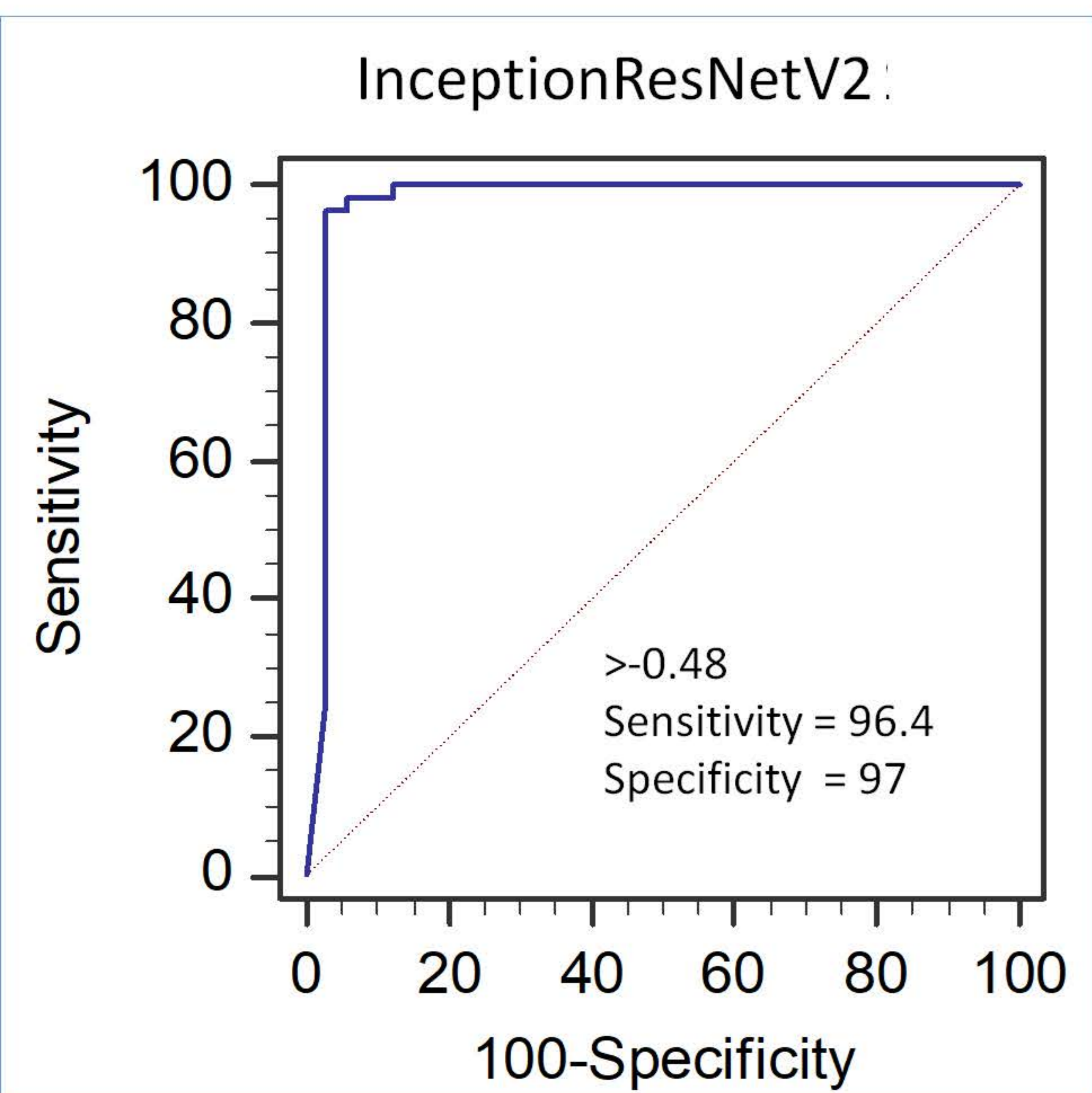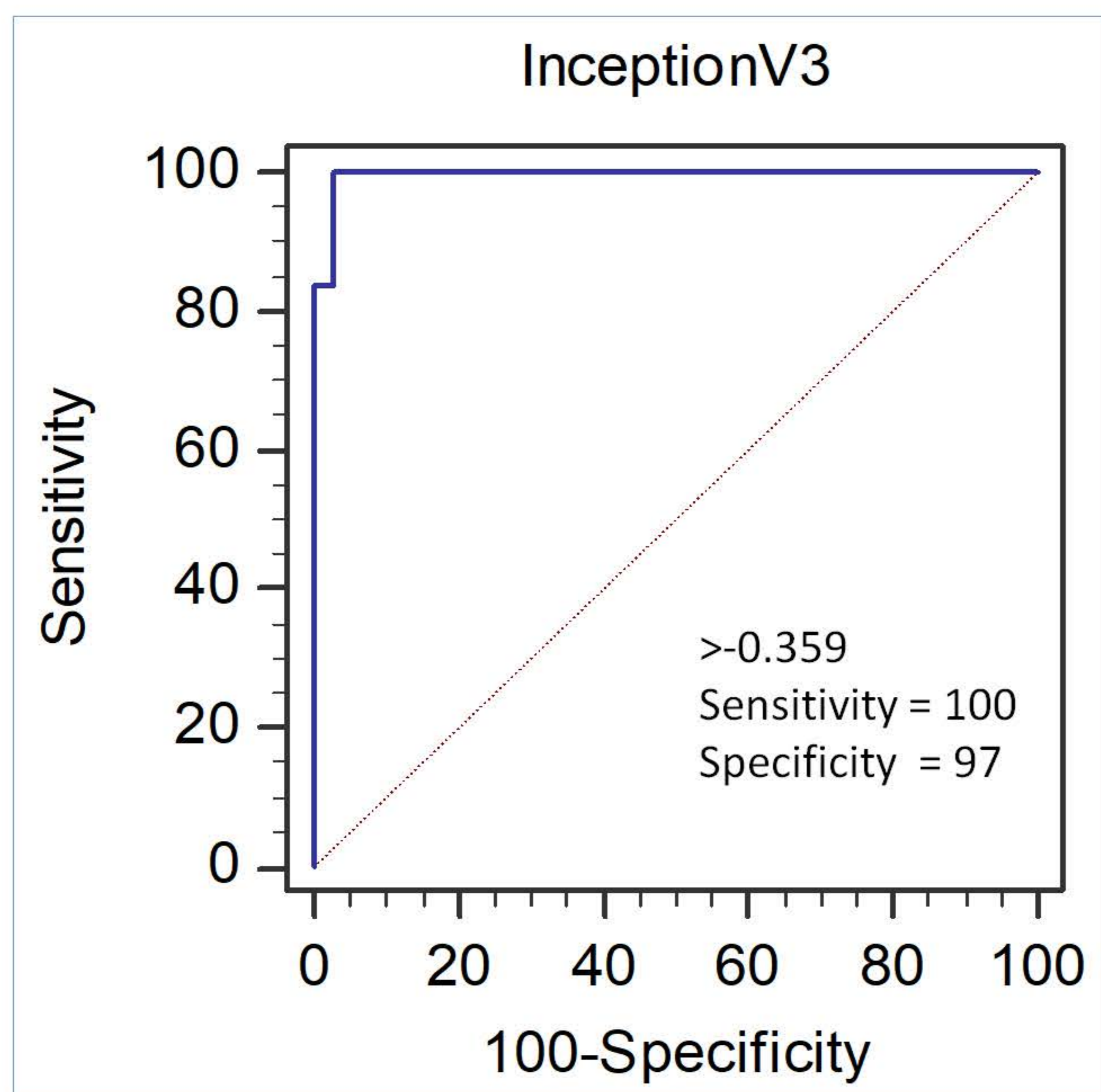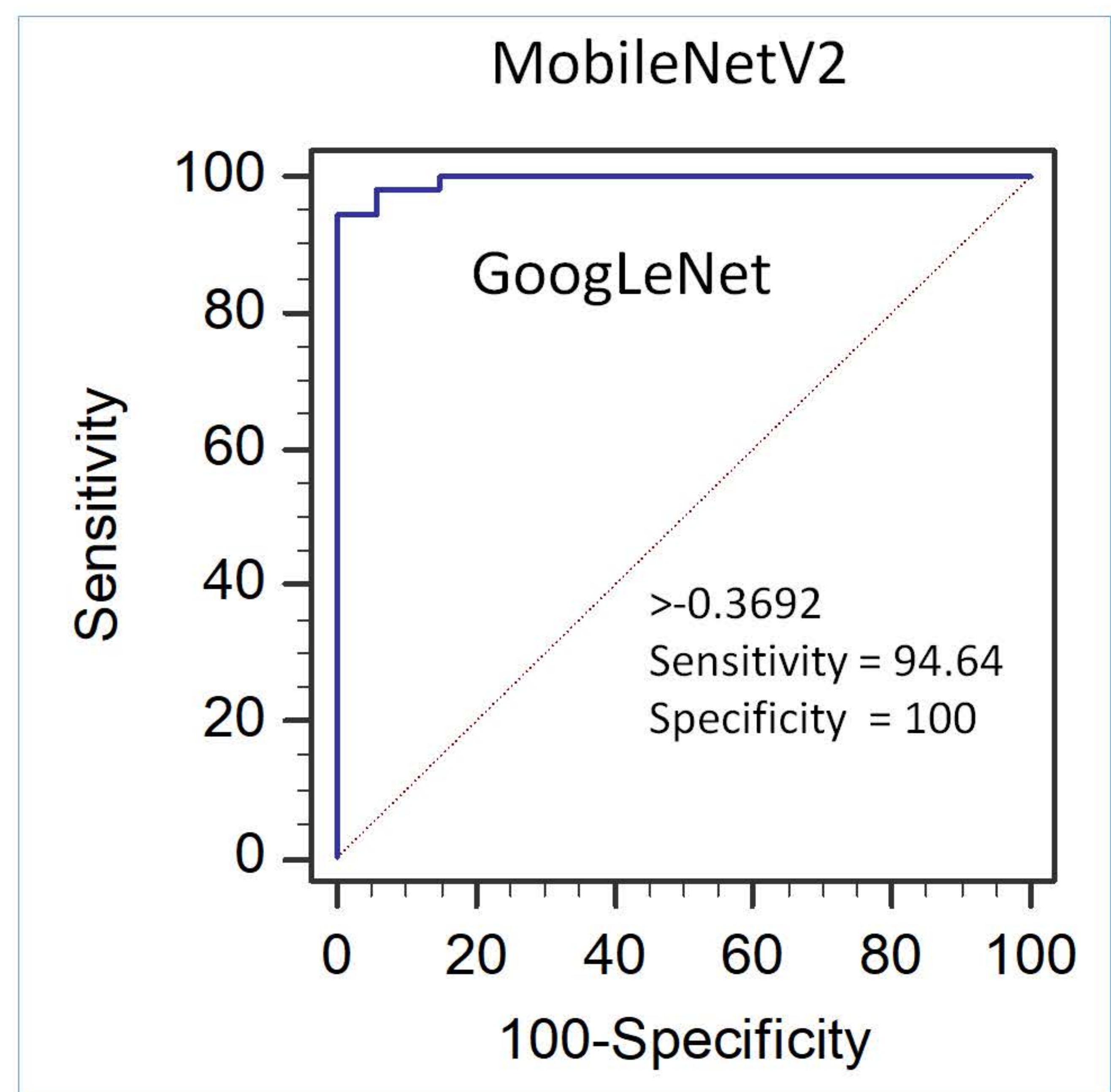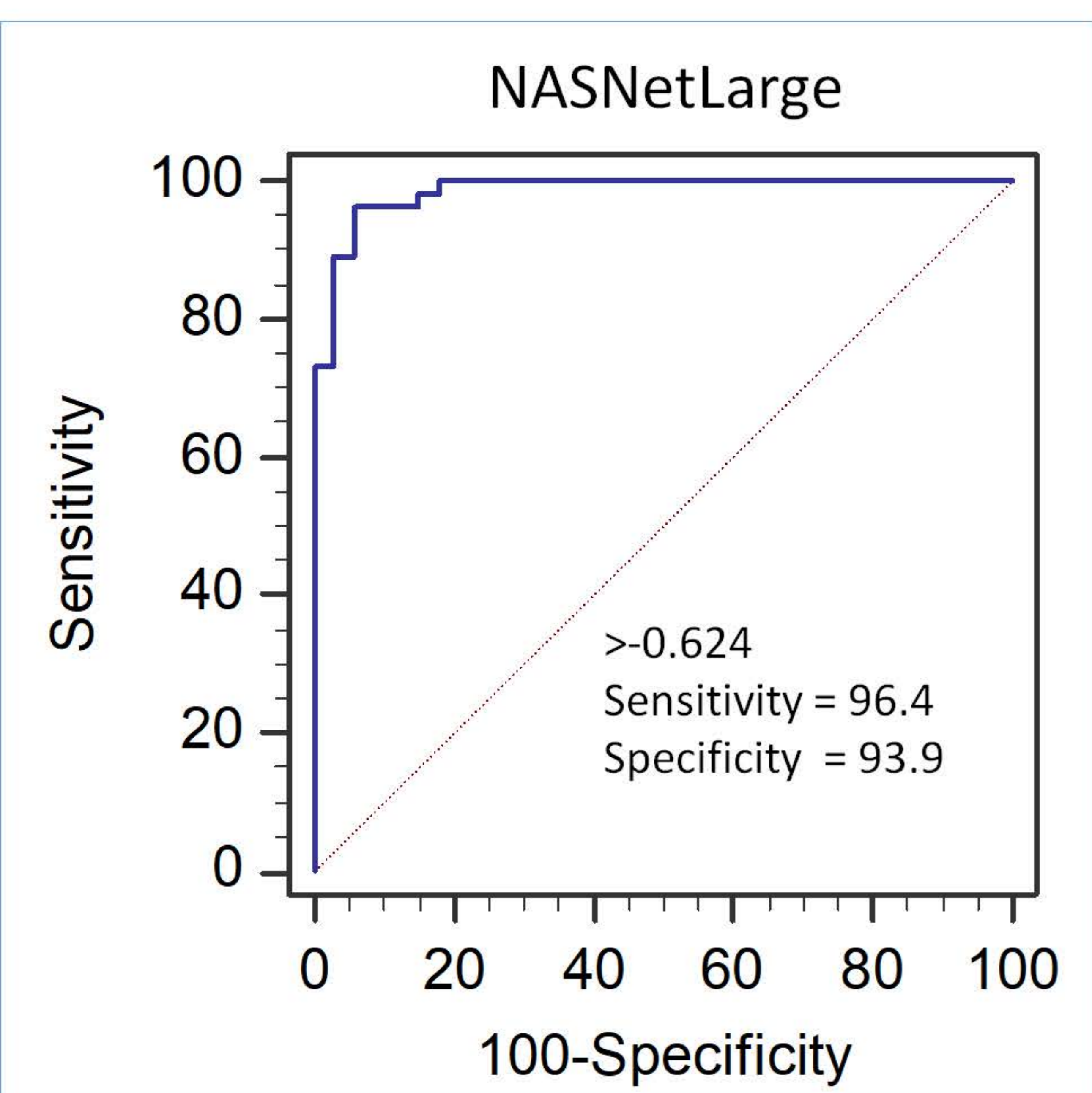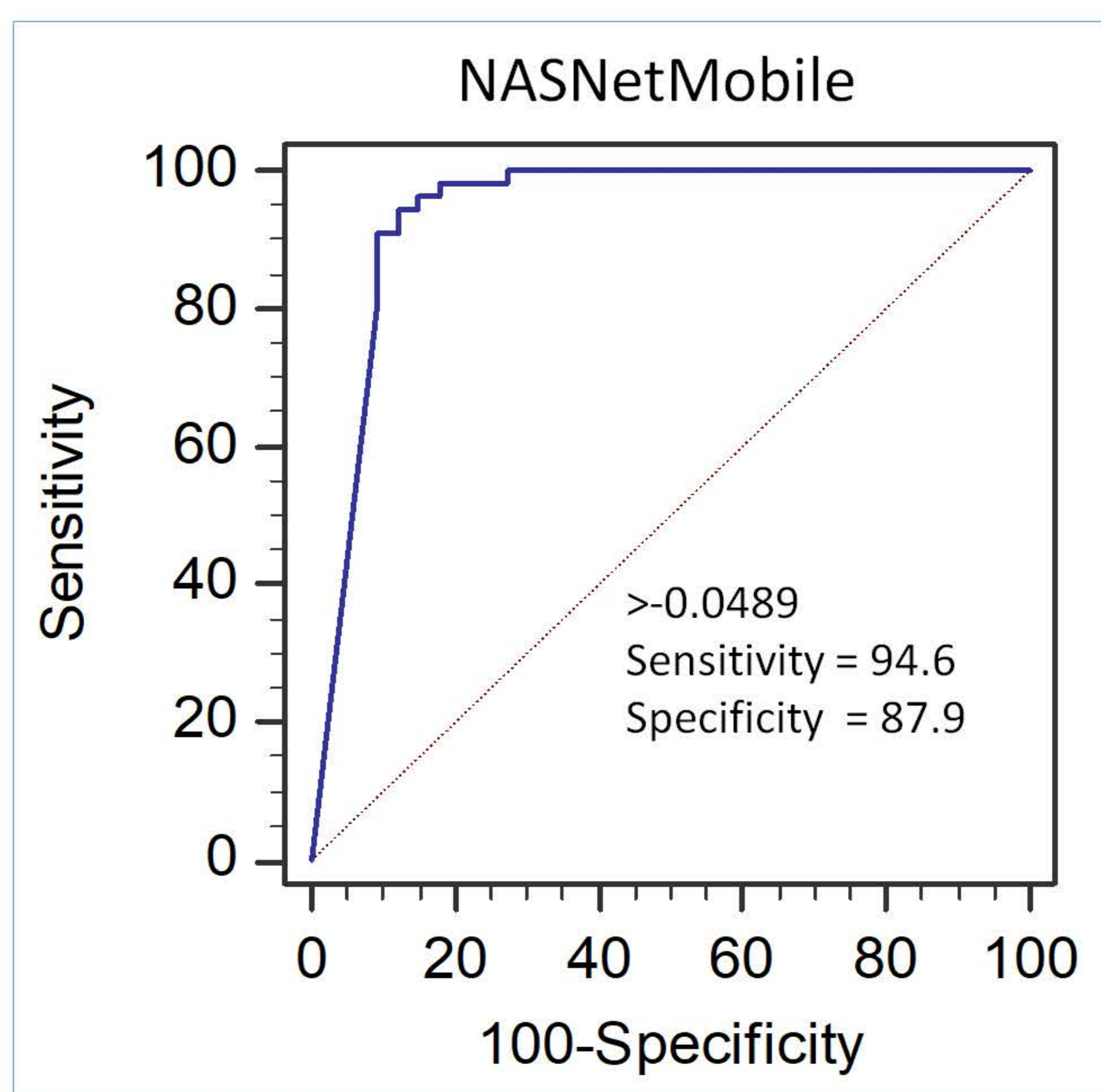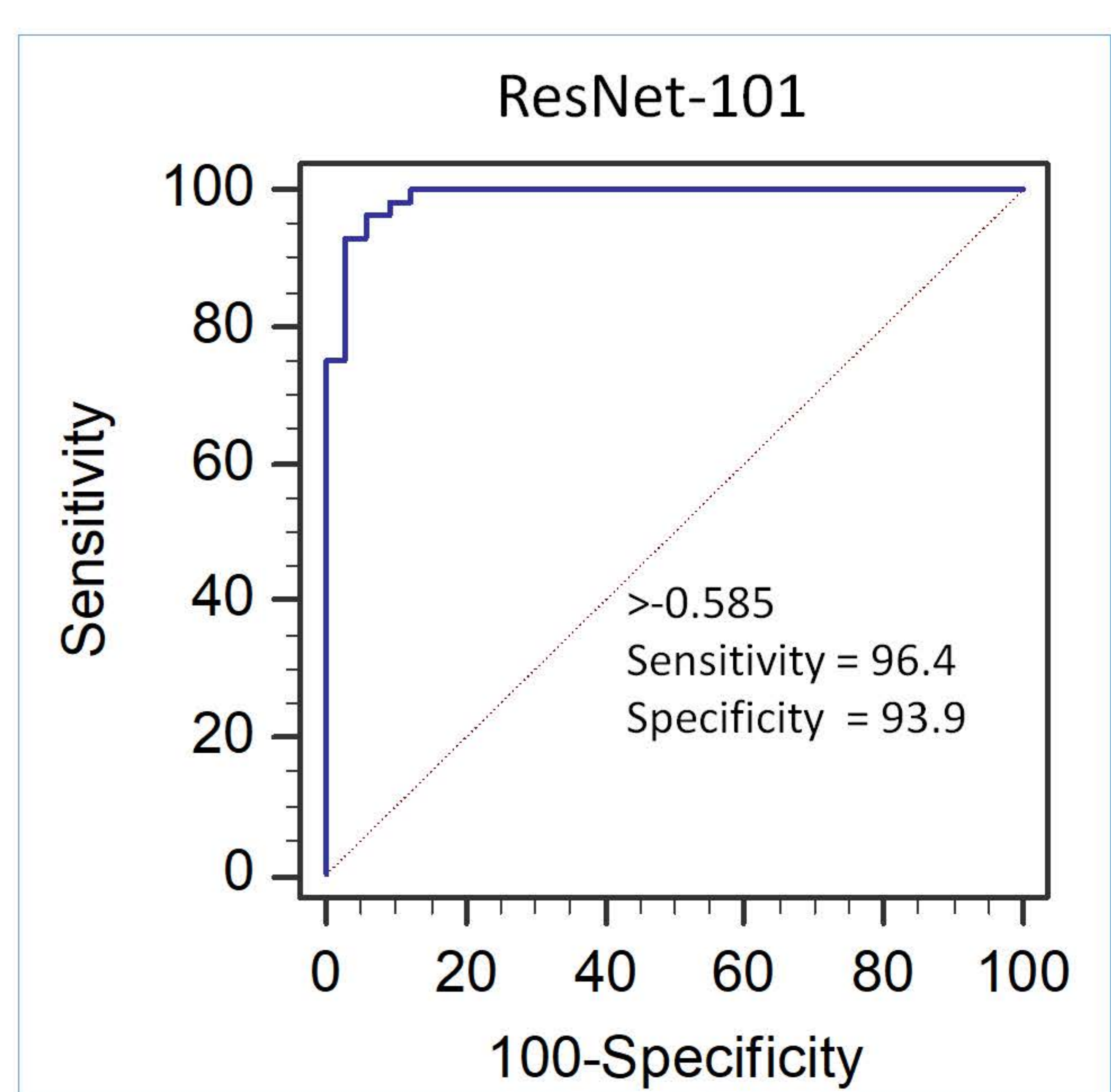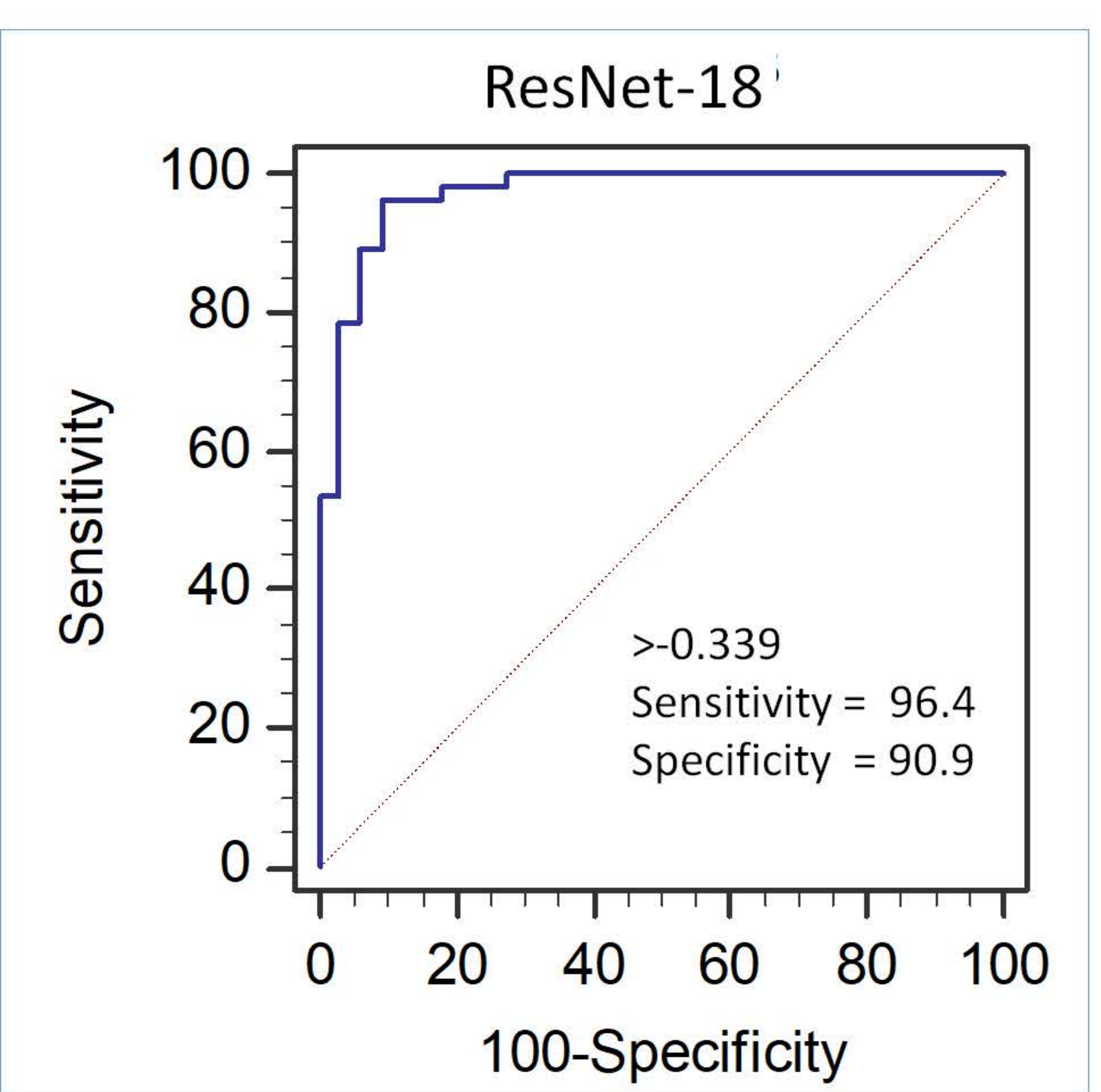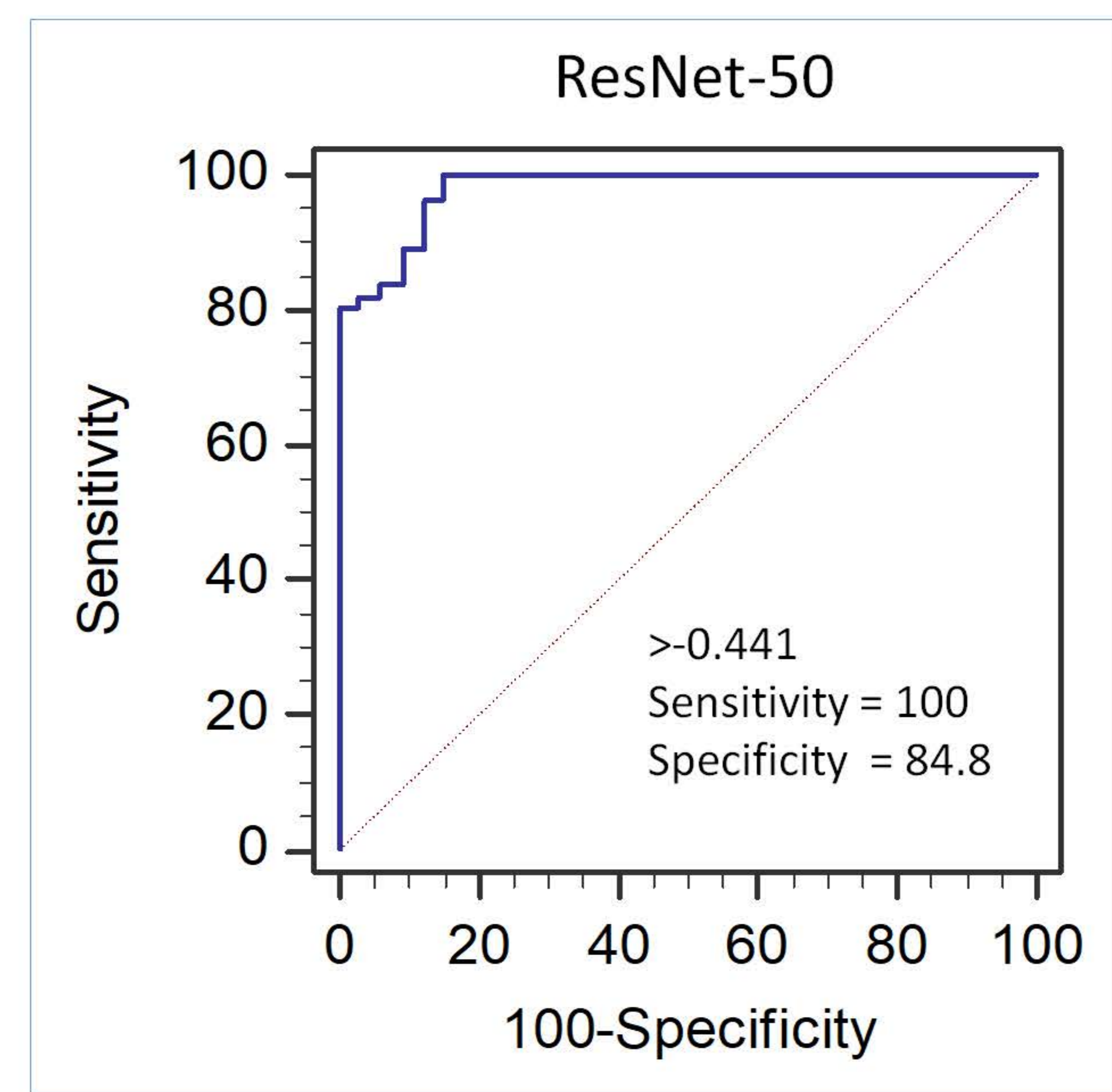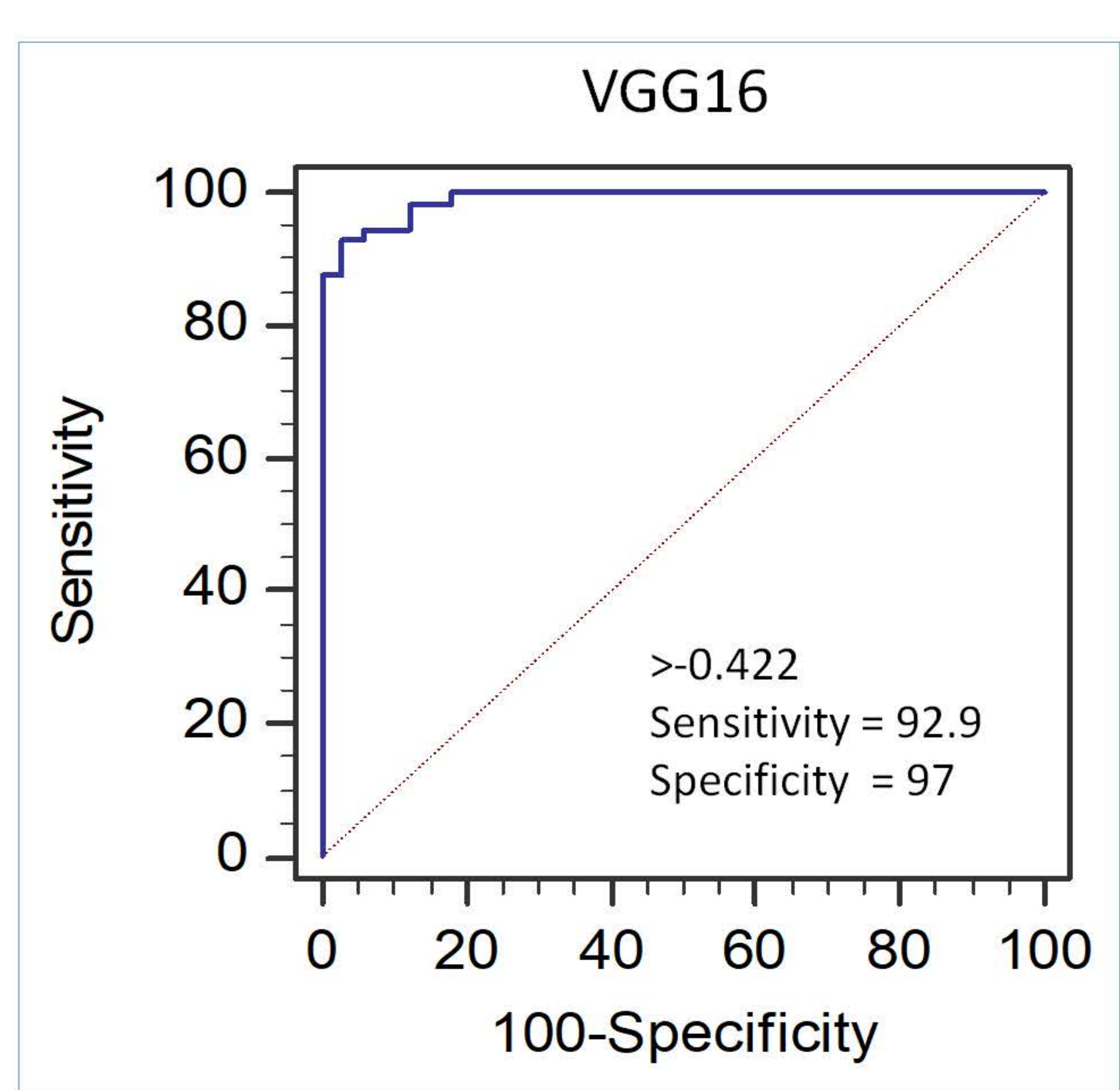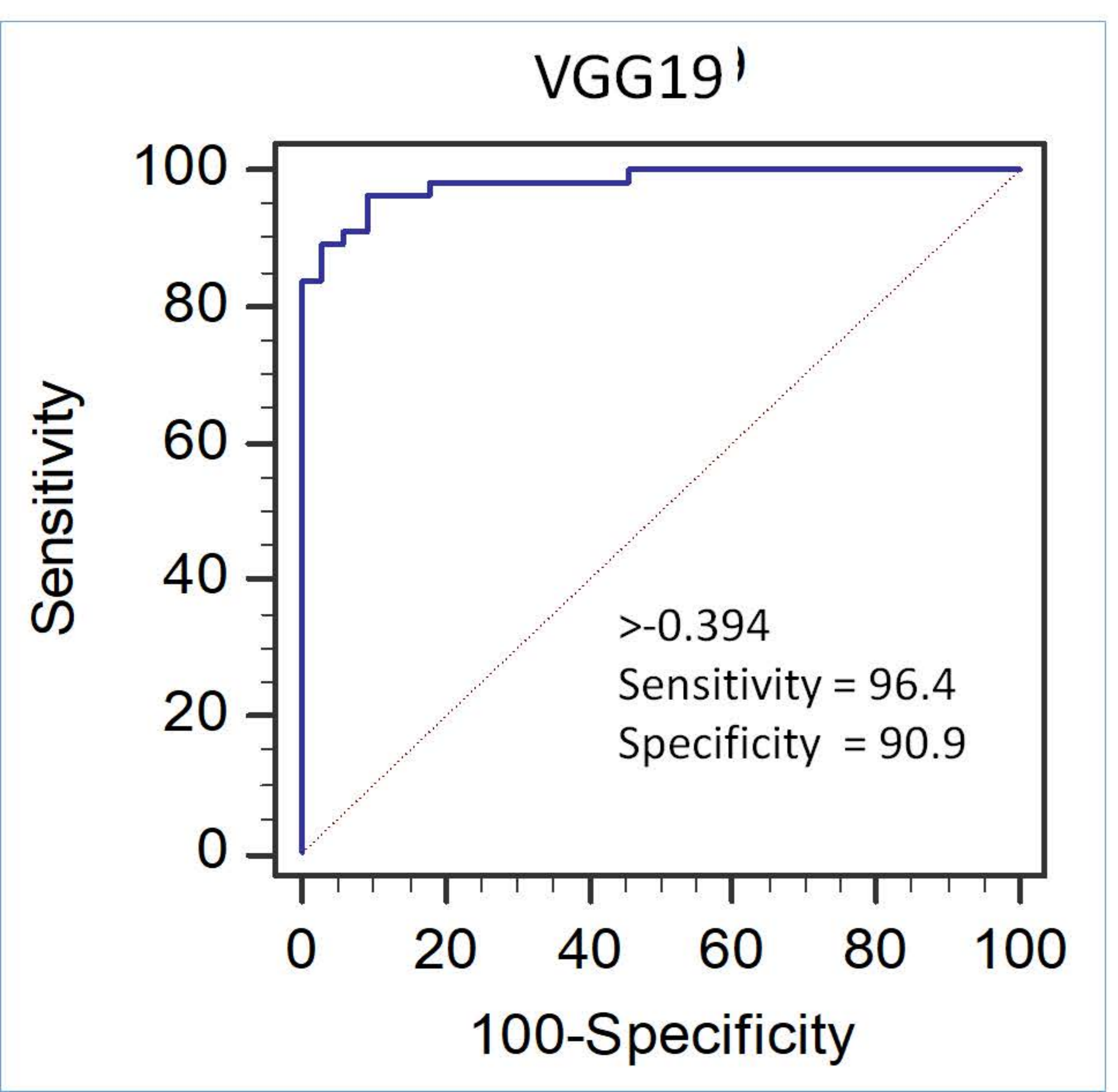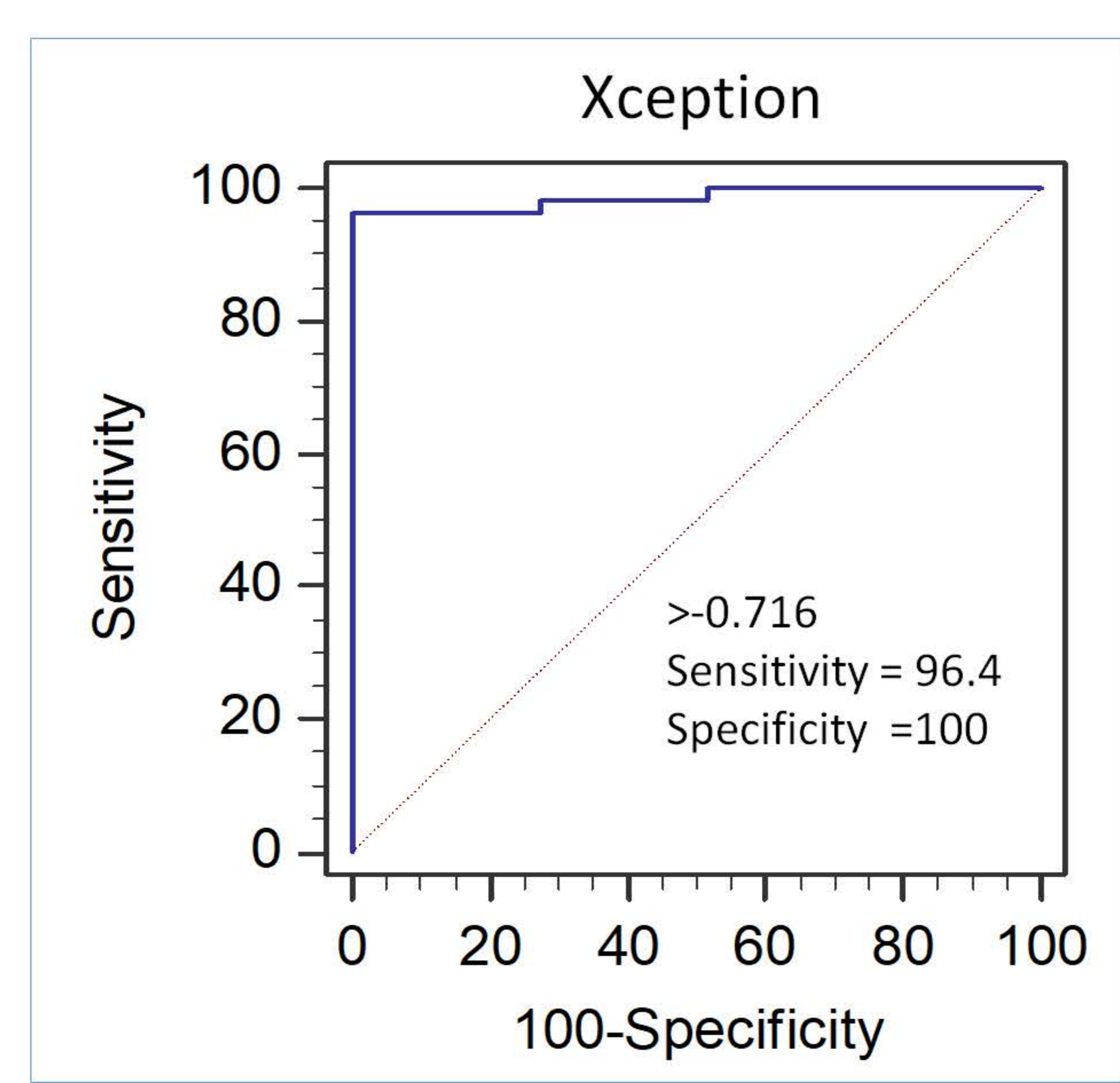

Supplement: Supplementary file 1 [file cancers-13-03583-s001.zip › Figure S6.pdf]

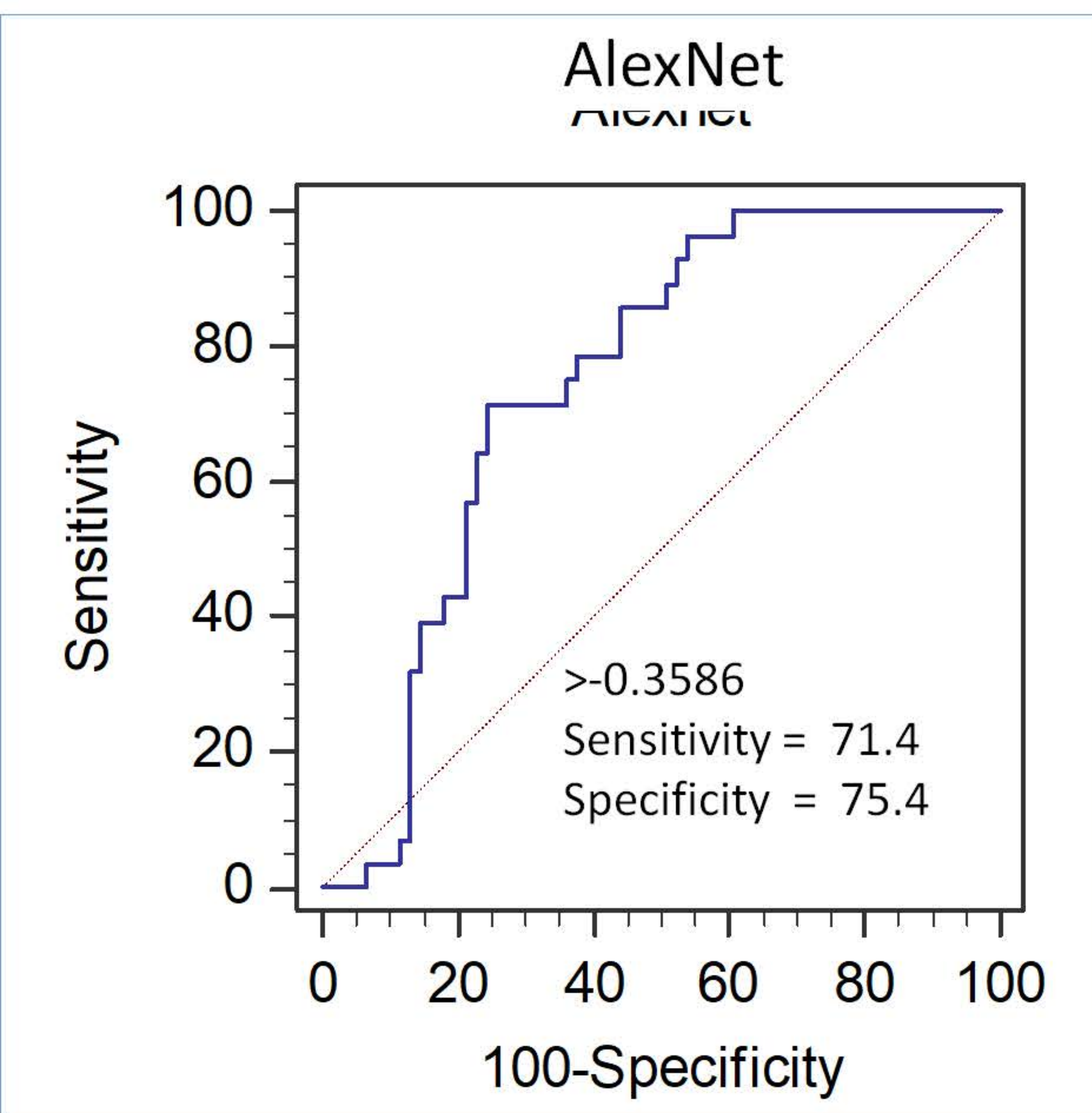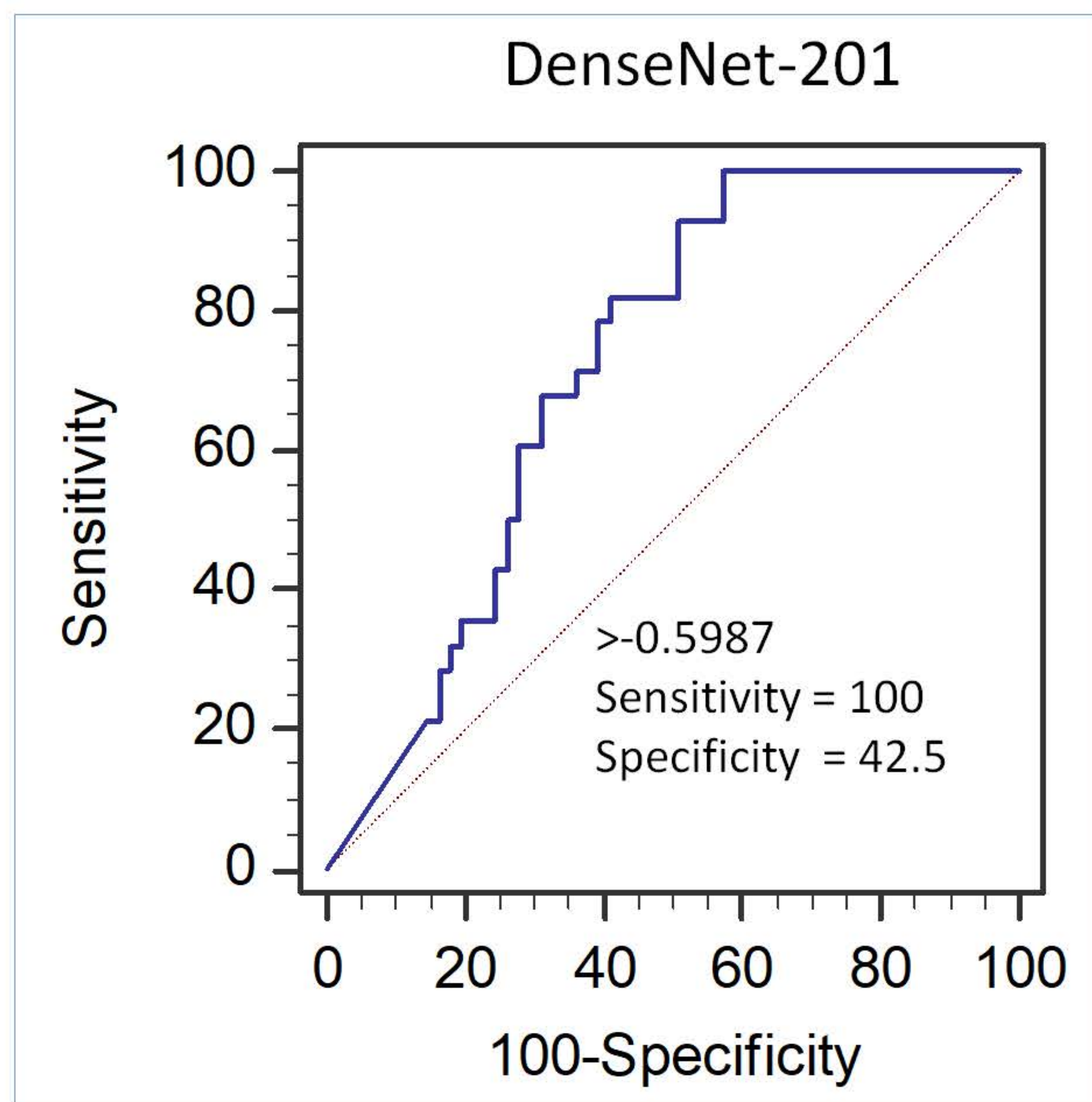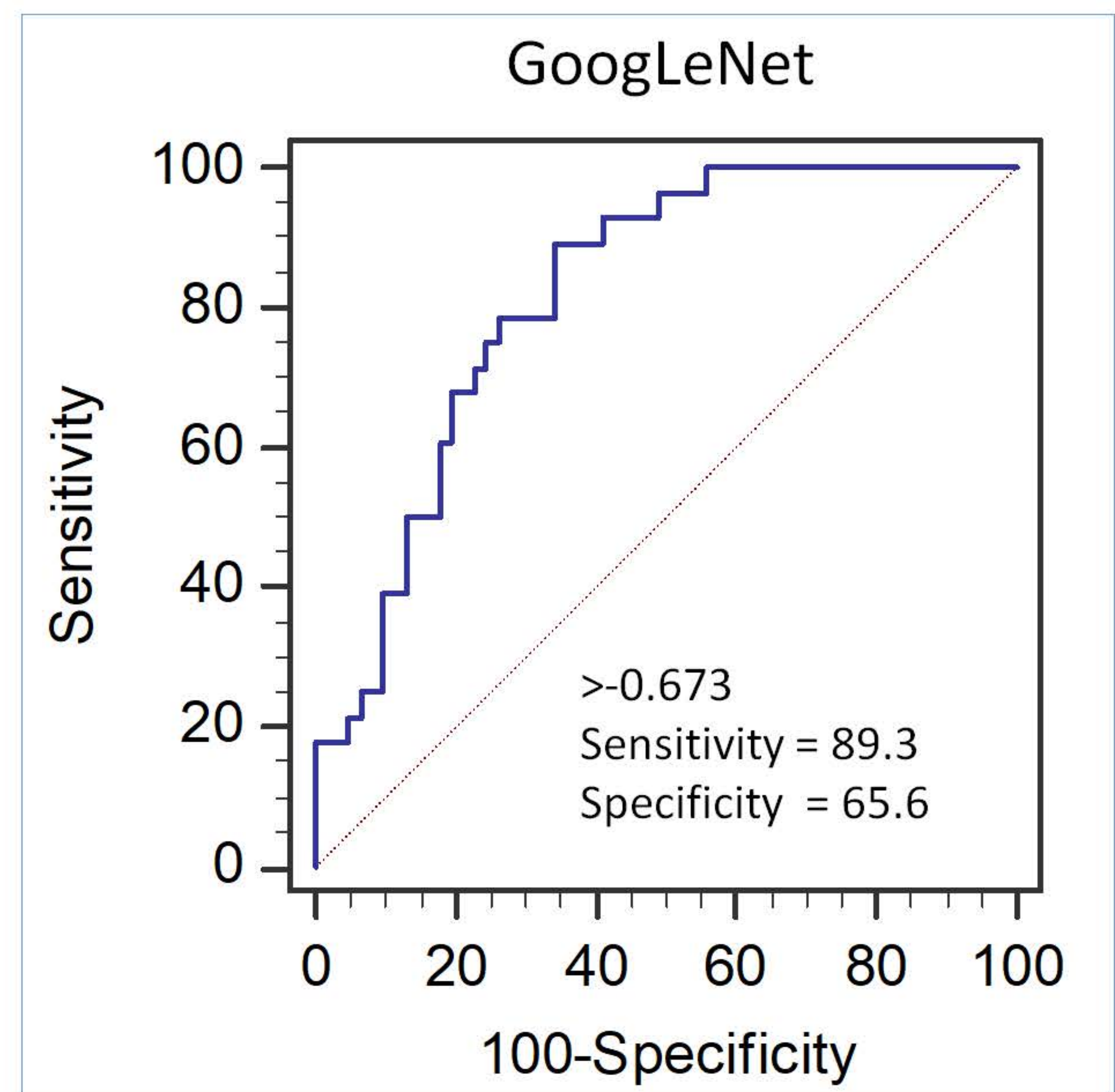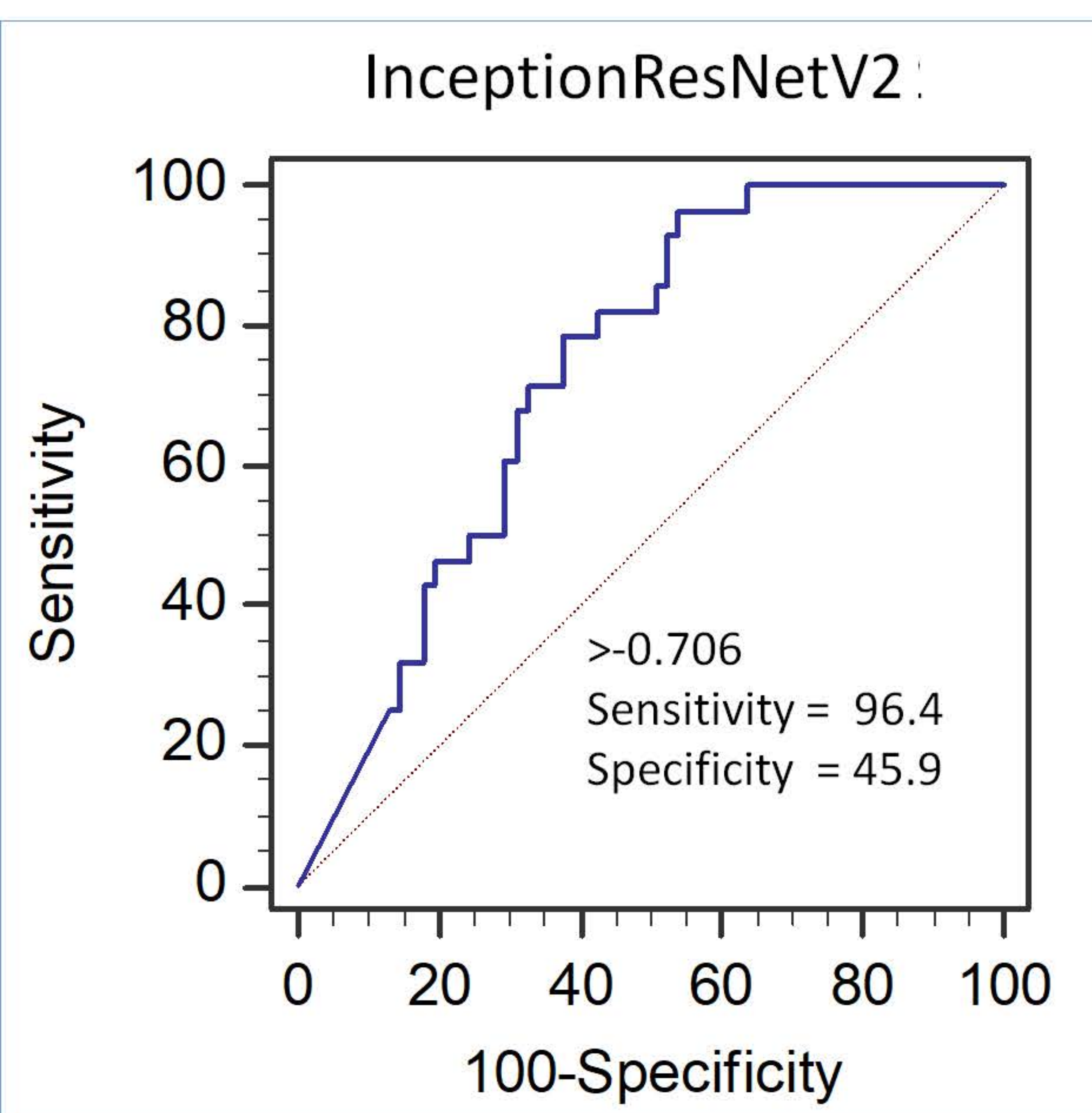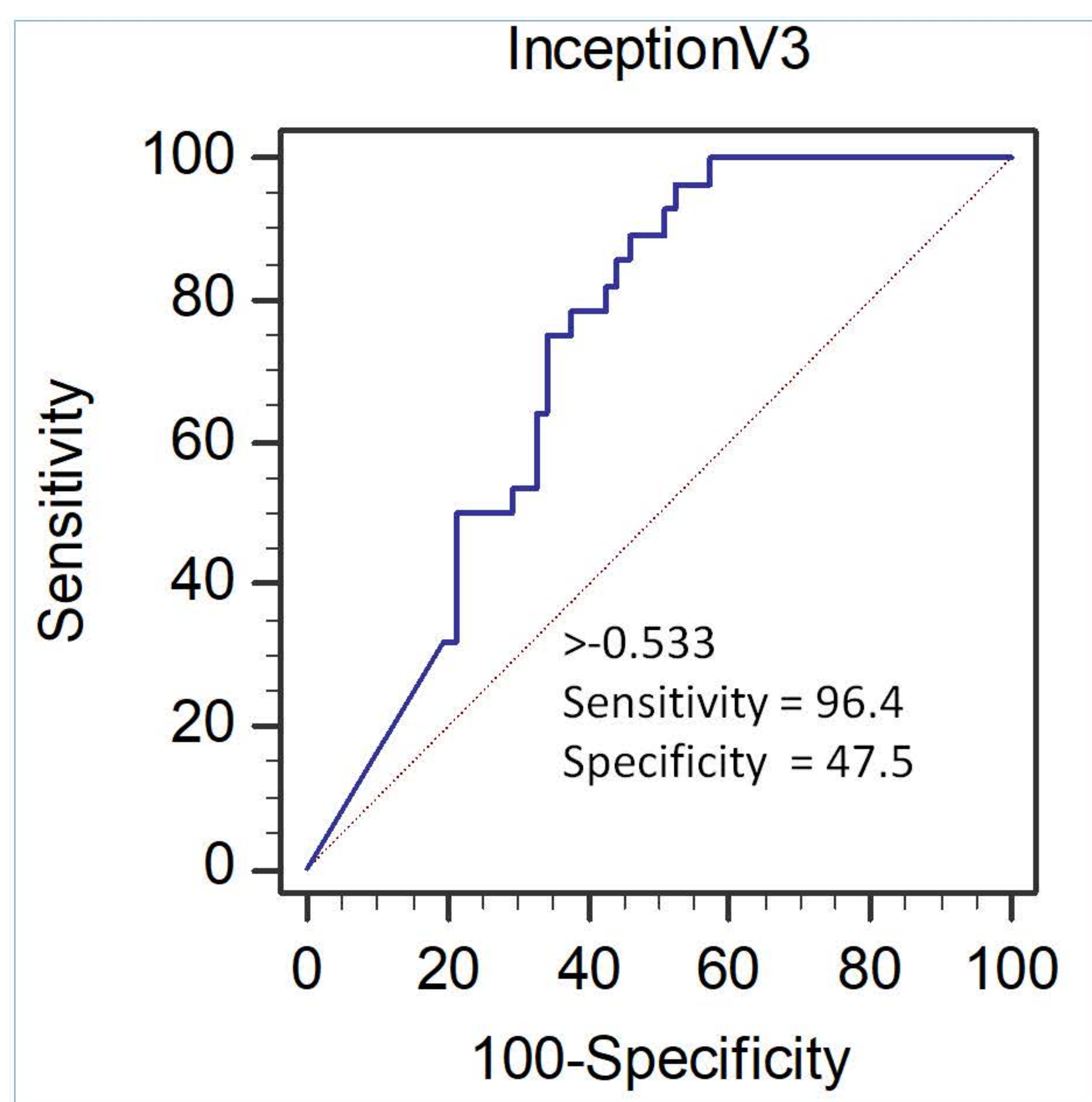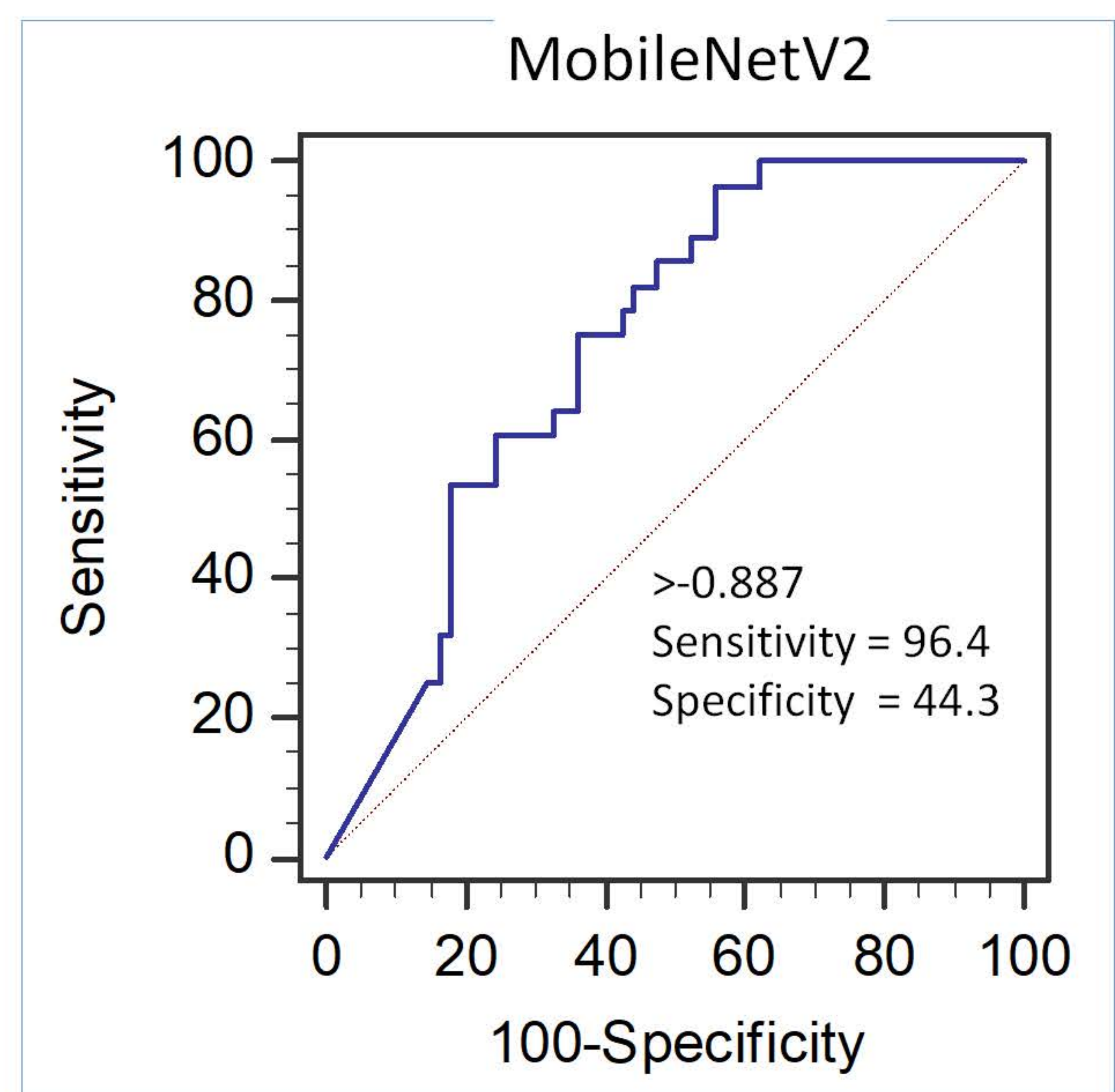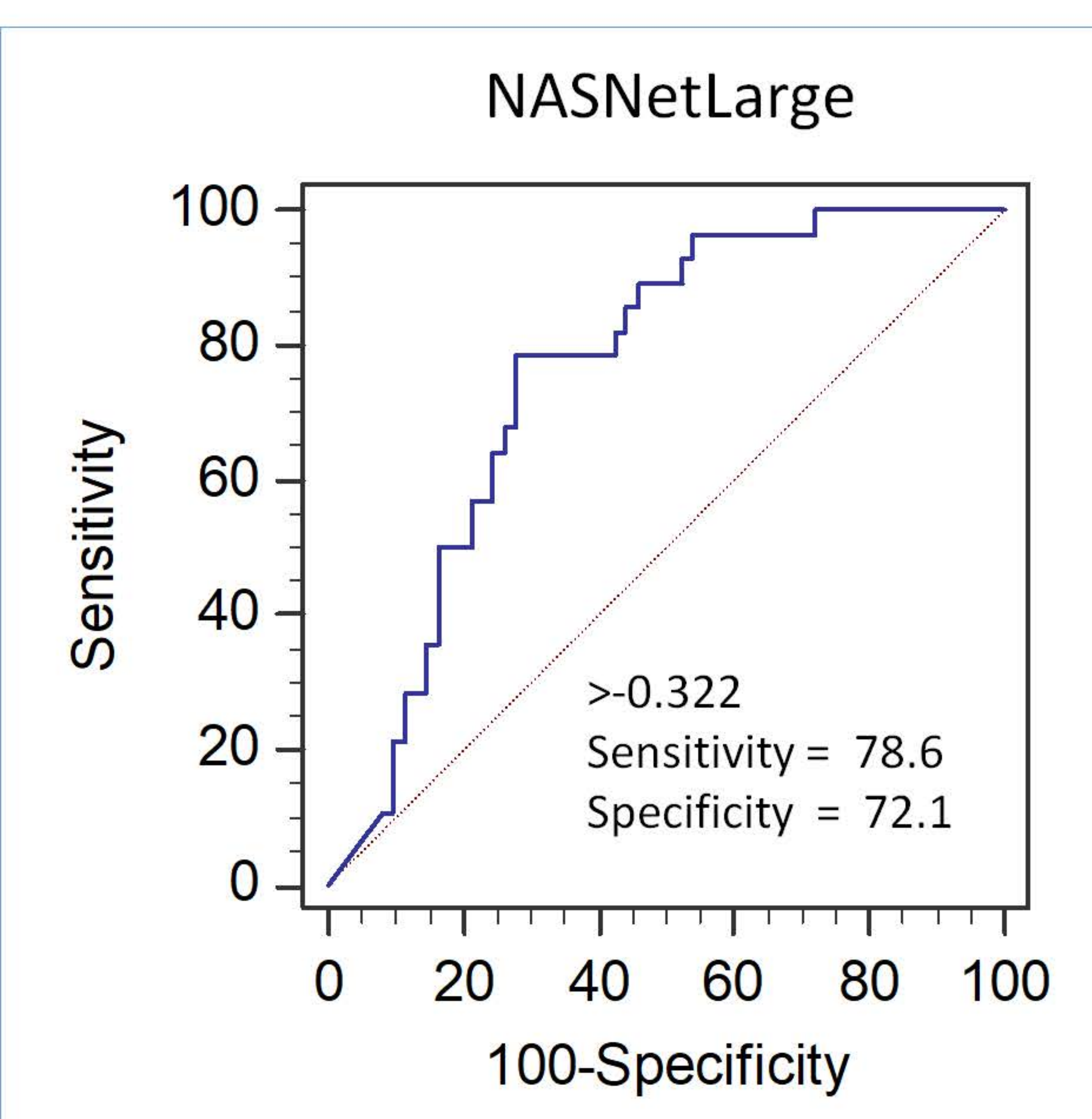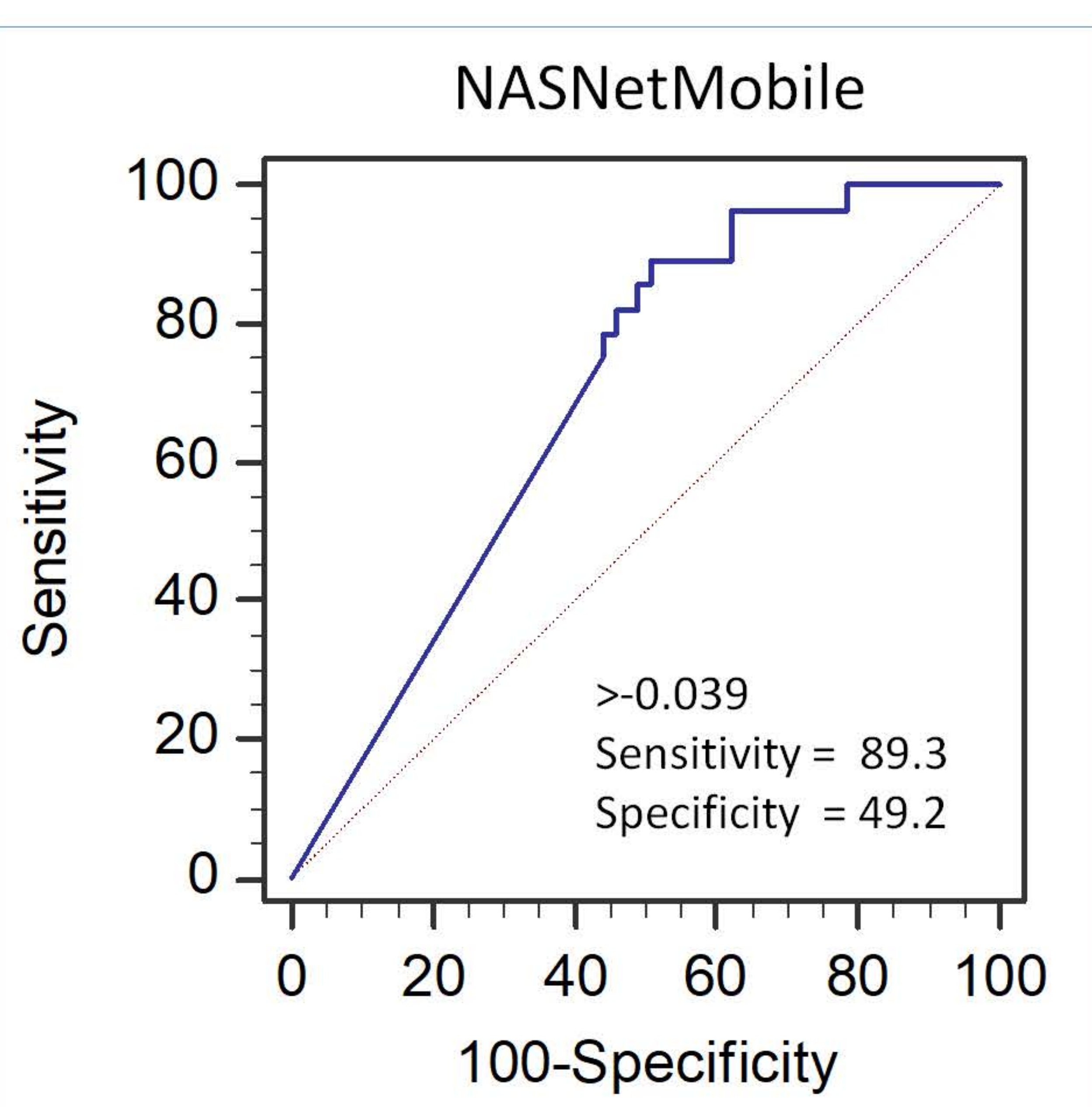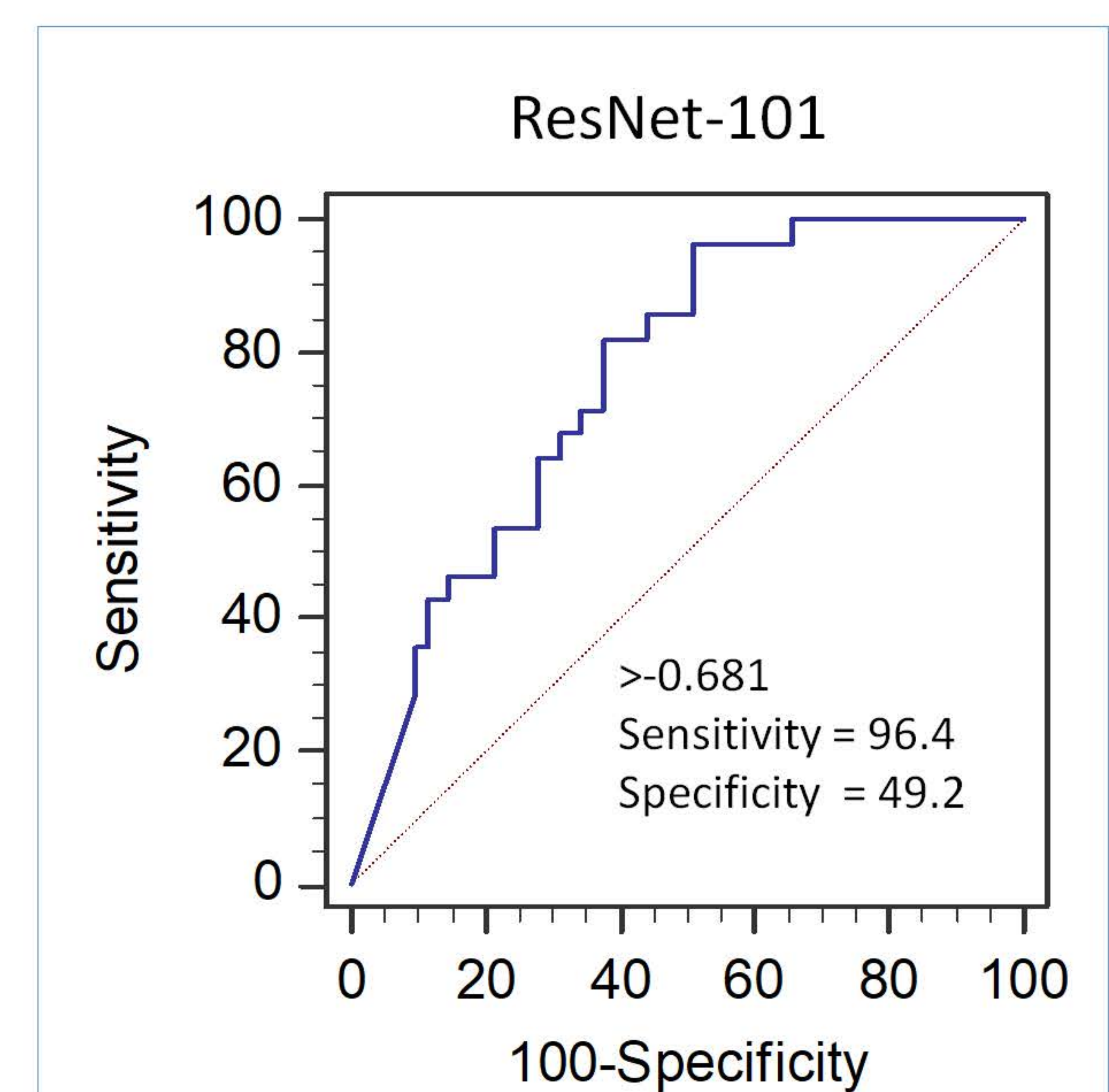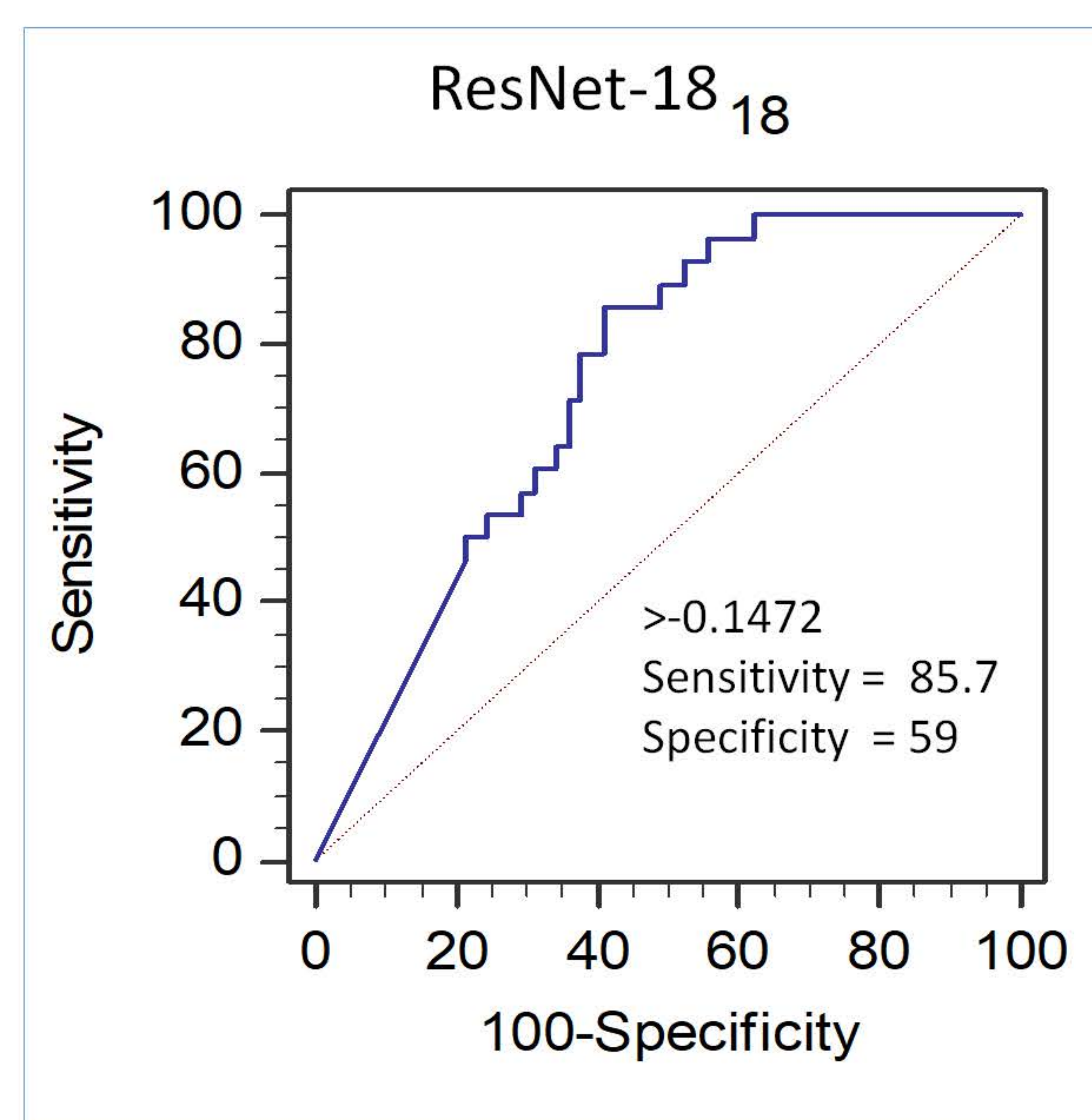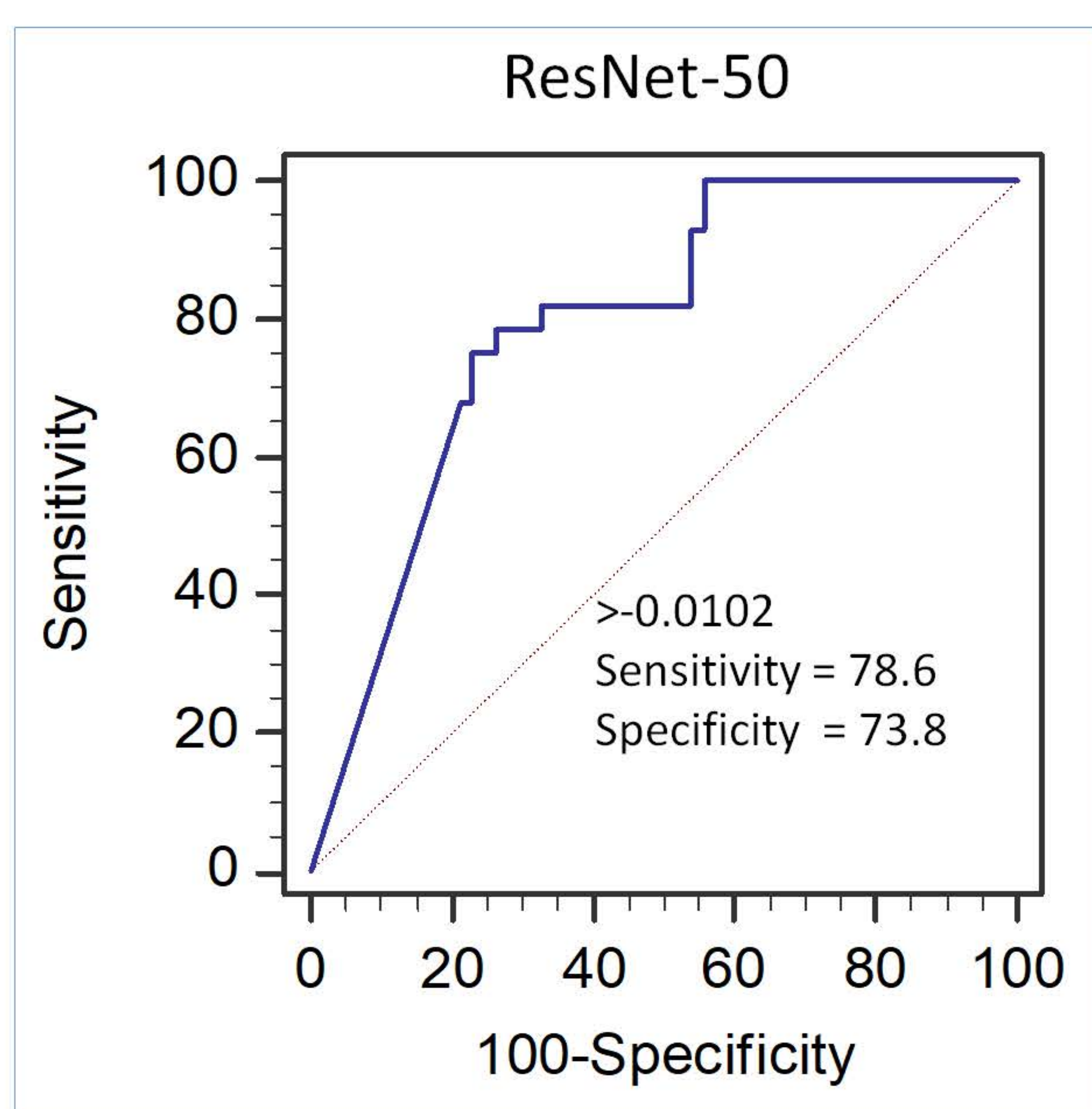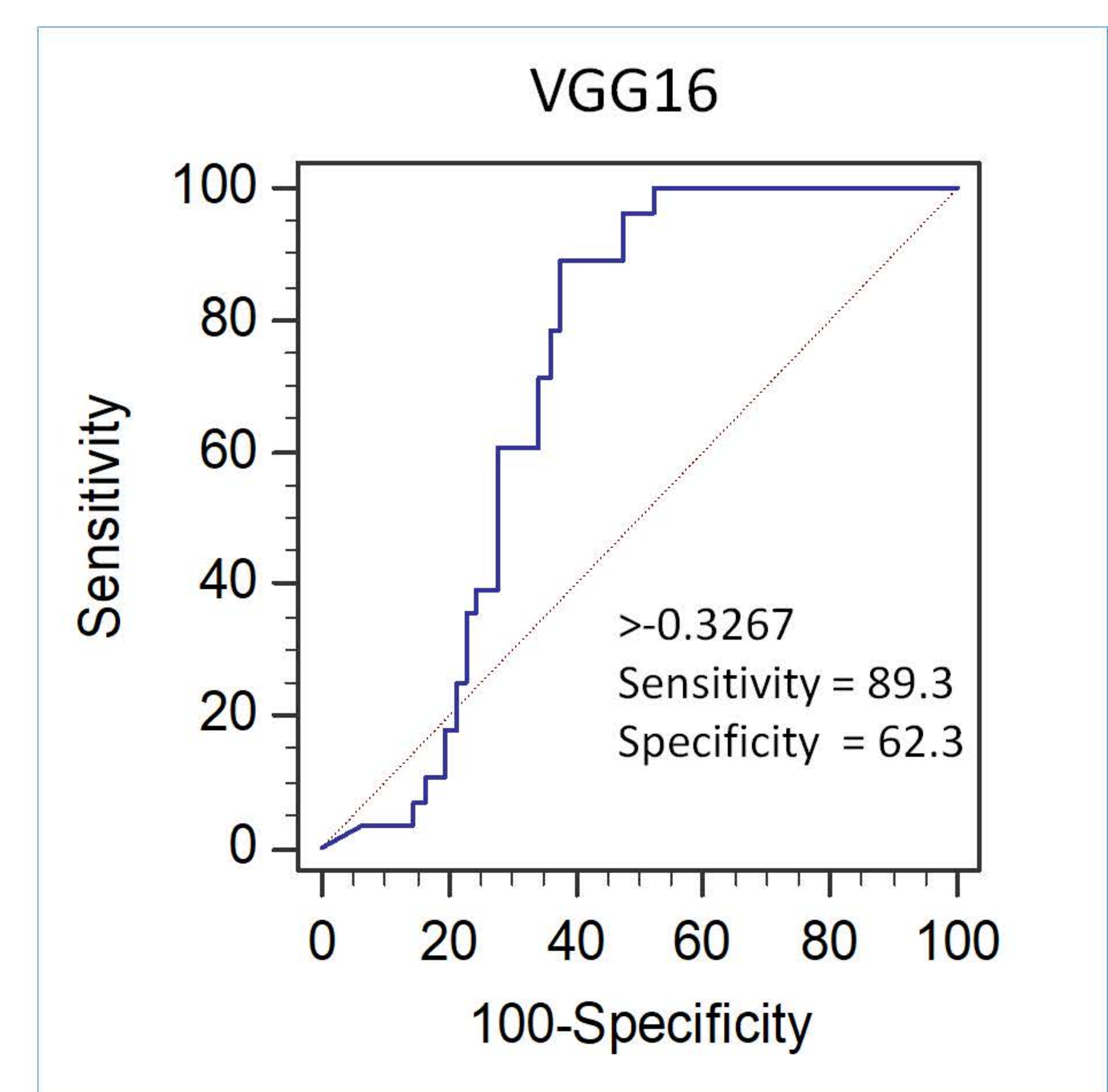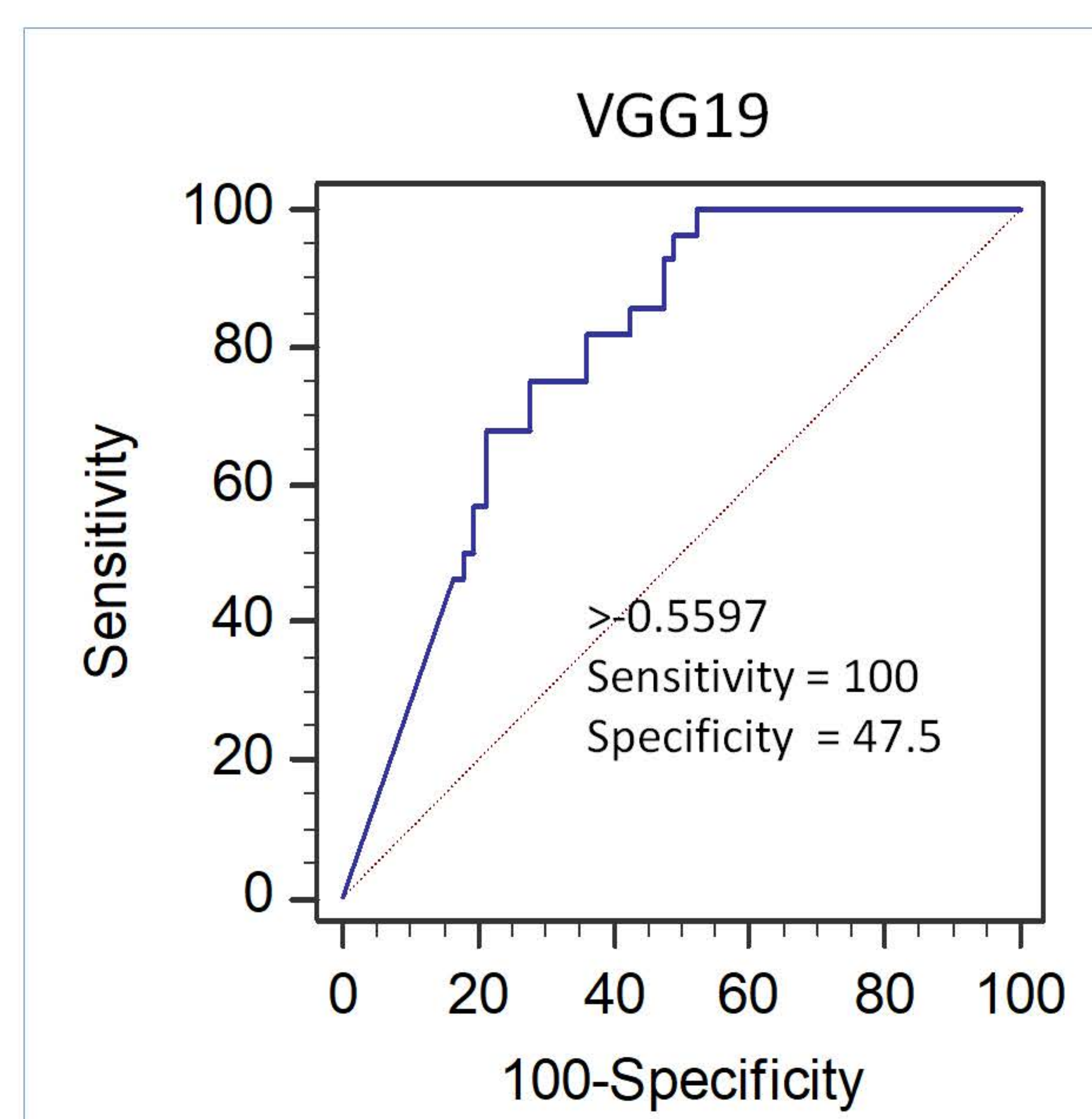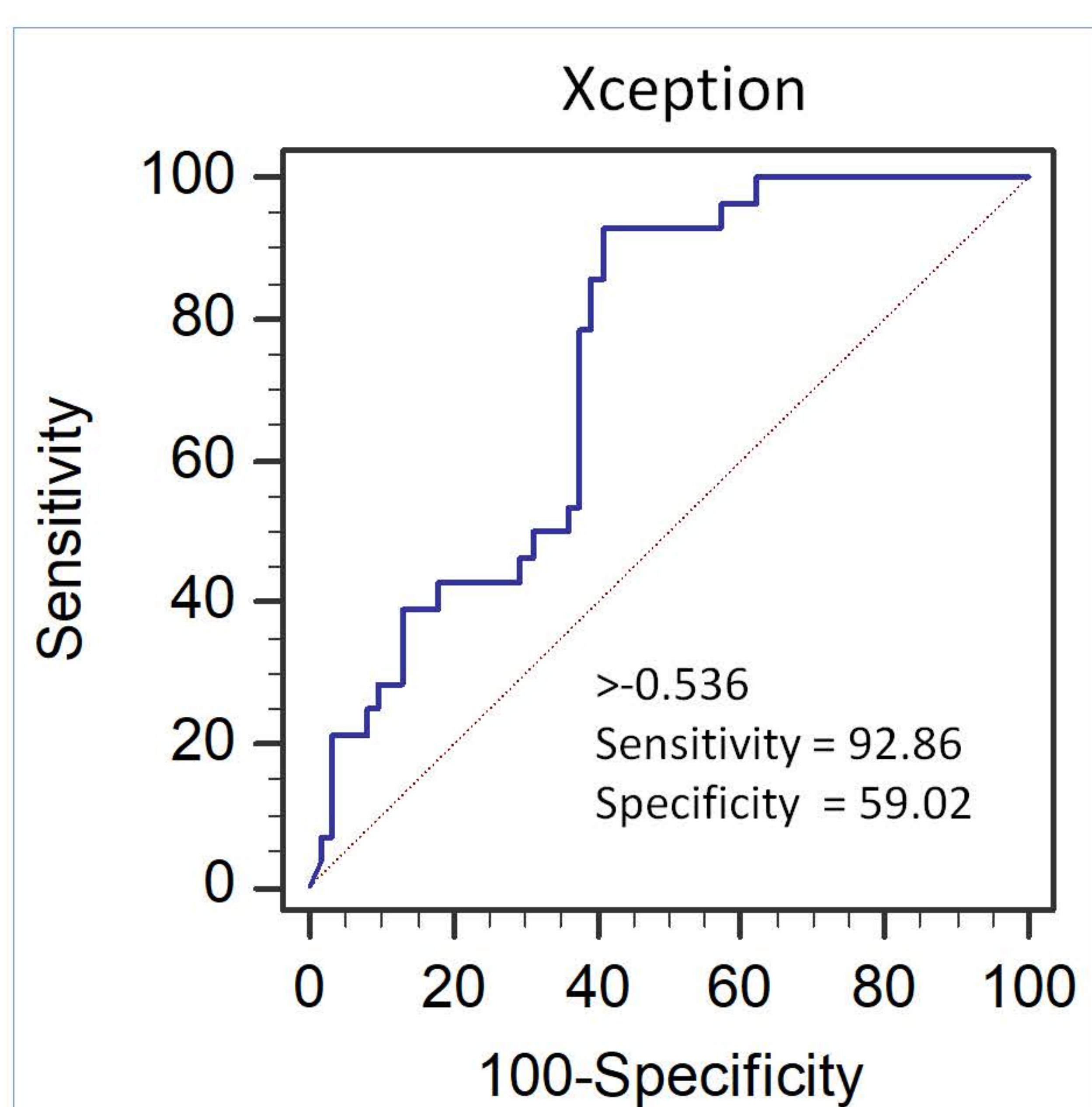

Supplement: Supplementary file 1 [file cancers-13-03583-s001.zip › Figure S7.pdf]

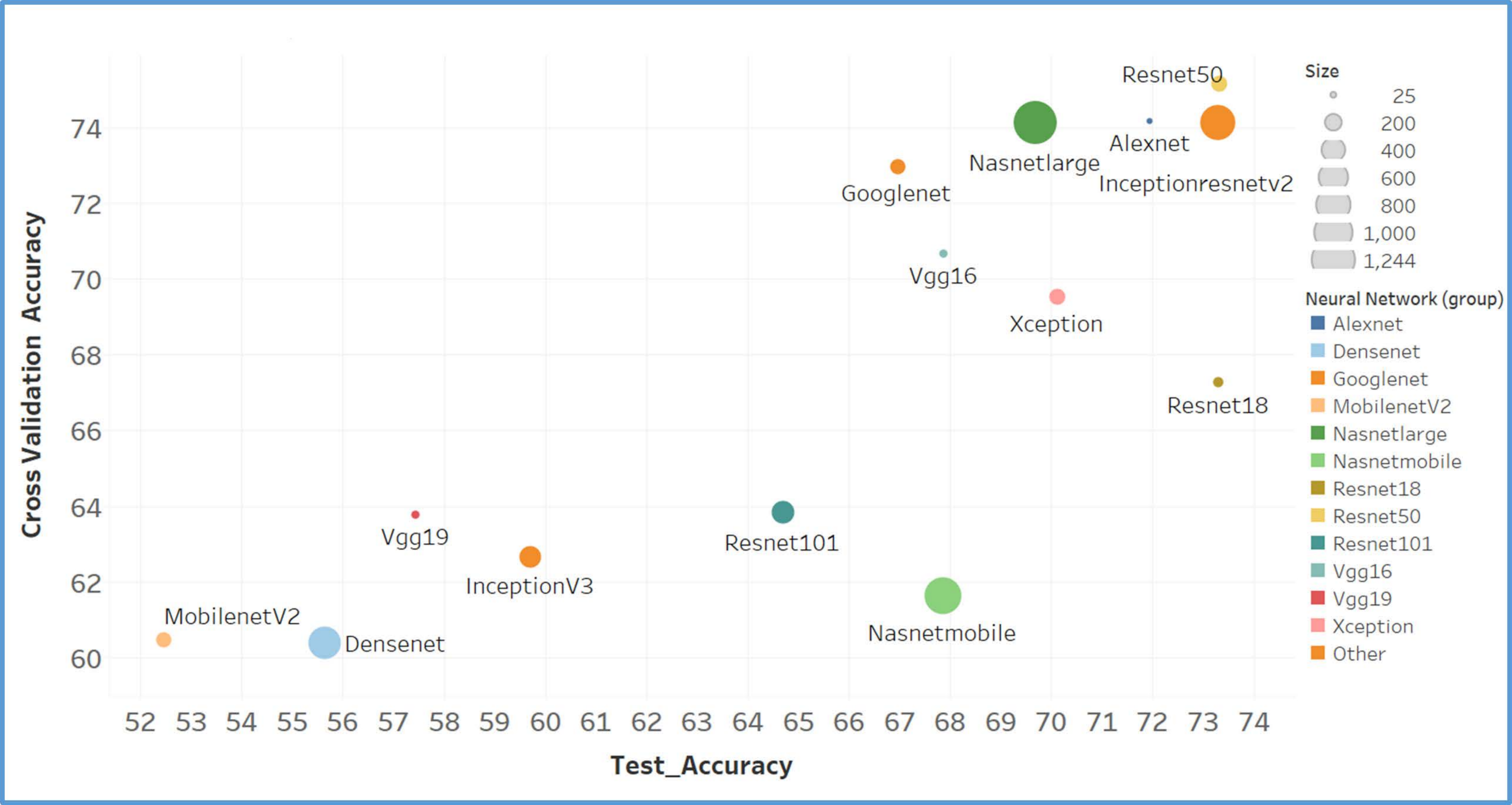

Supplement: Supplementary file 1 [file cancers-13-03583-s001.zip › Figure S8.pdf]
