# Supplementary material for: Validation of a Point-of-Care Optical Coherence Tomography Device with Machine Learning Algorithm for Detection of Oral Potentially Malignant and Malignant Lesions
Source: Cancers (Basel). 2021 Jul 17;13(14):3583. doi: 10.3390/cancers13143583 (PMC8304149; doi:10.3390/cancers13143583)
Supplement: Supplementary file 1 [file Figure S2.pdf › Figure S2.pdf]

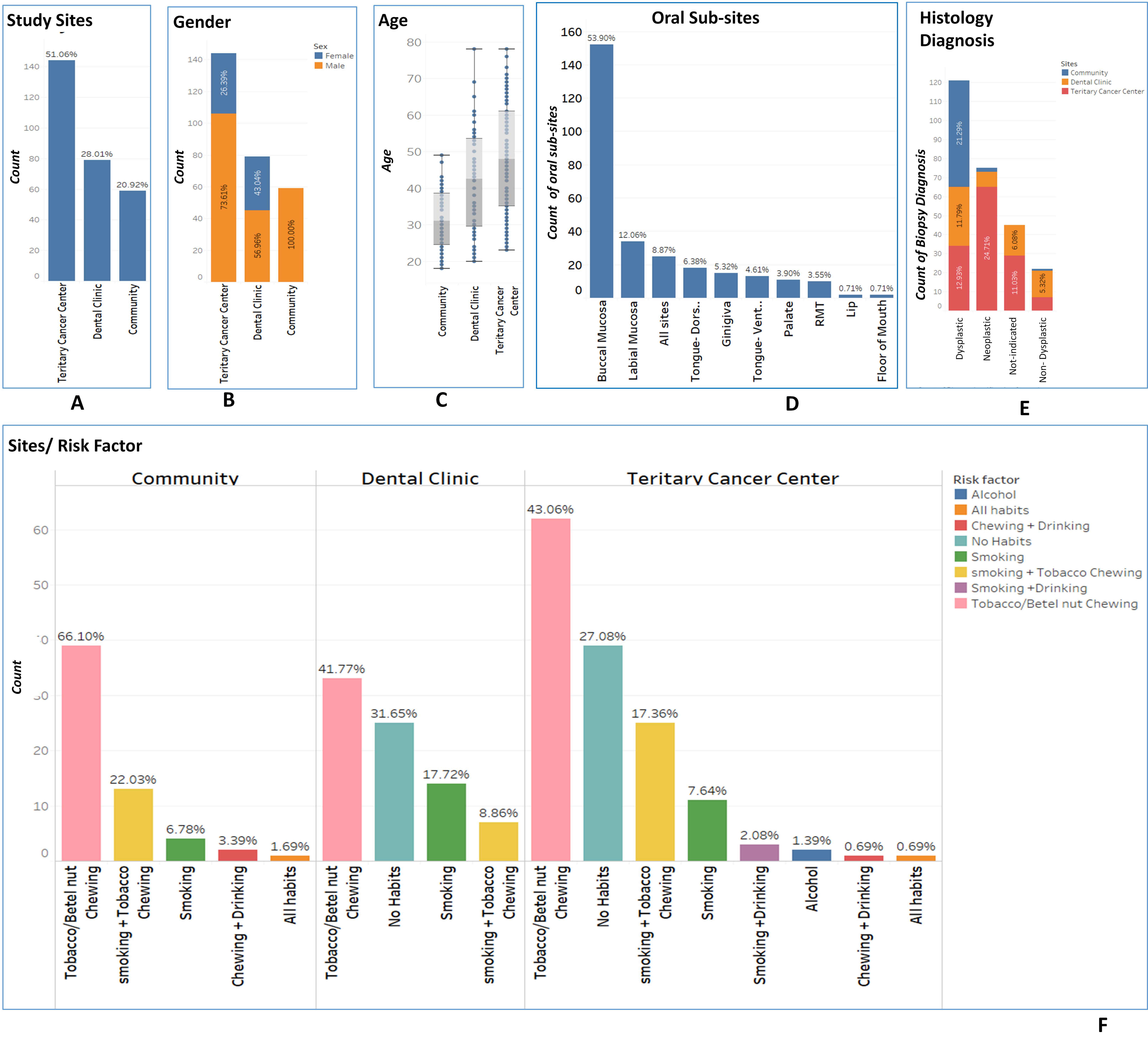
Study Sites
Count
51.06%
28.01%
20.92%
Teritary Cancer Center
Dental Clinic
Community
A
Gender
Sex
Female
Male
Count
26.39%
73.61%
43.04%
56.96%
100.00%
Teritary Cancer Center
Dental Clinic
Community
B
Age
Age
Community
Dental Clinic
Teritary Cancer Center
C
Oral Sub-sites
Count of oral sub-sites
53.90%
12.06%
8.87%
6.38%
5.32%
4.61%
3.90%
3.55%
0.71%
0.71%
Buccal Mucosa
Labial Mucosa
All sites
Tongue- Dors..
Ginigiva
Tongue- Vent..
Palate
RMT
Lip
Floor of Mouth
D
Histology Diagnosis
Sites
Community
Dental Clinic
Teritary Cancer Center
Count of Biopsy Diagnosis
21.29%
11.79%
12.93%
24.71%
6.08%
11.03%
5.32%
Dysplastic
Neoplastic
Not-indicated
Non- Dysplastic
E
Sites/ Risk Factor
Community
Dental Clinic
Teritary Cancer Center
Risk factor
Alcohol
All habits
Chewing + Drinking
No Habits
Smoking
smoking + Tobacco Chewing
Smoking +Drinking
Tobacco/Betel nut Chewing
Count
66.10%
22.03%
6.78%
3.39%
1.69%
41.77%
31.65%
17.72%
8.86%
43.06%
27.08%
17.36%
7.64%
2.08%
1.39%
0.69%
0.69%
Tobacco/Betel nut Chewing
smoking + Tobacco Chewing
Smoking
Chewing + Drinking
All habits
No Habits
Smoking+Drinking
Alcohol
F
