# Supplementary material for: Validation of a Point-of-Care Optical Coherence Tomography Device with Machine Learning Algorithm for Detection of Oral Potentially Malignant and Malignant Lesions
Source: Cancers (Basel). 2021 Jul 17;13(14):3583. doi: 10.3390/cancers13143583 (PMC8304149; doi:10.3390/cancers13143583)
Supplement: Supplementary file 1 [file Figure S9.pdf › Figure S9.pdf]

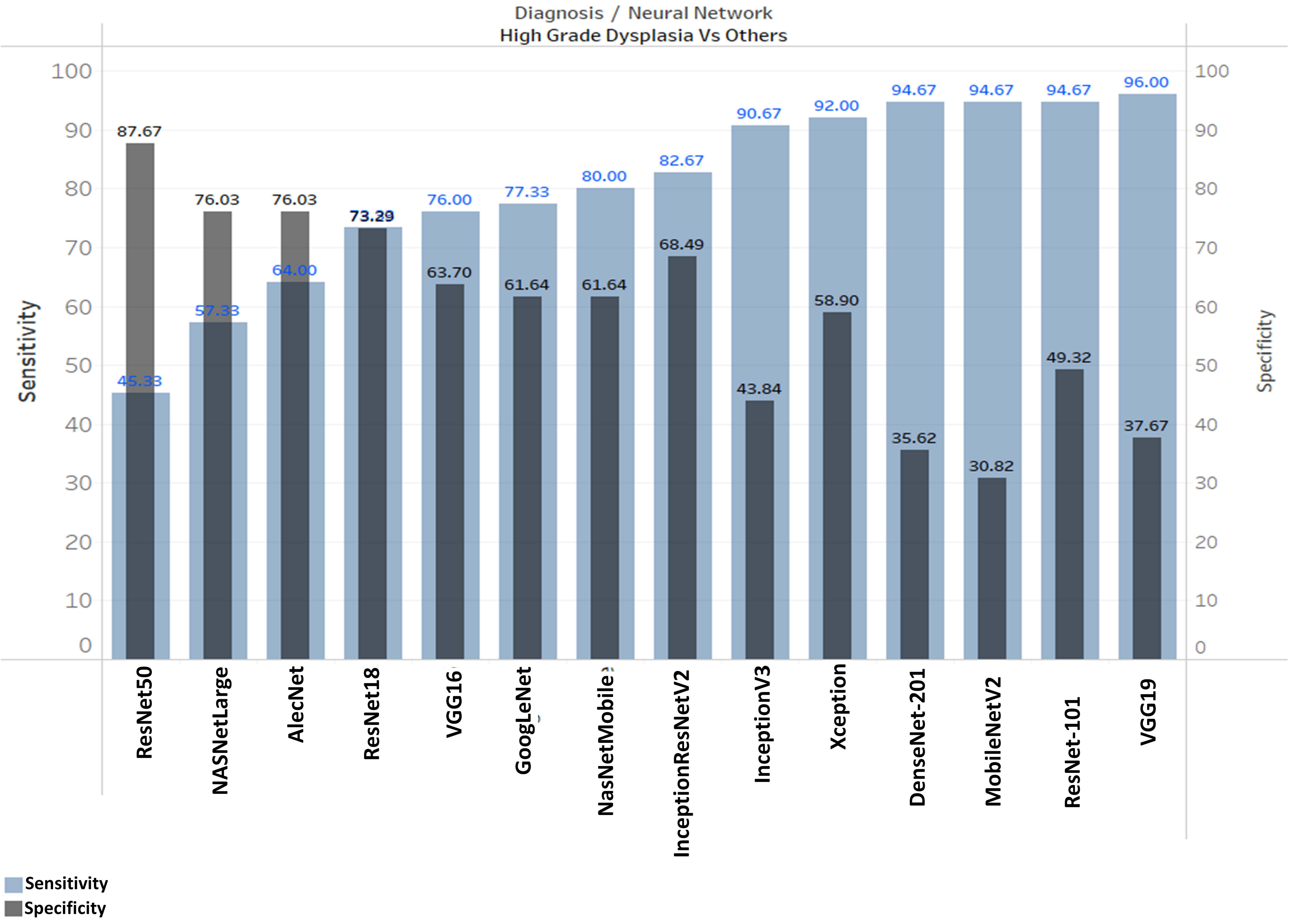

Diagnosis / Neural Network
High Grade Dysplasia Vs Others
Sensitivity
Specificity
100
90
80
70
60
50
40
30
20
10
0
87.67
45.33
76.03
57.33
76.03
64.00
73.29
76.00
63.70
77.33
61.64
80.00
61.64
82.67
68.49
90.67
43.84
92.00
58.90
94.67
35.62
94.67
30.82
94.67
49.32
96.00
37.67
ResNet50
NASNetLarge
AlecNet
ResNet18
VGG16
GoogLeNet
NasNetMobile
InceptionResNetV2
InceptionV3
Xception
DenseNet-201
MobileNetV2
ResNet-101
VGG19
Sensitivity
Specificity
